# Estimating the scale of chronic hepatitis C virus infection in the EU/EEA: a focus on migrants from anti-HCV endemic countries

**DOI:** 10.1186/s12879-017-2908-5

**Published:** 2018-01-16

**Authors:** A. M. Falla, A. A. Ahmad, E. Duffell, T. Noori, I. K. Veldhuijzen

**Affiliations:** 1 Department of Public Health, Erasmus MC, University Medical Center Rotterdam, Rotterdam, the Netherlands; 2 Division of Infectious Disease Control, Municipal Public Health Service Rotterdam-Rijnmond, PO Box 70032, 3000 LP Rotterdam, The Netherlands; 3 Department of Health Sciences, Hamburg University of Applied Sciences, Faculty Life Sciences / Public Health Research, Ulmenliet 20, 21033 Hamburg, Germany; 4 Department of Internal Medicine, University Medical Center Hamburg-Eppendorf, Martinistr 52, 20246 Hamburg, Germany; 5 European Centre for Disease Prevention and Control, Granits väg 8, 171 65 Solna, Sweden; 6 Center for Infectious Disease Control, National Institute for Public Health and the Environment, Bilthoven, the Netherlands

**Keywords:** Chronic viral hepatitis, Hepatitis C virus (HCV), Migrants, Epidemiology, Europe

## Abstract

**Background:**

Increasing the proportion diagnosed with and on treatment for chronic hepatitis C (CHC) is key to the elimination of hepatitis C in Europe. This study contributes to secondary prevention planning in the European Union/European Economic Area (EU/EEA) by estimating the number of CHC (anti-HCV positive and viraemic) cases among migrants living in the EU/EEA and born in endemic countries, defining the most affected migrant populations, and assessing whether country of birth prevalence is a reliable proxy for migrant prevalence.

**Methods:**

Migrant country of birth and population size extracted from statistical databases and anti-HCV prevalence in countries of birth and in EU/EEA countries derived from a systematic literature search were used to estimate caseload among and most affected migrants. Reliability of country of birth prevalence as a proxy for migrant prevalence was assessed via a systematic literature search.

**Results:**

Approximately 11% of the EU/EEA adult population is foreign-born, 79% of whom were born in endemic (anti-HCV prevalence ≥1%) countries. Anti-HCV/CHC prevalence in migrants from endemic countries residing in the EU/EEA is estimated at 2.3%/1.6%, corresponding to ~580,000 CHC infections or 14% of the CHC disease burden in the EU/EEA.

The highest number of cases is found among migrants from Romania and Russia (50–60,000 cases each) and migrants from Italy, Morocco, Pakistan, Poland and Ukraine (25–35,000 cases each). Ten studies reporting prevalence in migrants in Europe were identified; in seven of these estimates, prevalence was comparable with the country of birth prevalence and in three estimates it was lower.

**Discussion:**

Migrants are disproportionately affected by CHC, account for a considerable number of CHC infections in EU/EEA countries, and are an important population for targeted case finding and treatment. Limited data suggest that country of birth prevalence can be used as a proxy for the prevalence in migrants.

**Electronic supplementary material:**

The online version of this article (doi: 10.1186/s12879-017-2908-5) contains supplementary material, which is available to authorized users.

## Background

Chronic infection with the hepatitis C virus (HCV) is a global public health challenge and a leading cause of liver disease-related morbidity and mortality. The epidemiology remains poorly understood, however, and global, national and risk group-specific anti-HCV and viraemic prevalence estimates vary considerably. Recent studies suggest that between 105 million and 185 million people are anti-HCV positive worldwide and that global anti-HCV prevalence in adults could be as high as 2% [1, 2]. The Global Burden of Disease study estimated that chronic HCV (CHC) infection causes almost half a million deaths annually and is the 25th leading cause of death worldwide [3].

Chronic HCV infection affects the liver and has a mostly asymptomatic onset, but can lead to cirrhosis and hepatocellular carcinoma (HCC) decades later [4]. The asymptomatic nature of infection and the lack of adequate screening programmes means that the majority of people infected with CHC are unaware of their infection and only around one third of all estimated CHC infections in Europe have been diagnosed [5–7]. Effective antiviral treatment can prevent the development of cirrhosis and HCC and, with newer direct acting antivirals (DAAs) reporting cure rates in more than 90% of cases, [8] the elimination of HCV infection is now possible in Europe [9]. This will require continued primary prevention of new infections in parallel with expansion of secondary prevention through effective screening, linkage to care and treatment.

Primary prevention measures in Europe, including a safe blood supply, improved infection control practices and harm reduction programmes, have led to a significant reduction of HCV transmission in many countries and a mathematical modelling study shows incident cases are declining [10]. Incident data is not systematically collected and reported in most EU/EEEA countries hampering a good understanding of time trends although iatrogenic and nosocomial transmission is reported to be rare in most EU/EEA countries [11]. However, models predict that the peak in the mortality is yet to be reached [9, 10, 12]. An estimated 7.4 million people are anti-HCV positive in the European Union/European Economic area (EU/EEA), although prevalence varies from 0.9% in Western Europe to 3.3% in Eastern Europe [1, 13]. Deaths from viral hepatitis now exceed those from HIV and tuberculosis combined and latest published estimates show that 96,000 people die each year in EU/EEA countries from HBV and HCV-related liver disease [3]. Some populations are disproportionately affected and have a high prevalence of chronic infection. One such population is migrants born in high anti-HCV prevalence countries [10, 14–16], although estimates of the number of cases and the most affected migrant populations in Europe are lacking.

This epidemiological study seeks to inform targeted screening, linkage to care and treatment in the EU/EEA by: providing estimates, across and within all 31 EU/EEA countries, of the number of CHC cases among migrants from countries where anti-HCV prevalence is ≥1%; providing an estimate of the relative contribution of migrants to the overall burden of disease; and comparing the reported in-country of birth prevalence with that found among migrants living in European countries. In a sister paper to this, we conduct a similar analysis for chronic hepatitis B among migrants from endemic countries.

## Methods

**Fig. 1:**
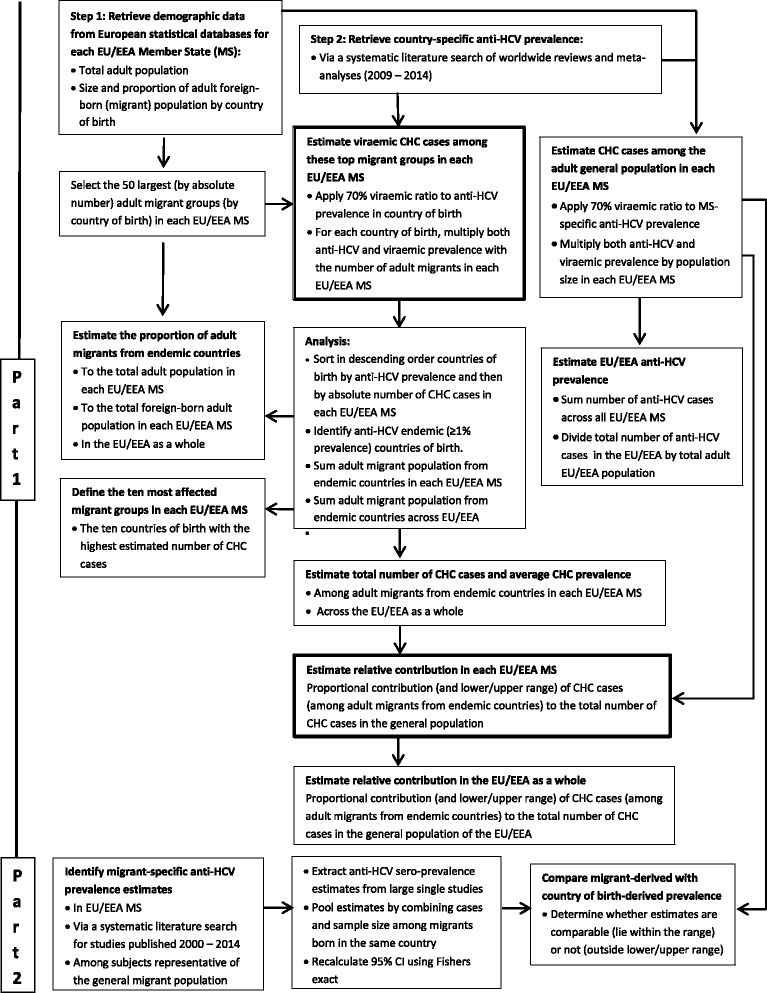
Schematic methodological representation to estimating the burden of CHC among migrants in the EU/EEA

The data retrieval and analysis process are described in detail below and in a schematic representation (Fig. 1). Demographic data on the size of and countries of birth of migrant populations were extracted from statistical databases. Country of birth-specific and EU/EEA country general population anti-HCV prevalence estimates were derived from a systematic literature search. To assess the reliability of using country of birth-derived prevalence as a proxy for the prevalence among migrants, a systematic literature search was conducted to identify prevalence estimates among migrants in Europe to compare with country of birth-derived prevalence.

### Definitions

#### Migrant

An adult 15 years old or above, born in a country other than the current country of residence. Children are excluded due to the dominance of older age populations in sero-prevalence studies and the higher prevalence reported among adults than among children [1]. The use of the term ‘migrant’ in this study therefore refers to adult (foreign-born) migrants only. The study accounts for and includes migration from outside the EU/EEA and migration within the EU/EEA, but excludes undocumented migrants.

#### Anti-HCV prevalence

The common measure of exposure to HCV/endemicity used in sero-prevalence studies. Anti-HCV prevalence can include exposed individuals with a resolved infection.

#### Chronic hepatitis C (CHC)

Refers to viraemic infection, i.e. anti-HCV and HCV-RNA positivity. Endemic country: the anti-HCV prevalence in the general adult population is ≥1%. This relatively low threshold was chosen to take into account migrants from countries where prevalence is higher than that of the EU/EEA as a whole.

### Part 1: The contribution of migrants from endemic countries to the burden of CHC in the EU/EEA

#### Demographic data (Step 1)

The size and country of birth of the migrant population was obtained for all 31 EU/EEA countries from Eurostat for 2013, if available [17]. Where data were unavailable, either the ‘EU 2011 – Housing and Population Census’, the most recent demographic data from the Organisation for Economic Co-operation and Development (OECD) Stats website or national statistics were used, in that order [18–20]. The data source is indicated in footnotes in Table 1. For each EU/EEA country, the countries of birth of migrants were sorted into descending order of magnitude of the number of migrants, and the top 50 countries of birth by migrant population size were then selected for estimating the CHC burden.

**Table 1:** Demographic data and CHC epidemiology in the general population and among migrants in EU/EEA countries

Country (anti-HCV prevalence; range)	General Adult Population				Migrants from endemic countries						
	Total Population^a^	Estimated no. of CHC cases			Number	Proportion (of the total population)	Estimated number of CHC cases			CHC prevalence	Relative contribution (and range) to burden of CHC
		Central estimate	Lower estimate	Upper estimate			Central Estimate	Lower Estimate	Upper Estimate		
Austria (0.5%; 0.1–0.7)	7,232,026	25,312	5062	35,437	883,345	12.2%	11,753	8243	15,117	1.3%	46% (15% - 77%)
Belgium (0.9%; 0.1–1.2)	9,263,570	58,360	6484	77,814	1,054,155	11.4%	18,607	9729	32,764	1.8%	32% (4% - 60%)
Bulgaria (1.1%; 0.3–2.4)	6,294,563	48,468	13,219	105,749	62,941	1.0%	1366	605	1836	2.2%	3% (0% - 6%)
Croatia (1.3%; 1.1–1.6)	3632,461^b,c^	33,055	27,970	40,684	524,609	14.4%	4901	4058	6081	0.9%	15% (10% - 22%)
Cyprus (0.6%; 0.5–0.7)	705,459^b^	2963	2469	9383	128,712	18.2%	2740	1821	3567	2.1%	92% (<0% - >100%)^f^
Czech Republic (0.7%; 0.2–0.7)	8,955,829	43,884	12,538	43,884	331,840	3.7%	5937	2596	8219	1.8%	14% (6% – 22%)
Denmark (0.7%; (0.5–0.7)	4,625,032	22,663	16,188	22,663	278,041	6.0%	3894	2244	5194	1.4%	17% (10% - 24%)
Estonia (3.3%; 1.6–4.5)	1,113,355	25,719	12,470	35,071	189,495	17.0%	5090	1625	7033	2.7%	20% (6% - 33%)
Finland (0.7%; 0.6–0.9)	4,535,282	22,223	19,048	28,572	176,980	3.9%	3383	1682	4826	1.9%	15% (7% - 23%)
France (0.7%) (0.5–0.8)	52,901,411^b^	259,217	185,155	296,248	5,714,076	10.8%	88,799	37,816	154,348	1.6%	34% (11% - 58%)
Germany (0.5%; 0.3–0.9)	69,414,404^b^	242,950	145,770	437,311	8,888,710	12.8%	128,809	61,796	193,947	1.4%	53% (11% - 95%)
Greece (1.9%; 0.5–2.6)	9464,000^c^	125,871	33,124	172,245	588,275	6.2%	12,959	9854	15,519	2.2%	10% (4% -16%)
Hungary (0.8%; 0.4–2.7)	8,477,933	47,476	23,738	160,233	336,360	4.0%	6548	4980	7889	1.9%	14% (0% - 34%)
Iceland (0.9%; 0.7–1.5)	255,391	1609	1251	2682	18,311	7.2%	206	111	307	1.1%	13% (4% - 21%)
Ireland (1.1%; 0.7–1.6)	3,586,829	27,619	17,575	40,172	321,771	9.0%	5485	2934	8188	1.7%	20% (7% - 32%)
Italy (4.4%; 1.6–7.3)	51,336,889	1,581,176	574,973	2,623,315	4,118,015	8.0%	78,501	52,730	101,393	1.9%	5% (1% - 9%)
Latvia (2.4%; 1.7–3.3)	1,731,509	29,089	20,605	39,998	271,468	15.7%	6532	2168	9209	2.4%	22% (8% - 37%)
Liechtenstein (0.9%; 0.7–1.5)	31,142	196	153	327	14,672	47.1%	172	82	223	1.2%	88% (35% - >100%)^f^
Lithuania (2.9%; 0.7–3.0)	2,535,329 ^e^	51,467	12,423	53,242	127,711	5.0%	2795	1046	4142	2.2%	5% (2%- 9%)
Luxembourg (0.9%; 0.6–0.9)	417,377^d^	2629	1753	2629	104,495	25.0%	1682	659	2710	1.6%	64% (24% - >100%)^f^
Malta (0.9%; 0.7–1.5)	355,704^b^	2241	1743	3735	17,833	5.0%	295	182	438	1.7%	13% (5% - 21%)
Netherlands (0.2%; 0.1–0.4)	13,901,653	19,462	9731	38,925	978,698	7.0%	13,262	7278	18,376	1.4%	68% (10% - >100%)^f^
Norway (0.7%; (0.6–0.9)	4,122,334	20,199	17,314	25,971	357,877	8.7%	4822	2601	6907	1.3%	24% (12% - 36%)
Poland (1.1%; 0.6–1.9)	32,736,685	252,072	137,494	435,398	453,723	1.4%	9633	3080	12,877	2.1%	4% (1% - 7%)
Portugal (1.8%; 0.5–2.9)	8989,849^b^	113,272	31,464	182,494	642,212	7.1%	13,505	7474	24,179	2.1%	12% (1% - 23%)
Romania (3.2%; 2.9–3.6)	16,880,465	378,122	342,673	425,388	102,854	0.6%	2090	912	2923	2.0%	1% (0% - 1%)
Slovakia (1.4%; 0.9–2.0)	4,580,260	44,887	28,856	64,124	31,460	0.7%	588	281	782	1.9%	1% (0% - 2%)
Slovenia (1.3%; 1.1–1.6)	1,760,726	16,023	13,558	19,720	208,026	11.8%	2030	1607	2563	1.0%	13% (9% - 17%)
Spain (1.7%; 0.4–2.6)	39,637,891	471,691	110,986	721,410	3,748,924	9.5%	55,164	31,440	75,323	1.5%	12% (3% - 21%)
Sweden (0.6%; 0.5–0.7)	7,944,034	33,365	27,804	38,926	773,085	9.7%	10,579	5352	14,452	1.4%	32% (17% - 46%)
United Kingdom (0.6%; (0.4–1.2)	52,082,285^b^	218,746	145,830	437,491	4,706,765	9.0%	76,535	45,555	118,608	1.6%	35% (6% -64%)
EU/EEA (1.4%; 0.5–1.5)	429,501,677	4,222,026	1,999,421	6,621,241	36,155,438	8.4%	578,663	312,539	859,941	1.6%	14% (4% - 24%)

^a^Source is EUROSTAT 2013 unless indicated by the following letter

^b^ESS 2011 Census

^c^OECD 2012/EUROSTAT 2013 age distribution

^d^OECD 2010/EUROSTAT age distribution

^e^http://www.euras.lt (Lithuanian National Statistics Agency)/EUROSTAT 2013 age distribution

^f^The Delta method does not give a reliable confidence interval around the relative contribution as the relative contribution is close to 100% and the distribution of cases in the general population is extremely skewed

#### Country-specific anti-HCV prevalence (Step 2)

The online databases Medline, Embase, the Cochrane library, Web of Science, Scopus, PubMed publisher and Google Scholar were searched in January 2015 for reviews, systematic reviews and meta-analyses in English about the prevalence of hepatitis C in the general population at country level. The search (described in full in Annex 1 of the Additional file 1) used a combination of disease-related (hepatitis C), outcome-related (prevalence), population-related (general population, worldwide) and study design-related (reviews) terms. Note that the search also included terms related to hepatitis B since we conducted a similar analysis for chronic hepatitis B among migrants from endemic countries (to be published in this journal). Since the aim was to identify recent reviews, the search was restricted to papers published after 2009 to the date of the search. The titles and abstracts, then the full text, of retrieved citations were assessed for relevancy by one reviewer (AF). Key exclusion criteria included studies focused on: hepatitis other than type C; natural history, clinical features or complications of hepatitis; medical treatment; other high risk groups e.g. people who inject drugs (PWID); and single case studies and cost effectiveness analyses. High quality systematic reviews/meta-analyses were selected given the recent publication of robust systematic reviews/meta-analyses of national level prevalence estimates globally.

Country-level anti-HCV prevalence estimates and uncertainty ranges/confidence intervals (CIs) were extracted from the included reviews and entered into a Microsoft Excel database of all countries. Where a country-specific estimate was unavailable, the relevant Global Burden of Disease region estimate was used. If a meta-analysis reported a statistically significant time trend, the estimate from the most recent period was selected. Where multiple estimates for a country were available from different reviews, the most robust was selected based on the assessed risk of selection bias. Risk of selection bias was assessed based on: sampling method (random favoured over convenience); sampling population (the general population was favoured over more specifically defined (risk) groups); geographical coverage (national favoured over regional; regional favoured over local); sample size (larger studies preferred over smaller one); and data collection timeframe (favouring recency). Decisions were made jointly by two reviewers (AF and IV) based on these criteria (rather than a pre-defined algorithm) with a detailed rationale recorded for each selected estimate. This rationale, together with search strategy, the inclusion/exclusion criteria and a PRISMA flowchart are available in the online supplement.

#### Estimating the CHC burden among migrants from endemic countries

Anti-HCV prevalence was multiplied by the number of migrants from the top 50 countries of birth of migrants in each EU/EEA country. The countries of birth were then sorted in descending order of magnitude by anti-HCV prevalence to identify all endemic (≥1% anti-HCV prevalence) countries. The total number of migrants born in anti-HCV endemic countries was used to determine both the total and proportional contribution of migrants from these countries to the overall number of migrants residing in each of the 31 EU/EEA countries. To estimate the proportion of CHC (viraemic) cases among the anti-HCV positive migrant population, the worldwide average viraemic proportion of 70% found in a recent global meta-analysis was applied [1].

#### Relative contribution

For each EU/EEA country, the estimated number of infected cases among migrants from endemic countries was divided by the total number of infected persons (based on the general population CHC prevalence estimate and the total population) to estimate the relative contribution of migrants from endemic countries to the overall number of people infected with CHC. Given uncertainty in both the size of the migrant population and CHC prevalence estimates in countries of birth, a range in the relative contribution (a lower limit and a higher limit) was also calculated using the Delta method [21].

### Part 2: Anti-HCV prevalence in migrant populations in Europe

The online databases Medline, Embase, the Cochrane library, Web of Science, Scopus, PubMed publisher and Google Scholar were searched in November 2014 for studies in English that estimate the prevalence of hepatitis C in migrants in any of the 31 EU/EEA countries. The search used a combination of disease-related (hepatitis C), outcome-related (prevalence), population-related (migrants) and geographical area (EU/EEA countries) terms and was limited to studies published between 2000 and 2014. We expected a limited retrieval from this search and therefore included only selection bias (how representative the study population was of the general population) as a key parameter in the risk of bias assessment. This operationalised through the exclusion of studies sampled from higher risk migrant groups such as refugees/asylum seeker and higher risk (general) populations such as STI clinic attendees, outpatient clinics, international health centres etc. The full search strategy, inclusion/exclusion criteria and PRISMA flowchart are available in the online supplement.

Data from the included studies were entered into Microsoft Excel. Pooled estimates for countries of birth were produced by combining the numbers tested and the number of cases. A 95% CI was re-calculated using the Fisher’s exact method. Prevalence estimates pooled from multiple studies or extracted from large single studies (>25 subjects from a single country) were compared with the in-country estimates to determine whether in-country estimates reflect the prevalence found among migrants. When the point prevalence from a study in migrants (Part 2) fell within the CI/uncertainty range of the in-country estimate (from Part 1), this estimate was considered to be comparable; when it fell below the lower CI/uncertainty range, it was considered to be lower than the in-country prevalence; and when it was higher than the upper CI/uncertainty range, it was considered to be higher.

## Results

### Estimated CHC prevalence and number of infected cases in 31 EU/EEA countries (Table 1)

The anti-HCV prevalence in the general population in the EU/EEA is estimated at 1.4% (range of 0.7–2.2%). However, prevalence estimates range from 0.2% in the Netherlands to 4.4% in Italy and 14 EU/EEA countries are considered endemic by the definition adopted in our study (≥1% anti-HCV prevalence). Table 1 lists the estimated number and range of CHC cases among adults in EU/EEA countries. An estimated 4.2 million (range 2.0–6.6 million) adults in the EU/EEA have CHC infection. Italy has the highest absolute number, with an estimated 1.6 million CHC cases. Other EU/EEA countries with a high absolute number of CHC cases among adults are Romania, with 380,000, and Spain, with 470,000.

### The distribution of migrants in the EU/EEA based on HCV endemicity in country of birth

**Fig. 2:**
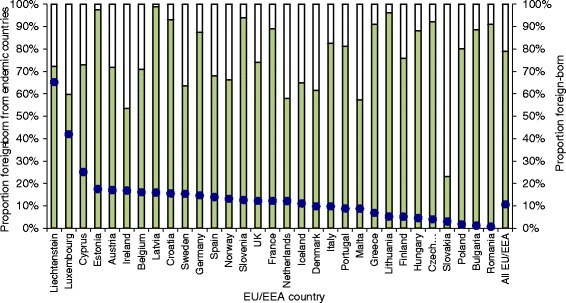
Total (%) migrant population in each EU/EEA country and the proportion born in endemic countries

The top 50 migrant populations in each EU/EEA country included in the analysis make up at least 95% of the total migrant population in 19 countries and at least 90% in all but three EU/EEA countries (Denmark, Sweden and the United Kingdom, where the proportion is at least 85%). These migrant populations account for approximately 10.7% of the total adult population in the EU/EEA although the proportion in each country varies, ranging from 0.7% in Romania, 1.1% in Bulgaria and 1.7% in Poland to 42.0% in Luxembourg and 65.2% in Liechtenstein (Fig. 2).

Nearly 80% of the total migrant population were born in HCV endemic countries. Other than in Slovakia, where 23% of the total migrant population are from endemic countries, over half of all migrants in the other 30 EU/EEA countries were born in countries where the anti-HCV prevalence is ≥1%. In Croatia, Estonia, Latvia, Lithuania, Romania and Slovenia, more than 90% of all migrants are from endemic countries (Fig. 2). The number and proportion of all migrants that from endemic countries, at country level and in the EU/EEA as a whole are shown in Table 2.

**Table 2:** The ten migrant groups (from endemic countries) accounting for the highest number of CHC cases

Migrant country of birth	Total adult migrant population	Anti-HCV prevalence	Number (rounded) of CHC cases	Host countries (first 6 with largest populations)^a^
Romania	2,646,392	3.2	59,000	Italy, Spain, Germany, Hungary, UK, Austria
Russia	1,713,636	4.1	49,000	Germany, Latvia, Estonia, Italy, Lithuania, Spain
Italy	1,114,683	4.4	34,000	France, Germany, UK, Belgium, Spain, The Netherlands
Poland	4,103,409	1.1	32,000	Germany, UK, Italy, France, Ireland, The Netherlands
Morocco	2,418,072	1.6	27,000	France, Spain, Italy, Belgium, The Netherlands, Germany
Pakistan	756,170	5.0	27,000	UK, Italy, Spain, Germany, Greece, France
Ukraine	993,459	3.6	25,000	Poland, Germany, Italy, Czech Republic, Spain, Latvia
Egypt	194,852	15.7	21,000	Italy, UK, France, The Netherlands, Austria, Greece
Kazakhstan	807,781	3.3	19,000	Germany, Latvia, Czech Republic, Poland, Lithuania, Estonia
Nigeria	313,212	8.4	18,000	UK, Italy, Spain, Ireland, Austria, The Netherlands

^a^if migrant population is at least 1000

### Country-specific anti-HCV prevalence estimates

The most comprehensive review with country-specific estimates of anti-HCV prevalence identified by the search was published in 2014 by Gower et al. [1]. This review includes studies published after the year 2000 and provides estimates for 87 countries and for each of the 21 Global Burden of Disease regions. The country-level estimates from Gower do not have 95% CIs but a lower and upper uncertainty range; the lower range is based on studies among populations considered representative of ‘healthy adults’ (such as blood donors), but the methodology applied to derive the upper limit is unclear. For nine countries, Gower’s regional or in-country estimate was replaced with estimates from other systematic reviews deemed more robust according to the criteria described in the methods [2, 22–26]. The 224 country-level prevalence estimates and the source, together with an overview of decision rationale where an estimate other than Gower was available, are listed in the online supplement.

### Estimated prevalence and number of CHC infections among migrants

Across the EU/EEA, the overall anti-HCV prevalence among migrants from endemic countries is estimated at 2.3%, which corresponds to a CHC prevalence of 1.6% and an estimated 580,000 CHC infections (Table 2). The estimated prevalence of CHC infection among migrants from endemic countries ranges from 0.9% in Croatia to 2.4% in Estonia. Table 2 lists the ten migrant populations with the highest estimated number of CHC cases and the host EU/EEA countries with the largest populations of migrants born in these countries. Based on cumulative analysis of the CHC burden among the different migrant populations from endemic countries to or within the EU, migrants from Romania, Russia, Italy and Poland contribute most, in descending order, to the overall number of CHC cases. An estimated 50,000–60,000 CHC cases are found among migrants from Romania and from Russia.

Some countries of birth of migrants are common across EU/EEA countries. Adult migrants from Russia, a high CHC prevalence country (2.9%), are represented among the top ten migrant populations in 25 of 31 EU/EEA countries. Migrants from Romania and Italy are among the top ten migrant groups in 20 EU/EEA countries. Although small in number, migrants from Egypt are among the top ten CHC-infected migrant populations in 16 of 31 EU/EEA countries due the very high anti-HCV prevalence (15.7%) in Egypt. In Estonia, Lithuania and Latvia, the top ten migrant populations with the highest number of infected cases are all from countries of the former Soviet Union. This is also the case in five to six of the top ten migrant populations with the highest number of infected cases in Bulgaria, the Czech Republic and Poland. People born either in Yugoslavia before 1992 or in one of the countries that emerged from the fall of Yugoslavia since 1992are represented in six of the top ten migrant populations with the largest of number of CHC cases in Croatia and Slovenia and three of the top ten in Austria. Migrants from the Algeria, Morocco and Tunisia are represented among the ten most CHC-affected migrant populations in France. EU/EEA countries with three to five African countries represented among the top ten CHC-affected migrant groups include Belgium, France, Luxembourg, Portugal and the UK. Prevalence, population size and number and range of CHC infections among, for all 50 countries of birth of migrants in each EU/EEA country can be found in the online supplement.

### Relative contribution of migrants to the CHC burden in EU/EEA countries

**Fig. 3:**
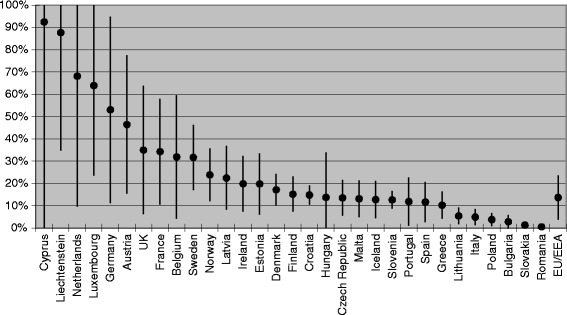
Estimated relative contribution (upper/lower range) of migrants to the total number of CHC cases

The relative proportion (and range) of infected migrants from endemic countries within the overall CHC burden in EU/EEA countries is shown in Table 1 and Fig. 3. The relative contribution is low (<4%) in Bulgaria, Poland, Romania and Slovakia and much higher (64%–92%) in Cyprus, Liechtenstein, Luxembourg and the Netherlands.

### Comparing migrant-derived anti-HCV prevalence with country of birth estimates

The literature search identified thirteen anti-HCV prevalence estimates from studies in migrants in the EU/EEA for comparison with the in-country estimates derived in Part 1. Two of the thirteen estimates, from studies among migrants from the former Dutch Antilles and Suriname, were higher than the comparator regional prevalence [1]. One estimate, from a study among migrants from the former Soviet Union, could not be compared with in-country anti-HCV prevalence since this nation state is now dissolved. Of the remaining ten estimates, seven were comparable with the in-country estimate and three, among migrants from Egypt, Pakistan and Turkey, were lower than the in-country prevalence (Egypt: 2.4% in migrants vs. 15.7% in-country; Pakistan: 2.8% in migrants vs. 5.5% in-country; and Turkey: 0.2% in migrants vs. 1.0% in-country), although for Turkey the two confidence intervals overlap. See Table 3 for details.

**Table 3:** Comparing migrant study-derived prevalence to country of birth-derived prevalence

Country of birth	Migrants			In-country		Comparison
	N. tested	Prevalence (95% CI)	Reference	Prevalence (95% CI)	Reference	
Afghanistan	293	1.0 (0.2–3.0)	[32]	1.1 (0.6–1.9)	[1]	Comparable
Bangladesh	934	0.4 (0.1–1.1)	[52, 53]	1.3 (0.2–2.2)	[1]	Comparable
Dutch Antilles	38	2.6 (0.1–13.8)	[15, 45]	0.8^b^ (0.2–1.3)	[1]	Higher
Egypt	465	2.4 (1.2–4.2)	[54]	15.7 (13.9–17.5)	[25]	Lower
Former USSR	65	3.1 (0.4–10.7)	[32]	3.3^a^ (1.6–4.5)	[1]	Comparable
India	1334	0.4 (0.2–1.0)	[52, 53]	0.8 (0.4–1.0)	[1]	Comparable
Iran	153	0.7 (0–3.6)	[32]	0.5 (0.2 – 1^a^)	[1]	Comparable
Iraq	290	0.3 (0–1.9)	[32]	3.2 (0.3–3.2^a^)	[1]	Comparable
Morocco	331	0.9 (0.2–2.6)	[15, 45]	1.6 (0.6–1.9^a^)	[1]	Comparable
Pakistan	3562	2.8 (2.3–3.4)	[30, 52, 53]	5.5 (4.4–5.5)	[26]	Lower
Suriname	225	2.4 (0.5–7.0)	[15, 45]	0.8^b^ (0.2–1.3)	[1]	Higher
Turkey	965	0.2 (0–0.8)	[15, 45, 46]	1.0 (0.7–1.1)	[22]	Lower
Vietnam	126	1.6 (0.2–5.6)	[32]	1.0 (0.8–1.8)	[1]	Comparable

^a^Regional estimate from GBD Eastern European region

^b^Caribbean GBD Regional estimate

## Discussion

This is the first study we know of that attempts to systematically estimate the overall number of CHC cases among migrants, as well as the relative contribution of cases among migrants to the overall burden of CHC in EU/EEA countries. Migrants from endemic countries account for one in 12 EU/EEA adult citizens and for one in seven of all CHC cases in the EU/EEA. As the contribution of migrants to the overall burden of CHC varies between EU/EEA countries, effective approaches to secondary prevention will, therefore, also differ. Screening programmes targeting migrant populations will be most effective in EU/EEA countries such as Austria, France, Germany, the Netherlands and the UK where migrants account for a large proportion of the disease burden (32–92%, see Fig. 3) and a small proportion of the total population (7–15%, see Fig. 2).

In contrast, in countries where HCV prevalence is high in the general population and the contribution of migrants is low, it may be more cost-effective to implement population-based screening [27, 28]. Examples of such countries include Bulgaria, Poland, Romania and Slovakia where less than 4% of the CHC burden is attributable to migrants from endemic countries. The differences in general population prevalence between EU/EEA countries, together with the contribution of migrants moving within the EU/EEA from high to low prevalence countries, suggests that there may also be value in allocating EU health funding to scale up and systematise screening and treatment efforts in EU/EEA countries with a high general population prevalence, to strengthen efforts to reduce cross-border health threats and to improve overall population health in the EU. A recent modelling study estimated that only around a third of all CHC cases across Europe have been diagnosed and that there are wide differences in both the proportion diagnosed and the proportion on treatment comparing EU/EEA countries [6]. There is however no data on the estimated proportion of migrants from endemic countries that are diagnosed. The data reported, as well as the strategies and interventions suggested, in this study can contribute to increasing the proportion of cases of CHC diagnosed and on treatment.

Previous studies of hepatitis B/C screening implemented among migrant populations describe different models. These include: outreach offering awareness raising and/or on-site screening by public health teams in social, civic or cultural locations familiar to the target community [29–31]; invitation-based screening where municipal, population or patient registries are used to send postal invitations to attend screening to people born in higher prevalence countries [32, 33]; opportunistic offering of screening to patients with country of birth-related risk factors who attend health care services for other health issues [34, 35]; and adding viral hepatitis screening to an existing screening programme, such as for tuberculosis, that targets people from high prevalence countries [36]. Each of these models differs in terms of logistical and resource requirements, uptake and case yield, but few studies have compared the characteristics and effectiveness of different models [37]. The characteristics of screening programmes that have demonstrated success in uptake and yield include: involvement of the community in planning and raising awareness; provision of screening in suitable and accessible locations for the target community; provision of language support, for example, translated materials and interpreters; planning and provision for people without health insurance coverage; cultural sensitivity about and efforts to reduce or eliminate stigma; and availability of follow-up and care in the community. Retention within a follow-up care and treatment pathway is crucial to ensure that the public health benefits of screening are realised [38]. A summary of migrant-specific screening programmes, an appraisal of factors contributing to success, and a range of other scientific and practical resources are available as part of the HEPscreen Toolkit, produced by the EU-funded HEPscreen project, which focused on screening for chronic viral hepatitis among migrants [39].

In four countries, (Cyprus, Lichtenstein, Luxembourg and the Netherlands) the upper range of the estimated relative contribution of migrants as a proportion of the total number of estimated cases was found to be over 100%. This reflects unmeasured correlation between the input parameters (prevalence in countries of birth and the size of the migrant population) for the Delta method as well as the strong skew in the distribution of cases in the general population in these countries. It is also possible that the prevalence in the general population in these EU/EEA countries is under-estimated, due to unrepresentative sampling or low participation of risk populations who are harder to reach, such as PWID and ethnic minorities as well as migrants. Under-representation of migrants and ethnic minorities is a widely recognised phenomenon in clinical trial and health survey research, [40, 41] although literature specifically focused on sero-prevalence sampling and uptake is very limited [23]. In addition, it is possible that estimates based on prevalence in countries of birth results in over-estimation of the prevalence among migrants. For example, it is probable that no longer living in a high prevalence country would reduce the risk of transmission of HCV, especially since much of the transmission in higher prevalence countries is nosocomial [42] and most EU/EEA countries have successfully controlled nosocomial transmission through health care infection control procedures. Over-estimation may also be due to the characteristics of migrants to the EU/EEA, who may be younger and healthier, and so less likely to have experienced hospitalisation or serious medical intervention and more likely to have benefited from improved primary prevention in the last two decades. Migrants to the EU may also be from higher socio-economic groups in their countries of birth and able to afford better, safer health care [43]. We sought to test out this theory of over-estimation and found that, for just three of the ten countries of birth for which we retrieved estimates for, [15, 29, 30, 44–47] the prevalence in migrants was lower than the reported in-country prevalence.

Despite the systematic nature of data retrieval, there are some data limitations. Detailed demographic data was available from Eurostat for only 21 EU/EEA countries. There is also heterogeneity in the parameters and methods used by the different demographic databases and in the way that demographic data on migration is collected and reported by EU/EEA countries. From other literature, we know that countries such as Germany, France and the Netherlands require municipal registration upon arrival in a new area and collect country of birth data as part of registration. Other countries, like the UK, rely on population census data to systematically collect population size and origin [48]. However, there is very limited information in the literature on the methods used by each database or on the heterogeneity of demographic data collection methods across the EU/EEA. Despite these differences, the demographic data used in this study are derived from databases that in turn derive data from national statistical institutes. We believe it to be the best and most reputable available. These analyses do not include undocumented migrants, partly because the size and origin of this population is very dynamic, but mostly because of the imprecision and unreliability of the data [49]. The use of systematic reviews and meta-analyses for the country of birth prevalence input has reduced the reliance on less reliable single study estimates, and whilst we critically appraised the quality of the systematic reviews and meta-analyses, which all applied quality criteria to select studies, we did not directly assess the quality of the estimates included in these reviews. A further note of caution relates to the studies retrieved on prevalence in migrants, as just ten countries of birth were represented in the estimates and few studies had the specific aim of estimating the prevalence in migrants. The use of convenience sampling in many of these studies increases the chance of selection bias and, specifically, that people already diagnosed may not present for screening.

## Conclusions

Advances in antiviral treatment open up the possibility of eliminating hepatitis C infection in Europe, but achieving this will require countries to scale up and better target screening, linkage to care and treatment. This study provides strategic, timely and detailed epidemiological intelligence for EU/EEA countries on the hepatitis C burden among migrant populations, a key population group affected by this infection in Europe. It also provides prevalence estimates for 224 countries and territories, which should serve as a useful resource for other countries and regions wishing to understand the relative contribution of migrants to the burden of hepatitis C. This intelligence, together with the learning from previous migrant-specific screening projects in the EU/EEA [39], can help to inform the design of screening programmes to reach migrant populations most affected by chronic hepatitis C infection. A targeted approach in higher risk populations makes more effective use of health care resources and contributes to reducing health inequalities. The World Health Organisation’s Global Strategy for Viral Hepatitis [50] and the European Action Plan [51] both share the ambitious goal of elimination of viral hepatitis by 2030. If this goal is to be realised, it will be essential to dramatically increase the proportion of people who are diagnosed, aware of their infection and on treatment. Future research can contribute by focussing on improving the evidence base on effective strategies to reach and retain migrant and other risk populations in screening and treatment and on cost-effectiveness across the treatment cascade.

## Additional file

Additional file 1: Annex 1. Search strategy for systematic reviews and meta-analyses of global/worldwide prevalence studies; **Annex 2.** PRISMA diagram of systematic search for global HBsAg/anti-HCV sero-prevalence; **Annex 3.** Description of the search strategy and retrievals for chronic hepatitis B/C prevalence studies among migrants; **Annex 4.** PRISMA flow diagram of systematic search for HBsAg/anti-HCV sero-prevalence in migrants in the EU/EEA; **Annex 5.** Inclusion/exclusion criteria for systematic reviews/meta analyses reporting the prevalence worldwide/in various continents/regions; **Annex 6.** Inclusion/exclusion criteria for studies retrieved by the search for articles reporting the prevalence among migrants; **Annex 7.** Comparison of anti-HCV estimates from SRs/MAs and rationale for estimate selected for analysis; **Annex 8.** Anti-HCV prevalence estimates selected from systematic reviews; **Annex 9.** Chronic Hepatitis C burden country tables: 50 largest migrant populations per EU/EEA country; **Annex 10.** Countries of birth of foreign-born migrants found amongst the ten migrant groups most affected by chronic hepatitis C in 10 or more of the 31 EU/EEA countries. (DOCX 308 kb)

## Abbreviations

CHC: Chronic hepatitis c
CIs: Confidence Intervals
DAAs: Direct acting antivirals
ECDC: European centre for disease prevention and control
EU/EEA: European Union/European Economic Area
GBD: Global burden of disease
HCC: Hepatocellular carcinoma
HCV: Hepatitis C virus
OECD: Organisation for economic co-operation and development
PRISMA: Preferred reporting items for systematic reviews and meta analyses
PWID: People who inject drugs
UK: United Kingdom
WHO: World Health Organisation
