# Supplementary material for: Estimating the scale of chronic hepatitis C virus infection in the EU/EEA: a focus on migrants from anti-HCV endemic countries
Source: BMC Infect Dis. 2018 Jan 16;18:42. doi: 10.1186/s12879-017-2908-5 (PMC5771208; doi:10.1186/s12879-017-2908-5)
Supplement: Additional file 1: Annex 1. — Search strategy for systematic reviews and meta-analyses of global/worldwide prevalence studies; Annex 2. PRISMA diagram of systematic search for global HBsAg/anti-HCV sero-prevalence; Annex 3. Description of the search strategy and retrievals for chronic hepatitis B/C prevalence studies among migrants; Annex 4. PRISMA flow diagram of systematic search for HBsAg/anti-HCV sero-prevalence in migrants in the EU/EEA; Annex 5. Inclusion/exclusion criteria for systematic reviews/meta analyses reporting the prevalence worldwide/in various continents/regions; Annex 6. Inclusion/exclusion criteria for studies retrieved by the search for articles reporting the prevalence among migrants; Annex 7. Comparison of anti-HCV estimates from SRs/MAs and rationale for estimate selected for analysis; Annex 8. Anti-HCV prevalence estimates selected from systematic reviews; Annex 9. Chronic Hepatitis C burden country tables: 50 largest migrant populations per EU/EEA country; Annex 10. Countries of birth of foreign-born migrants found amongst the ten migrant groups most affected by chronic hepatitis C in 10 or more of the 31 EU/EEA countries. (DOCX 308 kb) [file 12879_2017_2908_MOESM1_ESM.docx]

**Additional file 1**

**Estimating the scale of chronic hepatitis C virus infection among migrants in EU/EEA countries: Supplementary Data**

Authors: A.M. Falla (corresponding author)^1,2 *^, A.A. Ahmad^3*^, E. Duffell^4^, T. Noori^4^, I.K. Veldhuijzen^2,5^

^1^ Department of Public Health, Erasmus MC, University Medical Center Rotterdam, the Netherlands

^2^ Division of Infectious Disease Control, Municipal Public Health Service Rotterdam-Rijnmond, the Netherlands

^3^  Dept. of Health Sciences, Hamburg University of Applied Sciences, Faculty Life Sciences / Public Health Research, Hamburg, Germany.

^4^European Centre for Disease Control and Prevention (ECDC), Stockholm, Sweden

^5^ Center for Infectious Disease Control, National Institute for Public Health and the Environment, Bilthoven, the Netherlands

* Both authors contributed equally to the manuscript

Corresponding author contact details: [am.falla@rotterdam.nl](mailto:am.falla@rotterdam.nl); [abby.falla@gmail.com](mailto:abby.falla@gmail.com); Tel: +31(0)10 4339267, Fax: + 31(0)10 4339747. PO Box 70032, 3000 LP Rotterdam, The Netherlands.

Table of Contents

[Annex 1: Search strategy for systematic reviews and meta-analyses of global/worldwide prevalence studies 4](#_Toc477511574)

[Annex 2: PRISMA diagram of systematic search for global HBsAg/anti-HCV sero-prevalence 7](#_Toc477511575)

[Annex 3: Description of the search strategy and retrievals for chronic hepatitis B/C prevalence studies among migrants 8](#_Toc477511576)

[Annex 4: PRISMA flow diagram of systematic search for HBsAg/anti-HCV sero-prevalence in migrants in the EU/EEA 12](#_Toc477511577)

[Annex 5: Inclusion/exclusion criteria for systematic reviews/meta analyses reporting the prevalence worldwide/in various continents/regions 13](#_Toc477511578)

[Annex 6: Inclusion/exclusion criteria for studies retrieved by the search for articles reporting the prevalence among migrants 14](#_Toc477511579)

[Annex 7: Comparison of anti-HCV estimates from SRs/MAs and rationale for estimate selected for analysis 15](#_Toc477511580)

[Annex 8: Anti-HCV prevalence estimates selected from systematic reviews 19](#_Toc477511581)

[Annex 9: Chronic Hepatitis C burden country tables: 50 largest migrant populations per EU/EEA country 27](#_Toc477511582)

[9.1 Austria 28](#_Toc477511583)

[9.2 Belgium 29](#_Toc477511584)

[9.3 Bulgaria 30](#_Toc477511585)

[9.4 Croatia 31](#_Toc477511586)

[9.5 Cyprus 32](#_Toc477511587)

[9.6 Czech Republic 33](#_Toc477511588)

[9.7 Denmark 34](#_Toc477511589)

[9.8 Estonia 35](#_Toc477511590)

[9.9 Finland 36](#_Toc477511591)

[9.10 France 37](#_Toc477511592)

[9.11 Germany 38](#_Toc477511593)

[9.12 Greece 39](#_Toc477511594)

[9.13 Hungary 40](#_Toc477511595)

[9.14 Iceland 41](#_Toc477511596)

[9.15 Republic of Ireland 42](#_Toc477511597)

[9.16 Italy 43](#_Toc477511598)

[9.17 Latvia 44](#_Toc477511599)

[9.18 Liechtenstein 45](#_Toc477511600)

[9.19 Lithuania 46](#_Toc477511601)

[9.20 Luxembourg 47](#_Toc477511602)

[9.21 Malta 48](#_Toc477511603)

[9.22 The Netherlands 49](#_Toc477511604)

[9.23 Norway 50](#_Toc477511605)

[9.24 Poland 51](#_Toc477511606)

[9.25 Portugal 52](#_Toc477511607)

[9.26 Romania 53](#_Toc477511608)

[9.27 Slovakia 54](#_Toc477511609)

[9.28 Slovenia 55](#_Toc477511610)

[9.29 Spain 56](#_Toc477511611)

[9.30 Sweden 57](#_Toc477511612)

[9.31 United Kingdom 58](#_Toc477511613)

[Annex 10 Countries of birth of foreign-born migrants found amongst the ten migrant groups most affected by chronic hepatitis C in 10 or more of the 31 EU/EEA countries 59](#_Toc477511614)

# Annex 1: Search strategy for systematic reviews and meta-analyses of global/worldwide prevalence studies

The search was structured to include terms from four topic areas, i.e. the infection, the outcome, the population and the study design. Separate search terms are included, as well as controlled vocabulary search terms (EMTree, comparable to MeSH terms in Medline). The search terms included per topic area in the EMBASE search are described below. The full search strategy for systematic reviews in each database is also presented below.

The infection: hepatitis B or C
in abstract or title:
hepatitis B, hepatitis B virus, hepatitis C, hepatitis C virus [EMtree]
hbv, hcv

The outcome: prevalence
in abstract or title:
prevalence, seroprevalence;
serology [EMTree]; prevalen*, seroprevalen*, serolog*, marker*, seroepidemiol*
hepatitis rapid test, hepatitis B rapid test, hepatitis C rapid test [EMTree]
hepatitis B antibody [EMTree]
hepatitis B antigen, hepatitis B surface antigen, HBsAg, HBs-Ag, HB-s-Ag
hepatitis C antigen [EMTree]
hepatitis C antibody [EMTree]

The population: general population worldwide/global:
in abstract or title:
Health survey [EMTree]; 'residual sera', survey*, surveillan*,
Population, Population group, Population research [EMTree]; population*, communit*,
Geographic names, Geographic distribution [EMTree]; geograph*, worldwide, world-wide, global, europe*, asia*, america*, africa*, australia*, countr*
Population and population related phenomena [EMTree];

Study design
in abstract or title:
Systematic review [EMTree]; ‘systematic* AND review*’,
comprehensiv*, exhaustiv* NEAR/3 literature*
Meta analysis [EMTree]; meta NEXT/1 analy*

**Full search strategy for reviews, systematic review and meta analyses on hepatitis B and hepatitis C prevalence worldwide at country level (run by Erasmus MC Medical Library on 21 January 2015)**

**Embase.com 87**((('hepatitis B'/exp OR 'Hepatitis B virus'/exp OR 'hepatitis C'/exp OR 'Hepatitis C virus'/exp OR ((hepatitis NEXT/3 (B OR C)) OR hbv OR hcv):ab,ti) AND (prevalence/de OR seroprevalence/de OR serology/exp OR epidemiology/exp OR (seroprevalen* OR prevalen* OR serolog* OR seroepidemiol* OR epidemiolog*):ab,ti)) OR ('hepatitis rapid test'/exp OR 'hepatitis B rapid test'/exp OR 'hepatitis C rapid test'/exp OR 'hepatitis B antigen'/de OR 'hepatitis C antigen'/exp OR 'hepatitis C antibody'/exp OR 'hepatitis B antibody'/exp OR 'hepatitis B surface antigen'/de OR (HBsAg OR HBs-Ag OR HB-s-Ag OR (('Hepatitis b' OR 'Hepatitis c' OR hbv OR hcv) NEAR/3 ( 'surface antigen' OR 'surface antigens' OR antibod*))):ab,ti)) AND ('health survey'/de OR population/de OR 'population group'/exp OR 'geographic names'/exp OR 'population research'/exp OR 'geographic distribution'/exp OR 'population and population related phenomena'/exp OR (population* OR communit* OR 'residual sera' OR survey* OR surveillan* OR geograph* OR worldwide OR world-wide OR global OR europe* OR asia* OR america* OR africa* OR australia* OR countr*):ab,ti) AND ('systematic review'/exp OR 'meta analysis'/exp OR ((systematic* AND review*) OR ((comprehensiv* OR exhaustiv*) NEAR/3 literature*) OR (meta NEXT/1 analy*)):ab,ti)

**Medline (OvidSP) 326**(((exp hepatitis B/ OR Hepatitis B virus/ OR hepatitis C/ OR Hepacivirus/ OR ((hepatitis ADJ3 (B OR C)) OR hbv OR hcv).ab,ti.) AND (prevalence/ OR Seroepidemiologic Studies/ OR serology/ OR Serologic Tests/ OR (seroprevalen* OR prevalen* OR serolog* OR seroepidemiol* OR epidemiolog*).ab,ti.)) OR (exp hepatitis B antigen/ OR hepatitis C antigen/ OR hepatitis C antibodies/ OR hepatitis B antibodies/ OR (HBsAg OR HBs-Ag OR HB-s-Ag OR ((Hepatitis b OR Hepatitis c OR hbv OR hcv) ADJ3 ( surface antigen OR surface antigens OR antibod*))).ab,ti.)) AND (exp Health Surveys/ OR exp Population Groups/ OR exp Population/ OR exp Geographic Locations/ OR (population* OR communit* OR residual sera OR survey* OR surveillan* OR geograph* OR worldwide OR world-wide OR global OR europe* OR asia* OR america* OR africa* OR australia* OR countr*).ab,ti.) AND (Meta-Analysis.pt. OR ((systematic* AND review*) OR ((comprehensiv* OR exhaustiv*) ADJ3 literature*) OR (meta ADJ analy*)).ab,ti.)

**Cochrane DARE 0**(((((hepatitis NEXT/3 (B OR C)) OR hbv OR hcv):ab,ti) AND ((seroprevalen* OR prevalen* OR serolog* OR seroepidemiol* OR epidemiolog*):ab,ti)) OR ((HBsAg OR HBs-Ag OR HB-s-Ag OR (('Hepatitis b' OR 'Hepatitis c' OR hbv OR hcv) NEAR/3 ( 'surface antigen' OR 'surface antigens' OR antibod*))):ab,ti)) AND ((population* OR communit* OR 'residual sera' OR survey* OR surveillan* OR geograph* OR worldwide OR world-wide OR global OR europe* OR asia* OR america* OR africa* OR australia* OR countr*):ab,ti)

**Web-of-science 366**TS=((((((hepatitis NEAR/3 (B OR C)) OR hbv OR hcv)) AND ((seroprevalen* OR prevalen* OR serolog* OR seroepidemiol* OR epidemiolog*))) OR ((HBsAg OR HBs-Ag OR HB-s-Ag OR (("Hepatitis b" OR "Hepatitis c" OR hbv OR hcv) NEAR/3 ( "surface antigen" OR "surface antigens" OR antibod*))))) AND ((population* OR communit* OR "residual sera" OR survey* OR surveillan* OR geograph* OR worldwide OR world-wide OR global OR europe* OR asia* OR america* OR africa* OR australia* OR countr*)) AND (((systematic* AND review*) OR ((comprehensiv* OR exhaustiv*) NEAR/3 literature*) OR (meta NEAR/1 analy*))))

**PubMed publisher 8**(((hepatitis B[mh] OR Hepatitis B virus[mh] OR hepatitis C[mh] OR Hepacivirus[mh] OR ((hepatitis AND (B OR C)) OR hbv OR hcv)) AND (prevalence[mh] OR Seroepidemiologic Studies[mh] OR serology[mh] OR Serologic Tests[mh] OR (seroprevalen*[tiab] OR prevalen*[tiab] OR serolog*[tiab] OR seroepidemiol*[tiab] OR epidemiolog*[tiab]))) OR (hepatitis B antigen[mh] OR hepatitis C antigen[mh] OR hepatitis C antibodies[mh] OR hepatitis B antibodies[mh] OR (HBsAg OR HBs-Ag OR HB-s-Ag OR ((Hepatitis b OR Hepatitis c OR hbv OR hcv) AND ( surface antigen OR surface antigens OR antibod*[tiab]))))) AND (Health Surveys[mh] OR Population Groups[mh] OR Population[mh] OR Geographic Locations[mh] OR (population*[tiab] OR communit*[tiab] OR residual sera OR survey*[tiab] OR surveillan*[tiab] OR geograph*[tiab] OR worldwide OR world-wide OR global OR europe*[tiab] OR asia*[tiab] OR america*[tiab] OR africa*[tiab] OR australia*[tiab] OR countr*[tiab])) AND (Meta-Analysis[pt] OR ((systematic*[tiab] AND review*[tiab]) OR ((comprehensiv*[tiab] OR exhaustiv*[tiab]) AND literature*[tiab]) OR (meta analy*[tiab]))) AND publisher

**Google Scholar**"hepatitis B|C"|hbv|hcv seroprevalence|prevalence|serology|seroepidemioly|epidemiology population|community|geography|worldwide|"world wide"|global|europe|asia|america|africa|australia|country|countries "systematic review|"meta analysis"

# Annex 2: PRISMA diagram of systematic search for global HBsAg/anti-HCV sero-prevalence

Records identified through database searching n = 1772

Records after duplicates removed
n = 1182

Records screened
n = 802

Records excluded based on title/abstract n=757

Full-text articles assessed for eligibility
n = 45

Full-text articles excluded, with reasons n = 27

Studies included in qualitative synthesis

N = 18

Records after exclusion of articles published before 2009 n = 802

| **Full Text Exclusion: Reason** | **N** |
| --- | --- |
| No full text available | 4 |
| No prevalence estimate | 5 |
| Conference abstract (full paper available or insufficient detail to assess the methodological quality | 6 |
| Unrepresentative for or no data on the general population | 5 |
| Not a systematic review/meta analysis | 3 |
| Poor/limited search | 2 |
| Methodologically vague | 1 |
| Protocol only | 1 |
| Total | 27 |

# Annex 3: Description of the search strategy and retrievals for chronic hepatitis B/C prevalence studies among migrants

The search was structured to include terms from four topic areas, i.e. the infection, the outcome, the population and geographical area. Separate search terms are included, as well as controlled vocabulary search terms (EMTree, comparable to MeSH terms in Medline). The search terms included per topic area in the EMBASE search are described below. The full search strategy for migrant studies in each database is also presented below.

The infection: hepatitis B or C
in abstract or title:
hepatitis B, Hepatitis B virus, hepatitis C, hepatitis C virus [EMtree]
hbv, hcv

The outcome: prevalence
in abstract or title:
prevalence, seroprevalence;
serology [EMTree]; prevalen*, seroprevalen*, serolog*, marker*, seroepidemiol*
hepatitis rapid test, hepatitis B rapid test, hepatitis C rapid test [EMTree]
hepatitis B antibody [EMTree]
hepatitis B antigen, hepatitis B surface antigen, HBsAg, HBs-Ag, HB-s-Ag
hepatitis C antigen [EMTree]
hepatitis C antibody [EMTree]

The population: migrants:
in abstract or title:
migrant, migration, minority group, ethnic group, ethnicity [EMTree]
migrant*, emigrant*, immigrant*, migrat*, emigrat*, immigrat*, asylum seeker*, refugee*, minorit*, ethnic*
(countr*, africa*, asia*, racial*, eastern europe, endemic areas, endemic area, foreign*) NEAR (origin*, born*, nationalit*)

Geographical area: EU/EEA countries
in abstract or title or journal title or country of journal or country of author or author address:
Europe, European [EMTree]
European Union, europ*, eu
Austria*, Belgium, belgian, Bulgaria*, Croatia*, Cyprus, cypriot*, Czech*, Denmark, danish, Estonia*, Finland, finnish, finn, France, french*, German*, Greece, greek, Hungar*, Ireland, irish, Italy, italian*, Latvia*, Lithuania*, Luxemburg*, Malta, maltese, Netherlands, dutch, Poland, polish*, Portug*, Romania*, Slovak*, Slovenia*, Spain, spanish, spaniard*, Sweden, swedish, "united kingdom", "great britain", uk, british*, England, english, Scotland, scottish, Wales, welsh, Norway, norweg*, Iceland*

**Full search strategy: Hepatitis B and C among migrants in the EU (run by Erasmus MC Medical Library on 17 November 2014)**

**Embase.com 1210 (1204 unique records)**((('hepatitis B'/exp OR 'Hepatitis B virus'/exp OR 'hepatitis C'/exp OR 'Hepatitis C virus'/exp OR ((hepatitis NEAR/3 (B OR C)) OR hbv OR hcv):ab,ti) AND (prevalence/de OR seroprevalence/de OR serology/exp OR (seroprevalen* OR prevalen* OR marker* OR serolog* OR seroepidemiol*):ab,ti)) OR ('hepatitis rapid test'/exp OR 'hepatitis B rapid test'/exp OR 'hepatitis C rapid test'/exp OR 'hepatitis B antigen'/de OR 'hepatitis C antigen'/exp OR 'hepatitis C antibody'/exp OR 'hepatitis B antibody'/exp OR 'hepatitis B surface antigen'/de OR (HBsAg OR HBs-Ag OR HB-s-Ag OR (('Hepatitis b' OR 'Hepatitis c' OR hbv OR hcv) NEAR/3 ( 'surface antigen' OR 'surface antigens' OR antibod*))):ab,ti)) AND (migrant/exp OR migration/exp OR 'minority group'/exp OR 'ethnic group'/exp OR ethnicity/exp OR (migrant* OR emigrant* OR immigrant* OR migrat* OR emigrat* OR immigrat* OR (asylum NEXT/1 seeker*) OR refugee* OR minorit* OR ethnic* OR ((countr* OR africa* OR asia* OR racial* OR 'eastern europe' OR 'endemic areas' OR 'endemic area' OR foreign* ) NEAR/3 (origin* OR born* OR nationalit*))):ab,ti) AND (europe/exp OR European/exp OR 'European Union'/de OR (europ* OR eu OR iceland* OR norway OR norweg* OR sweden OR swedish OR finland OR finnish OR finn OR denmark OR danish OR "great britain" OR "united kingdom" OR uk OR british* OR England OR english OR Scotland OR scottish OR Wales OR welsh OR Ireland OR irish OR Netherlands OR dutch OR Belgium OR belgian OR France OR french* OR Luxemburg* OR Spain OR spanish OR spaniard* OR Portug* OR Italy OR italian* OR Switzerland OR swiss* OR Austria* OR German* OR Poland OR polish* OR Hungar* OR Czech* OR Croatia* OR Slovak* OR Slovenia* OR Romania* OR Bulgaria* OR Lithuania* OR Latvia* OR Estonia* OR Greece OR greek OR Turkey OR turkish OR Macedonia* OR Cyprus OR cypriot* OR Malta OR maltese):ab,ti,ca,ta,cy,ad) AND [english]/lim NOT ([Conference Abstract]/lim OR [Letter]/lim OR [Note]/lim OR [Conference Paper]/lim OR [Editorial]/lim)

**Medline (OvidSP) 989 (191 unique records)**(((exp hepatitis B/ OR Hepatitis B virus/ OR hepatitis C/ OR Hepacivirus/ OR ((hepatitis ADJ3 (B OR C)) OR hbv OR hcv).ab,ti.) AND (prevalence/ OR Seroepidemiologic Studies/ OR serology/ OR Serologic Tests/ OR (seroprevalen* OR prevalen* OR marker* OR serolog* OR seroepidemiol*).ab,ti.)) OR (exp hepatitis B antigen/ OR hepatitis C antigen/ OR hepatitis C antibodies/ OR hepatitis B antibodies/ OR (HBsAg OR HBs-Ag OR HB-s-Ag OR ((Hepatitis b OR Hepatitis c OR hbv OR hcv) ADJ3 (surface antigen* OR antibod*))).ab,ti.)) AND (Transients and Migrants/ OR Human Migration/ OR minority group/ OR ethnic group/ OR Emigrants and Immigrants/ OR Emigration and Immigration/ OR (migrant* OR emigrant* OR immigrant* OR migrat* OR emigrat* OR immigrat* OR asylum seeker* OR refugee* OR minorit* OR ethnic* OR ((countr* OR africa* OR asia* OR racial* OR eastern europe OR endemic areas OR endemic area OR foreign* ) ADJ3 (origin* OR born* OR nationalit*))).ab,ti.) AND (exp europe/ OR (europ* OR eu OR iceland* OR norway OR norweg* OR sweden OR swedish OR finland OR finnish OR finn OR denmark OR danish OR "great britain" OR "united kingdom" OR uk OR british* OR England OR english OR Scotland OR scottish OR Wales OR welsh OR Ireland OR irish OR Netherlands OR dutch OR Belgium OR belgian OR France OR french* OR Luxemburg* OR Spain OR spanish OR spaniard* OR Portug* OR Italy OR italian* OR Switzerland OR swiss* OR Austria* OR German* OR Poland OR polish* OR Hungar* OR Czech* OR Croatia* OR Slovak* OR Slovenia* OR Romania* OR Bulgaria* OR Lithuania* OR Latvia* OR Estonia* OR Greece OR greek OR Turkey OR turkish OR Macedonia* OR Cyprus OR cypriot* OR Malta OR maltese).ab,ti,jn,cp,in.) AND english.la. NOT (letter OR news OR comment OR editorial OR congresses OR abstracts).pt.

**Cochrane 8 (3 unique records)**(((((hepatitis NEAR/3 (B OR C)) OR hbv OR hcv):ab,ti) AND ((seroprevalen* OR prevalen* OR marker* OR serolog* OR seroepidemiol*):ab,ti)) OR ((HBsAg OR HBs-Ag OR HB-s-Ag OR (('Hepatitis b' OR 'Hepatitis c' OR hbv OR hcv) NEAR/3 ( 'surface antigen' OR 'surface antigens' OR antibod*))):ab,ti)) AND ((migrant* OR emigrant* OR immigrant* OR migrat* OR emigrat* OR immigrat* OR (asylum NEXT/1 seeker*) OR refugee* OR minorit* OR ethnic* OR ((countr* OR africa* OR asia* OR racial* OR 'eastern europe' OR 'endemic areas' OR 'endemic area' OR foreign* ) NEAR/3 (origin* OR born* OR nationalit*))):ab,ti) AND ((europ* OR eu OR iceland* OR norway OR norweg* OR sweden OR swedish OR finland OR finnish OR finn OR denmark OR danish OR "great britain" OR "united kingdom" OR uk OR british* OR England OR english OR Scotland OR scottish OR Wales OR welsh OR Ireland OR irish OR Netherlands OR dutch OR Belgium OR belgian OR France OR french* OR Luxemburg* OR Spain OR spanish OR spaniard* OR Portug* OR Italy OR italian* OR Switzerland OR swiss* OR Austria* OR German* OR Poland OR polish* OR Hungar* OR Czech* OR Croatia* OR Slovak* OR Slovenia* OR Romania* OR Bulgaria* OR Lithuania* OR Latvia* OR Estonia* OR Greece OR greek OR Turkey OR turkish OR Macedonia* OR Cyprus OR cypriot* OR Malta OR maltese):ab,ti)

**Web-of-science 547 (170 unique records)**TS=((((((hepatitis NEAR/3 (B OR C)) OR hbv OR hcv)) AND ((seroprevalen* OR prevalen* OR marker* OR serolog* OR seroepidemiol*))) OR ((HBsAg OR HBs-Ag OR HB-s-Ag OR (("Hepatitis b" OR "Hepatitis c" OR hbv OR hcv) NEAR/3 ( "surface antigen" OR "surface antigens" OR antibod*))))) AND ((migrant* OR emigrant* OR immigrant* OR migrat* OR emigrat* OR immigrat* OR (asylum NEAR/1 seeker*) OR refugee* OR minorit* OR ethnic* OR ((countr* OR africa* OR asia* OR racial* OR "eastern europe" OR "endemic areas" OR "endemic area" OR foreign* ) NEAR/3 (origin* OR born* OR nationalit*)))) AND ((europ* OR eu OR iceland* OR norway OR norweg* OR sweden OR swedish OR finland OR finnish OR finn OR denmark OR danish OR "great britain" OR "united kingdom" OR uk OR british* OR England OR english OR Scotland OR scottish OR Wales OR welsh OR Ireland OR irish OR Netherlands OR dutch OR Belgium OR belgian OR France OR french* OR Luxemburg* OR Spain OR spanish OR spaniard* OR Portug* OR Italy OR italian* OR Switzerland OR swiss* OR Austria* OR German* OR Poland OR polish* OR Hungar* OR Czech* OR Croatia* OR Slovak* OR Slovenia* OR Romania* OR Bulgaria* OR Lithuania* OR Latvia* OR Estonia* OR Greece OR greek OR Turkey OR turkish OR Macedonia* OR Cyprus OR cypriot* OR Malta OR maltese)) )

**Scopus 1012 (304 unique records)**TITLE-ABS-KEY((((((hepatitis W/3 (B OR C)) OR hbv OR hcv)) AND ((seroprevalen* OR prevalen* OR marker* OR serolog* OR seroepidemiol*))) OR ((HBsAg OR HBs-Ag OR HB-s-Ag OR (("Hepatitis b" OR "Hepatitis c" OR hbv OR hcv) W/3 ( "surface antigen" OR "surface antigens" OR antibod*))))) AND ((migrant* OR emigrant* OR immigrant* OR migrat* OR emigrat* OR immigrat* OR (asylum W/1 seeker*) OR refugee* OR minorit* OR ethnic* OR ((countr* OR africa* OR asia* OR racial* OR "eastern europe" OR "endemic areas" OR "endemic area" OR foreign* ) W/3 (origin* OR born* OR nationalit*)))) AND ((europ* OR eu OR iceland* OR norway OR norweg* OR sweden OR swedish OR finland OR finnish OR finn OR denmark OR danish OR "great britain" OR "united kingdom" OR uk OR british* OR England OR english OR Scotland OR scottish OR Wales OR welsh OR Ireland OR irish OR Netherlands OR dutch OR Belgium OR belgian OR France OR french* OR Luxemburg* OR Spain OR spanish OR spaniard* OR Portug* OR Italy OR italian* OR Switzerland OR swiss* OR Austria* OR German* OR Poland OR polish* OR Hungar* OR Czech* OR Croatia* OR Slovak* OR Slovenia* OR Romania* OR Bulgaria* OR Lithuania* OR Latvia* OR Estonia* OR Greece OR greek OR Turkey OR turkish OR Macedonia* OR Cyprus OR cypriot* OR Malta OR maltese)) )

**PubMed publisher 37 (27 unique records)**(((hepatitis B*[tiab] OR hepatitis C*[tiab] OR hbv[tiab] OR hcv[tiab]) AND ((seroprevalen*[tiab] OR prevalen*[tiab] OR marker*[tiab] OR serolog*[tiab] OR seroepidemiol*[tiab] OR surface antigen*[tiab] OR antibod*[tiab]))) OR ((HBsAg[tiab] OR HBs-Ag[tiab] OR HB-s-Ag[tiab] ))) AND ((migrant*[tiab] OR emigrant*[tiab] OR immigrant*[tiab] OR migrat*[tiab] OR emigrat*[tiab] OR immigrat*[tiab] OR asylum seeker*[tiab] OR refugee*[tiab] OR minorit*[tiab] OR ethnic*[tiab] OR ((countr*[tiab] OR africa*[tiab] OR asia*[tiab] OR racial*[tiab] OR eastern europe*[tiab] OR endemic area*[tiab] OR foreign*[tiab] ) AND (origin*[tiab] OR born*[tiab] OR nationalit*[tiab])))) AND (europ* OR eu OR iceland* OR norway OR norweg* OR sweden OR swedish OR finland OR finnish OR finn OR denmark OR danish OR "great britain" OR "united kingdom" OR uk OR british* OR England OR english OR Scotland OR scottish OR Wales OR welsh OR Ireland OR irish OR Netherlands OR dutch OR Belgium OR belgian OR France OR french* OR Luxemburg* OR Spain OR spanish OR spaniard* OR Portug* OR Italy OR italian* OR Switzerland OR swiss* OR Austria* OR German* OR Poland OR polish* OR Hungar* OR Czech* OR Croatia* OR Slovak* OR Slovenia* OR Romania* OR Bulgaria* OR Lithuania* OR Latvia* OR Estonia* OR Greece OR greek OR Turkey OR turkish OR Macedonia* OR Cyprus OR cypriot* OR Malta OR maltese) AND english[la] AND publisher

**Google Scholar 200 (155 unique records)**"hepatitis B|C" prevalence|seroprevalence|antigen|antibody|antigens|antibodies
migrant|migrants|minority|minorities|ethnic|ethnicity|immigrants|immigrant|foreigners europe|European

# Annex 4: PRISMA flow diagram of systematic search for HBsAg/anti-HCV sero-prevalence in migrants in the EU/EEA

Records excluded based on title/abstract

n = 1365

Records retrieved via systematic search
n = 4003

Records after duplicates removed
n = 2054

Records after exclusion of articles published before 2000

n = 1443

Records screened
n = 1443

Full-text articles assessed for eligibility

n = 78

Records excluded based on full-text (reasons documented)

n =22

Studies included in qualitative synthesis

n = 56

| Reason for exclusion | N |
| --- | --- |
| Not in English | 14 |
| Commentary only | 1 |
| Modelled data only | 1 |
| Unspecified virological markers | 1 |
| No data on migrants | 4 |
| Unreliable/unknown denominator | 1 |
| Total | 22 |

# Annex 5: Inclusion/exclusion criteria for systematic reviews/meta analyses reporting the prevalence worldwide/in various continents/regions

Studies retrieved by the search for systematic reviews/meta analyses reporting the prevalence worldwide/in various continents/regions will be assessed by the following criteria:

Inclusion criteria:

- Studies must be of a systematic review and/or meta analytic methodological design
- Studies must include primary research reporting anti-HCV (and/or HCV RNA) prevalence in humans measured via (laboratory) testing of samples of bodily fluid(s)

Exclude:

- Guidelines, opinion pieces or commentaries that are not systematic reviews/meta analyses of prevalence estimates
- Self-reported anti-HCV status
- Studies with unspecified virological markers of hepatitis C infection
- Modelling studies with no original data
- Environmental studies, technology assessments (studies on diagnostic and/or laboratory methods)

# Annex 6: Inclusion/exclusion criteria for studies retrieved by the search for articles reporting the prevalence among migrants

Studies retrieved by the search for articles reporting the prevalence among migrants will be assessed by the following criteria:

Inclusion criteria:

- Studies must suggest/report HBsAg/anti-HCV (and/or HBV DNA/HCV RNA) prevalence in humans measured via (laboratory) testing of samples of bodily fluid(s)
- Studies must be among (first or second generation) migrants of any age to any of the 31 EU/EEA countries
- Studies must report prevalence by country of birth/ethnicity/region
- Studies can be among any migrant sub-population such as refugees, asylum seekers and undocumented migrants
- Studies can be among adults, pregnant women and/or children/adolescents

Exclude:

- Guidelines, opinion pieces or commentaries that are not original research of prevalence estimates
- Studies in Roma populations unless explicitly about Roma groups that are migrants (i.e. with a country of birth outside the ‘host’ country)
- Self-reported HBsAg/anti-HCV status
- Studies with unspecified virological markers of hepatitis B/C infection
- Modelling studies with no original data
- Environmental studies, technology assessments (studies on diagnostic and/or laboratory methods)

# Annex 7: Comparison of anti-HCV estimates from SRs/MAs and rationale for estimate selected for analysis

Studies compared:

**Bruggmann P**, Berg T, Ovrehus AL, Moreno C, Brandao Mello CE, Roudot-Thoraval F, et al. Historical epidemiology of hepatitis C virus (HCV) in selected countries. J Viral Hepat. 2014; 21 Suppl 1:5-33. Epub 2014/04/10;

**Cornberg, M**., H. A. Razavi, et al. (2011). A systematic review of hepatitis C virus epidemiology in Europe, Canada and Israel. Liver Int 31(SUPPL. 2): 30-60.

**Ezzikouri S**, Pineau P, Benjelloun S. Hepatitis C virus infection in the Maghreb region.J Med Virol. 2013; 85(9):1542-9;

**Gower E**, Estes C, Blach S, Razavi-Shearer K, Razavi H. Global epidemiology and genotype distribution of the hepatitis C virus infection. J Hepatol. 2014. Epub 2014/08/03;

**Hope, V. D**., I. Eramova, et al. (2014). Prevalence and estimation of hepatitis B and C infections in the WHO European Region: a review of data focusing on the countries outside the European Union and the European Free Trade Association. Epidemiol Infect 142(2): 270-286

**Kershenobich, D**., H. A. Razavi, et al. (2011). Trends and projections of hepatitis C virus epidemiology in Latin America. Liver Int 31(SUPPL. 2): 18-29;

**Mohd Hanafiah K**, Groeger J, Flaxman AD, Wiersma ST. Global epidemiology of hepatitis C virus infection: new estimates of age-specific antibody to HCV seroprevalence. Hepatology. 2013; 57(4):1333-42. Epub 2012/11/23;

**Sievert, W**., I. Altraif, et al. (2011). A systematic review of hepatitis C virus epidemiology in Asia, Australia and Egypt. Liver Int 31(SUPPL. 2): 61-80;

**Waheed Y**, Shafi T, Safi SZ, Qadri I. Hepatitis C virus in Pakistan: A systematic review of prevalence, genotypes and risk factors. World J Gastroenterol. 2009; 15(45):5647-53.

Note on Gower et al methodology: Studies in non-representative (high-risk) populations, studies with a small sample size (<1000) and studies published prior to 2000 were excluded from their analysis. Studies were given a quality score in which the representativeness for the general population, the sample size and the year of the study (with recent studies scoring higher) were taken into account. The estimates from the studies with the highest score were selected as the in-country general population prevalence estimate, with the exception of China, and India and for which estimates were calculated from a meta-analysis from multiple studies. Because most studies reported the prevalence in adults, the prevalence estimates from Gower are applicable to the adult population, defined as 15 years and older. Where studies reported the prevalence for the total population this was recalculated to a prevalence in adults.

Note on Kershenobich et al study: There is no additional or higher quality data added for the countries reported in this review; either imputed estimates, selection bias and date range exist.

Algeria
Ezzikouri provided a prevalence estimate of 1.4, which is the same as the country estimate in Gower. We therefore select the Gower estimate as we do have a 95% CI, where there is not one for the Ezzikouri estimate.

Albania
No in-country estimates are available and as Mohd Hanafiah meta-analysed more than one study (compared to Gower’s selection of the most recent and highest quality), we select the former estimate for Central Europe.

Argentina
Comparable estimate but as only a range is reported with no point estimate, we select Gower.

Belgium
Hope provides an estimate of 0.6 although the source is unclear. The Gower estimate is the same as the Bruggman estimate (0.9), we therefore take the Gower estimate.

Bulgaria
Comparable estimate, we therefore take Gower.

Brazil
Seems lower but as only a range is reported with no point estimate, we select Gower.

Egypt
Comparable estimate, although the Lehman study includes many more studies than Gower. We therefore select Lehman.

France
All estimates are derived from the same original data. Cornberg reported the prevalence in the original study. Bruggman modelled the original data by age to arrive at a lower estimate (of 0.7 compared to 0.84 in the original study). The Gower estimate used Bruggman and the original data alongside an expert panel to revise it down further (0.58). We therefore select the Bruggman estimate as it is accounts for the change in age distribution over the period 2004-2014. We also select the Bruggman CI.

Libya
Comparable estimate, we therefore take Gower.

Georgia
Comparable estimate, we therefore take Gower.

Germany
The Bruggman study is based on seven single study estimates, and is the basis of the Gower estimates. We therefore select Bruggman estimate and the CI.

Greece
There are no precise estimates from the other review available (Bruggman). We therefore select Gower.

Italy
There is quite a large difference between the estimates from Gower and Cornberg (2 to 5.2). It is unclear what the source of original data for the point estimate Gower is, although the low and high estimate ranges come from the same original study as that preferred by Cornberg (Ansaldi et al). The summary of data sources listed in Cornberg suggests that there are large age and region specific differences and that the studies took place over the last 20-30 years. Most reliable seems to be the age-adjusted estimate reported in Cornberg of 4.4 (and from Ansaldi), with the CI limit to account for the regional variation 1.6 (north) – 7.3 (south) (as reported in Gower).

Kazakhstan
The Hope estimate of 1.0 is from a study among pregnant women, a likely under-estimation. The Gower estimate seems a more reasonable estimate, and we therefore keep that although the data source for the point estimate is unclear.

Kyrgyzstan
The Hope estimate of 1.6 is from a study among pregnant women, a likely under-estimation. The Gower estimate seems a more reasonable estimate, and we therefore keep that although the data source for the point estimate is unclear.

Mauritania
Comparable estimate, we therefore take Gower.

Morocco
Comparable estimate, we therefore take Gower.

Mexico
Comparable estimate, we therefore take Gower.

The Netherlands
The estimate that is used to generate the prevalence in Gower is from a large population based study published recently whereas the Hope review is based on older estimates. We therefore take Gower.

Pakistan

Two estimates are available. The confidence limit in the Ali study is (2009) unreasonably wide. The Waheed review includes 10 studies and produced a narrow CI range. The Gower estimate in based on one estimate and we therefore select Waheed (2009).

Portugal
The estimate in the Bruggman study is from 1995 and is used within the Gower estimate. We prefer Gower.

Poland
There are a number of studies included in the description of data sources in Cornberg study whereas the Gower reports estimates from a very recent study (from 2012) which reports an age-standardised estimate for the population 2010. There is quite a difference: 0.9 compared to 1.9 in Cornberg. In the original study preferred by Gower (in Polish: Godzik, P., A. Kolakowska, et al. [Prevalence of anti-HCV antibodies among adults in Poland--results of cross-sectional study in general population]. Przegl Epidemiol 2012 66(4): 575-580), there are a number of different results reported (varying between 0.9 -1.1) due to the ELISA/Western Blot confirmation variance in samples. If ELISA and Western blot are positive, the prevalence is 1.12. Four of the studies listed in Cornberg find a prevalence of 1.9, with other estimates of 1.4 and 2.1. We select the most recent point estimate of 1.12 with the lower range as reported in Gower 0.59 and the upper range of 1.9 from Cornberg to account for this heterogeneity.

Romania
The Hope and Cornberg studies are very similar to Gower. We therefore select the Gower estimate.

Russia
The Cornberg estimates are mostly derived from older estimates, before the dissolution of the Soviet Union. Both the Hope and the Gower estimates are based on one study; we therefore select the Gower estimate which is more recent.

Serbia
The estimate from Hope is based on one study (from Kosovo), mostly in blood donors. There are only regional estimates in Gower and Mohd Hanafiah – further decision needed on preference of regional estimate.

South Korea
There is no CI in Sievert and the Gower estimate is a very recent estimate from a peer reviewed sample.

Spain
There are mostly old estimates used in the Cornberg estimates. The Bruggman study revised the estimates from these early studies down to 1.5 which was used as a reference in Gower, plus another study by the research team. We prefer Gower.

Sweden
Prefer Bruggman due to inclusion of notification and published data. The estimates are quite similar.

Syria
There is heterogeneity in the samples listed in Sievert, and the overall point estimate does not have a CI. Although the Gower estimate is for the region, it does have a CI. We prefer Gower.

Tajikistan
The estimates in blood donors and the general population from Hope are very different (5.9 compared to 0.5). The Gower estimates are between these two and considered more relevant.

Turkey
The Hope study is based on multiple estimates although it is unclear how the 0.7 estimate was derived. The Bruggman study reports the regional variety in studies, which are the low and high ranges around the Gower estimate. The Bruggman review highlights the nationally representative study used to derive the Gower estimate. We therefore select the Bruggman estimate and CI as these derive from the nationally representative recent study.

Tunisia
Comparable estimate, we therefore take Gower.

Ukraine
The estimate used in Hope is from an unpublished national source. The estimate is also much higher than in Gower (12.0 vs 3.6), which is also difficult to identify the original source of. The estimate in Hope among blood donors (2.7) is so much lower than the general population. The Hope estimate is not therefore deemed more accurate and we select Gower.

The UK
The estimates in Gower and Bruggman are derived from the same source (Public Health England). But the age distribution and the date ranges differ. The Bruggman estimate is also for England and Wales only. We therefore select Gower, which includes Scotland.

Uzbekistan
The Hope general population estimate appears to be the higher limit in the Gower estimate and the blood donor limit is the lower Gower limit. We select Gower given they derive from the same study.

Vietnam
There is heterogeneity in the samples listed in Sievert, and the overall point estimate does not have a CI. Although the Gower estimate is for the region, it and does have a CI. We prefer Gower.

# Annex 8: Anti-HCV prevalence estimates selected from systematic reviews

| **Country** | **Anti-HCV Prevalence** | **Lower limit** | **Upper limit** | **Endemicity*** | **Level of data** | **Source** | **GBD Region** |
| --- | --- | --- | --- | --- | --- | --- | --- |
| Afghanistan | 1.1 | 0.6 | 1.9 | high | Country | Gower et.al. | South Asia |
| Albania | 2.4 | 2.0 | 2.8 | high | Regional | Mohd Hanafiah et.al. | Central Europe |
| Algeria | 1.4 | 0.2 | 2.5 | high | Country | Gower et.al. | North Africa / Middle East |
| Angola | 4.2 | 2.4 | 9.2 | high | Regional | Gower et.al. | Central Sub-Saharan Africa |
| Anguilla | 0.8 | 0.2 | 1.3 | low | Regional | Gower et.al. | Caribbean |
| Antigua and Barbuda | 0.8 | 0.2 | 1.3 | low | Regional | Gower et.al. | Caribbean |
| Argentina | 1.5 | 0.5 | 2.5 | high | Country | Gower et.al. | Southern Latin America |
| Armenia | 5.4 | 3.5 | 6.8 | high | Regional | Gower et.al. | Central Asia |
| Aruba | 0.8 | 0.2 | 1.3 | low | Regional | Gower et.al. | Caribbean |
| Australia | 1.7 | 1.2 | 2.3 | high | Country | Gower et.al. | Australasia |
| Austria | 0.5 | 0.1 | 0.7 | low | Country | Gower et.al. | Western Europe |
| Azerbaijan | 3.1 | 1.0 | 6.7 | high | Country | Gower et.al. | Central Asia |
| Bahamas | 0.8 | 0.2 | 1.3 | low | Regional | Gower et.al. | Caribbean |
| Bahrain | 3.1 | 2.5 | 3.9 | high | Regional | Gower et.al. | North Africa / Middle East |
| Bangladesh | 1.3 | 0.2 | 2.2 | high | Country | Gower et.al. | South Asia |
| Barbados | 0.8 | 0.2 | 1.3 | low | Regional | Gower et.al. | Caribbean |
| Belarus | 1.3 | 0.9 | 2.9 | high | Country | Gower et.al. | Eastern Europe |
| Belgium | 0.9 | 0.1 | 1.2 | low | Country | Gower et.al. | Western Europe |
| Belize | 0.8 | 0.2 | 1.3 | low | Regional | Gower et.al. | Caribbean |
| Benin | 3.6 | 3.6 | 12.8 | high | Country | Gower et.al. | West Sub-Saharan Africa |
| Bermuda | 0.8 | 0.2 | 1.3 | low | Regional | Gower et.al. | Caribbean |
| Bhutan | 1.1 | 0.7 | 1.5 | high | Regional | Gower et.al. | South Asia |
| Bolivia | 0.9 | 0.4 | 1.3 | low | Regional | Gower et.al. | Andean Latin America |
| Bosnia and Herzegovina | 1.3 | 1.1 | 1.6 | high | Regional | Gower et.al. | Central Europe |
| Botswana | 1.3 | 0.8 | 2.5 | high | Regional | Gower et.al. | South Sub-Saharan Africa |
| Brazil | 1.6 | 1.1 | 1.6 | high | Country | Gower et.al. | Tropical Latin America |
| British Virgin Islands | 0.8 | 0.2 | 1.3 | low | Regional | Gower et.al. | Caribbean |
| Brunei Darussalam | 1.1 | 0.5 | 1.7 | high | Regional | Gower et.al. | High-Income Asia Pacific |
| Bulgaria | 1.1 | 0.3 | 2.4 | high | Country | Gower et.al. | Central Europe |
| Burkina Faso | 5.3 | 2.9 | 9.1 | high | Regional | Gower et.al. | West Sub-Saharan Africa |
| Burundi | 1.0 | 0.6 | 3.1 | high | Regional | Gower et.al. | East Sub-Saharan Africa |
| Cambodia | 2.3 | 2.3 | 14.7 | high | Country | Gower et.al. | Southeast Asia |
| Cameroon | 11.6 | 4.3 | 29.7 | high | Country | Gower et.al. | West Sub-Saharan Africa |
| Canada | 1.1 | 0.6 | 1.3 | high | Country | Gower et.al. | High-income North America |
| Cape Verde | 5.3 | 2.9 | 9.1 | high | Regional | Gower et.al. | West Sub-Saharan Africa |
| Cayman Islands | 0.8 | 0.2 | 1.3 | low | Regional | Gower et.al. | Caribbean |
| Cent African Rep | 4.2 | 2.4 | 9.2 | high | Regional | Gower et.al. | Central Sub-Saharan Africa |
| Chad | 5.3 | 2.9 | 9.1 | high | Regional | Gower et.al. | West Sub-Saharan Africa |
| Chile | 1.2 | 0.5 | 2.1 | high | Regional | Gower et.al. | Southern Latin America |
| China | 1.3 | 0.4 | 2.0 | high | Country | Gower et.al. | East Asia |
| Colombia | 1.0 | 0.8 | 1.4 | high | Regional | Gower et.al. | Central Latin America |
| Comoros | 1.0 | 0.6 | 3.1 | high | Regional | Gower et.al. | East Sub-Saharan Africa |
| Congo | 4.2 | 2.4 | 9.2 | high | Regional | Gower et.al. | Central Sub-Saharan Africa |
| Cook Islands | 0.1 | 0.1 | 0.6 | low | Regional | Gower et.al. | Oceania |
| Costa Rica | 1.0 | 0.8 | 1.4 | high | Regional | Gower et.al. | Central Latin America |
| Cote d'Ivoire | 3.3 | 0.8 | 12.8 | high | Country | Gower et.al. | West Sub-Saharan Africa |
| Croatia | 1.3 | 1.1 | 1.6 | high | Regional | Gower et.al. | Central Europe |
| Cuba | 0.8 | 0.2 | 1.3 | low | Regional | Gower et.al. | Caribbean |
| Cyprus | 0.6 | 0.5 | 1.9 | low | Country | Gower et.al. | Western Europe |
| Czech Republic | 0.7 | 0.2 | 0.7 | low | Country | Gower et.al. | Central Europe |
| Czech Republic and Slovakia | 1.3 | 1.1 | 1.6 | high | Regional | Gower et.al. | Central Europe |
| Democratic Republic of the Congo | 4.3 | 3.2 | 13.7 | high | Country | Gower et.al. | Central Sub-Saharan Africa |
| Denmark | 0.7 | 0.5 | 0.7 | low | Country | Gower et.al. | Western Europe |
| Djibouti | 1.0 | 0.6 | 3.1 | high | Regional | Gower et.al. | East Sub-Saharan Africa |
| Dominica | 0.8 | 0.2 | 1.3 | low | Regional | Gower et.al. | Caribbean |
| Dominican Republic | 0.8 | 0.2 | 1.3 | low | Regional | Gower et.al. | Caribbean |
| Ecuador | 0.9 | 0.4 | 1.3 | low | Regional | Gower et.al. | Andean Latin America |
| Egypt | 15.7 | 13.9 | 17.5 | high | Country | Lehman et.al. | North Africa / Middle East |
| El Salvador | 1.0 | 0.8 | 1.4 | high | Regional | Gower et.al. | Central Latin America |
| Equatorial Guinea | 4.2 | 2.4 | 9.2 | high | Regional | Gower et.al. | Central Sub-Saharan Africa |
| Eritrea | 1.0 | 0.6 | 3.1 | high | Regional | Gower et.al. | East Sub-Saharan Africa |
| Estonia | 3.3 | 1.6 | 4.5 | high | Regional | Gower et.al. | Eastern Europe |
| Ethiopia | 1.3 | 0.7 | 5.8 | high | Country | Gower et.al. | East Sub-Saharan Africa |
| Faeroe Islands (DK) | 0.9 | 0.7 | 1.5 | low | Regional | Gower et.al. | Western Europe |
| Fiji | 0.1 | 0.1 | 0.6 | low | Regional | Gower et.al. | Oceania |
| Finland | 0.7 | 0.6 | 0.9 | low | Country | Gower et.al. | Western Europe |
| Former Netherlands Antilles | 0.8 | 0.2 | 1.3 | low | Regional | Gower et.al. | Caribbean |
| Former Yugoslav Republic | 1.3 | 1.1 | 1.6 | high | Regional | Gower et.al. | Central Europe |
| Former USSR | 3.3 | 1.6 | 4.5 | high | Regional | Gower et.al. | Eastern Europe |
| France | 0.7 | 0.5 | 0.8 | low | Country | Bruggman et.al. | Western Europe |
| French Guiana | 0.8 | 0.2 | 1.3 | low | Regional | Gower et.al. | Caribbean |
| Gabon | 11.2 | 2.1 | 20.7 | high | Country | Gower et.al. | Central Sub-Saharan Africa |
| Gambia | 2.1 | 1.4 | 2.9 | high | Country | Gower et.al. | West Sub-Saharan Africa |
| Georgia | 6.7 | 5.6 | 7.3 | high | Country | Gower et.al. | Central Asia |
| Germany | 0.5 | 0.3 | 0.9 | low | Country | Bruggman et. al. | Western Europe |
| Ghana | 5.3 | 2.9 | 9.1 | high | Regional | Gower et.al. | West Sub-Saharan Africa |
| Gibraltar | 0.9 | 0.7 | 1.5 | low | Regional | Gower et.al. | Western Europe |
| Greece | 1.9 | 0.5 | 2.6 | high | Country | Gower et.al. | Western Europe |
| Greenland (DK) | 0.9 | 0.7 | 1.5 | low | Regional | Gower et.al. | Western Europe |
| Grenada | 0.8 | 0.2 | 1.3 | low | Regional | Gower et.al. | Caribbean |
| Guatemala | 1.0 | 0.8 | 1.4 | high | Regional | Gower et.al. | Central Latin America |
| Guinea | 5.3 | 2.9 | 9.1 | high | Regional | Gower et.al. | West Sub-Saharan Africa |
| Guinea-Bissau | 5.3 | 2.9 | 9.1 | high | Regional | Gower et.al. | West Sub-Saharan Africa |
| Guyana | 0.8 | 0.2 | 1.3 | low | Regional | Gower et.al. | Caribbean |
| Haiti | 0.8 | 0.2 | 1.3 | low | Regional | Gower et.al. | Caribbean |
| Holy See | 0.9 | 0.7 | 1.5 | low | Regional | Gower et.al. | Western Europe |
| Honduras | 1.0 | 0.8 | 1.4 | high | Regional | Gower et.al. | Central Latin America |
| Hong Kong | 1.2 | 0.4 | 1.8 | high | Regional | Gower et.al. | East Asia |
| Hungary | 0.8 | 0.4 | 2.7 | low | Country | Gower et.al. | Central Europe |
| Iceland | 0.9 | 0.7 | 1.5 | low | Regional | Gower et.al. | Western Europe |
| India | 0.8 | 0.4 | 1.0 | low | Country | Gower et.al. | South Asia |
| Indonesia | 0.8 | 0.4 | 2.0 | low | Country | Gower et.al. | Southeast Asia |
| Iran | 0.5 | 0.2 | 1.0 | low | Country | Gower et.al. | North Africa / Middle East |
| Iraq | 3.2 | 0.3 | 3.2 | high | Country | Gower et.al. | North Africa / Middle East |
| Ireland | 1.1 | 0.7 | 1.6 | high | Country | Gower et.al. | Western Europe |
| Isle of Man | 0.9 | 0.7 | 1.5 | low | Regional | Gower et.al. | Western Europe |
| Israel | 2.0 | 0.9 | 2.0 | high | Country | Gower et.al. | Western Europe |
| Italy | 4.4 | 1.6 | 7.3 | high | Country | Cornberg et. al. | Western Europe |
| Ivory Coast | 5.3 | 2.9 | 9.1 | high | Regional | Gower et.al. | West Sub-Saharan Africa |
| Jamaica | 0.8 | 0.2 | 1.3 | low | Regional | Gower et.al. | Caribbean |
| Japan | 1.5 | 0.5 | 2.2 | high | Country | Gower et.al. | High-Income Asia Pacific |
| Jordan | 3.1 | 2.5 | 3.9 | high | Regional | Gower et.al. | North Africa / Middle East |
| Kazakhstan | 3.3 | 1.0 | 6.7 | high | Country | Gower et.al. | Central Asia |
| Kenya | 1.0 | 0.6 | 3.1 | high | Regional | Gower et.al. | East Sub-Saharan Africa |
| Kiribati | 0.1 | 0.1 | 0.6 | low | Regional | Gower et.al. | Oceania |
| Kosovo | 1.3 | 1.1 | 1.6 | high | Regional | Gower et.al. | Central Europe |
| Kuwait | 3.1 | 2.5 | 3.9 | high | Regional | Gower et.al. | North Africa / Middle East |
| Kyrgyzstan | 2.5 | 1.6 | 6.7 | high | Country | Gower et.al. | Central Asia |
| Laos | 1.0 | 0.8 | 1.8 | high | Regional | Gower et.al. | Southeast Asia |
| Latvia | 2.4 | 1.7 | 3.3 | high | Country | Gower et.al. | Eastern Europe |
| Lebanon | 3.1 | 2.5 | 3.9 | high | Regional | Gower et.al. | North Africa / Middle East |
| Lesotho | 1.3 | 0.8 | 2.5 | high | Regional | Gower et.al. | South Sub-Saharan Africa |
| Liberia | 5.3 | 2.9 | 9.1 | high | Regional | Gower et.al. | West Sub-Saharan Africa |
| Libya | 1.2 | 1.2 | 2.3 | high | Country | Gower et.al. | North Africa / Middle East |
| Liechtenstein | 0.9 | 0.7 | 1.5 | low | Regional | Gower et.al. | Western Europe |
| Lithuania | 2.9 | 0.7 | 3.0 | high | Country | Gower et.al. | Eastern Europe |
| Luxembourg | 0.9 | 0.6 | 0.9 | low | Country | Gower et.al. | Western Europe |
| Macao, China | 1.2 | 0.4 | 1.8 | high | Regional | Gower et.al. | East Asia |
| Macedonia | 1.3 | 1.1 | 1.6 | high | Regional | Gower et.al. | Central Europe |
| Madagascar | 1.2 | 0.8 | 1.7 | high | Country | Gower et.al. | East Sub-Saharan Africa |
| Malawi | 1.0 | 0.6 | 3.1 | high | Regional | Gower et.al. | East Sub-Saharan Africa |
| Malaysia | 1.5 | 0.3 | 7.7 | high | Country | Gower et.al. | Southeast Asia |
| Maldives | 1.0 | 0.8 | 1.8 | high | Regional | Gower et.al. | Southeast Asia |
| Mali | 5.3 | 2.9 | 9.1 | high | Regional | Gower et.al. | West Sub-Saharan Africa |
| Malta | 0.9 | 0.7 | 1.5 | low | Regional | Gower et.al. | Western Europe |
| Marshall Islands | 0.1 | 0.1 | 0.6 | low | Regional | Gower et.al. | Oceania |
| Mauritania | 1.9 | 1.1 | 10.7 | high | Country | Gower et.al. | West Sub-Saharan Africa |
| Mauritius | 1.0 | 0.8 | 1.8 | high | Regional | Gower et.al. | Southeast Asia |
| Mexico | 1.4 | 1.1 | 1.6 | high | Country | Gower et.al. | Central Latin America |
| Micronesia (Federated States of) | 0.1 | 0.1 | 0.6 | low | Regional | Gower et.al. | Oceania |
| Moldova | 3.3 | 1.6 | 4.5 | high | Regional | Gower et.al. | Eastern Europe |
| Monaco | 0.9 | 0.7 | 1.5 | low | Regional | Gower et.al. | Western Europe |
| Mongolia | 10.8 | 8.7 | 15.6 | high | Country | Gower et.al. | Central Asia |
| Montenegro | 1.3 | 1.1 | 1.6 | high | Regional | Gower et.al. | Central Europe |
| Montserrat | 0.8 | 0.2 | 1.3 | low | Regional | Gower et.al. | Caribbean |
| Morocco | 1.6 | 0.6 | 1.9 | high | Country | Gower et.al. | North Africa / Middle East |
| Mozambique | 1.0 | 0.6 | 3.1 | high | Regional | Gower et.al. | East Sub-Saharan Africa |
| Myanmar | 1.7 | 1.0 | 2.7 | high | Country | Gower et.al. | Southeast Asia |
| Namibia | 1.3 | 0.8 | 2.5 | high | Regional | Gower et.al. | South Sub-Saharan Africa |
| Nauru | 0.1 | 0.1 | 0.6 | low | Regional | Gower et.al. | Oceania |
| Nepal | 1.1 | 0.7 | 1.5 | high | Regional | Gower et.al. | South Asia |
| Netherlands | 0.2 | 0.1 | 0.4 | low | Country | Gower et.al. | Western Europe |
| New Zealand | 1.9 | 0.8 | 2.2 | high | Country | Gower et.al. | Australasia |
| Nicaragua | 1.0 | 0.8 | 1.4 | high | Regional | Gower et.al. | Central Latin America |
| Niger | 5.3 | 2.9 | 9.1 | high | Regional | Gower et.al. | West Sub-Saharan Africa |
| Nigeria | 8.4 | 3.9 | 12.8 | high | Country | Gower et.al. | West Sub-Saharan Africa |
| Niue | 0.1 | 0.1 | 0.6 | low | Regional | Gower et.al. | Oceania |
| Norway | 0.7 | 0.6 | 0.9 | low | Country | Gower et.al. | Western Europe |
| Oman | 3.1 | 2.5 | 3.9 | high | Regional | Gower et.al. | North Africa / Middle East |
| Pakistan | 5.0 | 4.4 | 5.5 | high | Country | Waheed et. al. | South Asia |
| Palau | 0.1 | 0.1 | 0.6 | low | Regional | Gower et.al. | Oceania |
| Palestine | 3.1 | 2.5 | 3.9 | high | Regional | Gower et.al. | North Africa / Middle East |
| Panama | 1.0 | 0.8 | 1.4 | high | Regional | Gower et.al. | Central Latin America |
| Papua New Guinea | 0.1 | 0.1 | 0.6 | low | Regional | Gower et.al. | Oceania |
| Paraguay | 1.2 | 0.9 | 1.2 | high | Regional | Gower et.al. | Tropical Latin America |
| People's Republic of Korea | 1.2 | 0.4 | 1.8 | high | Regional | Gower et.al. | East Asia |
| Peru | 1.2 | 0.4 | 1.6 | high | Country | Gower et.al. | Andean Latin America |
| Philippines | 0.9 | 0.3 | 2.0 | low | Country | Gower et.al. | Southeast Asia |
| Poland | 1.1 | 0.6 | 1.9 | high | Country | Godzik et. al. | Central Europe |
| Portugal | 1.8 | 0.5 | 2.9 | high | Country | Gower et.al. | Western Europe |
| Puerto Rico | 2.3 | 1.3 | 4.2 | high | Country | Gower et.al. | Caribbean |
| Qatar | 0.9 | 0.5 | 1.5 | low | Country | Gower et.al. | North Africa / Middle East |
| Rep, of Moldova | 4.5 | 2.3 | 4.5 | high | Country | Gower et.al. | Eastern Europe |
| Republic of Korea | 0.8 | 0.2 | 2.1 | high | Country | Gower et.al. | High-Income Asia Pacific |
| Romania | 3.2 | 2.9 | 3.6 | high | Country | Gower et.al. | Central Europe |
| Russia | 4.1 | 1.2 | 5.6 | high | Country | Gower et.al. | Eastern Europe |
| Rwanda | 1.0 | 0.6 | 3.1 | high | Regional | Gower et.al. | East Sub-Saharan Africa |
| Saint Kitts and Nevis | 0.8 | 0.2 | 1.3 | low | Regional | Gower et.al. | Caribbean |
| Saint Lucia | 0.8 | 0.2 | 1.3 | low | Regional | Gower et.al. | Caribbean |
| Saint Vincent and the Grenadines | 0.8 | 0.2 | 1.3 | low | Regional | Gower et.al. | Caribbean |
| Samoa | 0.2 | 0.2 | 0.9 | low | Country | Gower et.al. | Oceania |
| San Marino | 0.9 | 0.7 | 1.5 | low | Regional | Gower et.al. | Western Europe |
| Sao Tome and Principe | 5.3 | 2.9 | 9.1 | high | Regional | Gower et.al. | West Sub-Saharan Africa |
| Saudi Arabia | 1.5 | 0.6 | 7.3 | high | Country | Gower et.al. | North Africa / Middle East |
| Senegal | 5.3 | 2.9 | 9.1 | high | Regional | Gower et.al. | West Sub-Saharan Africa |
| Serbia | 1.3 | 1.1 | 1.6 | high | Regional | Gower et.al. | Central Europe |
| Seychelles | 1.0 | 0.8 | 1.8 | high | Regional | Gower et.al. | Southeast Asia |
| Sierra Leone | 5.3 | 2.9 | 9.1 | high | Regional | Gower et.al. | West Sub-Saharan Africa |
| Singapore | 1.1 | 0.5 | 1.7 | high | Regional | Gower et.al. | High-Income Asia Pacific |
| Slovakia | 1.4 | 0.9 | 2.0 | high | Country | Gower et.al. | Central Europe |
| Slovenia | 1.3 | 1.1 | 1.6 | high | Regional | Gower et.al. | Central Europe |
| Solomon Islands | 0.1 | 0.1 | 0.6 | low | Regional | Gower et.al. | Oceania |
| Somalia | 1.0 | 0.6 | 3.1 | high | Regional | Gower et.al. | East Sub-Saharan Africa |
| South Africa | 1.7 | 1.0 | 2.5 | high | Country | Gower et.al. | South Sub-Saharan Africa |
| South Korea | 0.8 | 0.2 | 2.1 | low | Country | Gower et.al. | High-Income Asia Pacific |
| South Sudan | 1.0 | 0.6 | 3.1 | high | Regional | Gower et.al. |  |
| Spain | 1.7 | 0.4 | 2.6 | high | Country | Gower et.al. | Western Europe |
| Sri Lanka | 1.0 | 0.8 | 1.8 | high | Regional | Gower et.al. | Southeast Asia |
| Sudan | 1.0 | 0.6 | 3.1 | high | Regional | Gower et.al. | East Sub-Saharan Africa |
| Suriname | 0.8 | 0.2 | 1.3 | low | Regional | Gower et.al. | Caribbean |
| Swaziland | 1.3 | 0.8 | 2.5 | high | Regional | Gower et.al. | South Sub-Saharan Africa |
| Sweden | 0.6 | 0.5 | 0.7 | low | Country | Bruggman et. al. | Western Europe |
| Switzerland | 1.5 | 0.7 | 1.8 | high | Country | Gower et.al. | Western Europe |
| Syria | 3.1 | 2.5 | 3.9 | high | Regional | Gower et.al. | North Africa / Middle East |
| Taiwan | 4.4 | 2.5 | 6.3 | high | Country | Gower et.al. | East Asia |
| Tajikistan | 3.1 | 1.1 | 6.7 | high | Country | Gower et.al. | Central Asia |
| Tanzania | 1.0 | 0.6 | 3.1 | high | Regional | Gower et.al. | East Sub-Saharan Africa |
| Thailand | 2.7 | 1.8 | 3.7 | high | Country | Gower et.al. | Southeast Asia |
| Timor-Leste | 1.0 | 0.8 | 1.8 | high | Regional | Gower et.al. | Southeast Asia |
| Togo | 5.3 | 2.9 | 9.1 | high | Regional | Gower et.al. | West Sub-Saharan Africa |
| Tokelau | 0.1 | 0.1 | 0.6 | low | Regional | Gower et.al. | Oceania |
| Tonga | 0.1 | 0.1 | 0.6 | low | Regional | Gower et.al. | Oceania |
| Trinidad and Tobago | 0.8 | 0.2 | 1.3 | low | Regional | Gower et.al. | Caribbean |
| Tunisia | 1.3 | 0.3 | 2.5 | high | Country | Gower et.al. | North Africa / Middle East |
| Turkey | 1.0 | 0.7 | 1.1 | high | Country | Bruggman et. al. | North Africa / Middle East |
| Turkmenistan | 5.6 | 1.1 | 6.7 | high | Country | Gower et.al. | Central Asia |
| Turks and Caicos Islands | 0.8 | 0.2 | 1.3 | low | Regional | Gower et.al. | Caribbean |
| Tuvalu | 0.1 | 0.1 | 0.6 | low | Regional | Gower et.al. | Oceania |
| Uganda | 1.0 | 0.6 | 3.1 | high | Regional | Gower et.al. | East Sub-Saharan Africa |
| Ukraine | 3.6 | 0.9 | 4.5 | high | Country | Gower et.al. | Eastern Europe |
| United Arab Emirates | 3.1 | 2.5 | 3.9 | high | Regional | Gower et.al. | North Africa / Middle East |
| United Kingdom | 0.6 | 0.4 | 1.2 | low | Country | Gower et.al. | Western Europe |
| United States | 1.3 | 1.2 | 2.4 | high | Country | Gower et.al. | High-income North America |
| Uruguay | 1.2 | 0.5 | 2.1 | high | Regional | Gower et.al. | Southern Latin America |
| US Virgin Islands | 0.8 | 0.2 | 1.3 | low | Regional | Gower et.al. | Caribbean |
| Uzbekistan | 11.3 | 6.4 | 13.1 | high | Country | Gower et.al. | Central Asia |
| Vanuatu | 0.1 | 0.1 | 0.6 | low | Regional | Gower et.al. | Oceania |
| Venezuela | 1.5 | 0.3 | 2.6 | high | Country | Gower et.al. | Central Latin America |
| Vietnam | 1.0 | 0.8 | 1.8 | high | Regional | Gower et.al. | Southeast Asia |
| Western Sahara | 3.1 | 2.5 | 3.9 | high | Regional | Gower et.al. | North Africa / Middle East |
| Yemen | 2.2 | 1.1 | 3.5 | high | Country | Gower et.al. | North Africa / Middle East |
| Yugoslavia | 1.3 | 1.1 | 1.6 | high | Regional | Gower et.al. | Central Europe |
| Zambia | 1.0 | 0.6 | 3.1 | high | Regional | Gower et.al. | East Sub-Saharan Africa |
| Zimbabwe | 1.6 | 1.0 | 9.1 | high | Country | Gower et.al. | South Sub-Saharan Africa |

*High endemiciy classified as >1% prevalence

# Annex 9: Chronic Hepatitis C burden country tables: 50 largest migrant populations per EU/EEA country

The following country tables list the estimated number of Chronic Hepatitis C (CHC) cases among the 50 largest adult (>15 years) migrant populations residing in the individual EU/EEA host countries. The tables are sorted in descending order by the absolute number of CHC cases, and first list populations from high endemic countries (anti-HCV prevalence ≥1%), followed by populations from low endemic countries.

Notes on dissolved states/former nation states listed:

China includes the territory of Hong Kong

Kosovo refers to the territory as per UN SCR 1244/99

Former Czechoslovakia refers to the nation state area of Czechoslovakia prior to 1993

Former Soviet Union refers to the nation state of the USSR prior to 1991

Former Yugoslavia refers to the nation state of Yugoslavia prior to 1992

Former Yugoslav Republic refers to the nation state established between 1992 to 2006 (https://en.wikipedia.org/wiki/Serbia_and_Montenegro)

## Austria

| **Country of birth** | **Population (>15 yrs)** | **Anti-HCV endemicity** | **Anti-HCV prevalence** | | | **Estimated no. of CHC cases** | | |
| --- | --- | --- | --- | --- | --- | --- | --- | --- |
|  |  |  | **%** | **Lower limit** | **Upper limit** | **Cases** | **Lower range** | **Upper range** |
| **Romania** | 69,506 | high | 3.2 | 2.9 | 3.6 | 1,557 | 1,411 | 1,752 |
| **Bosnia and Herzegovina** | 149,265 | high | 1.3 | 1.1 | 1.6 | 1,358 | 1,149 | 1,672 |
| **Egypt** | 11,612 | high | 15.7 | 13.9 | 17.5 | 1,276 | 1,130 | 1,422 |
| **Serbia** | 126,453 | high | 1.3 | 1.1 | 1.6 | 1,151 | 974 | 1,416 |
| **Turkey** | 154,707 | high | 1 | 0.7 | 1.1 | 1,083 | 758 | 1,191 |
| **Italy** | 24,638 | high | 4.4 | 1.6 | 7.3 | 759 | 276 | 1,259 |
| **Russia** | 24,206 | high | 4.1 | 1.2 | 5.6 | 695 | 203 | 949 |
| **Poland** | 60,323 | high | 1.1 | 0.6 | 1.9 | 464 | 253 | 802 |
| **Nigeria** | 6,476 | high | 8.4 | 3.9 | 12.8 | 381 | 177 | 580 |
| **Croatia** | 38,305 | high | 1.3 | 1.1 | 1.6 | 349 | 295 | 429 |
| **Slovakia** | 27,776 | high | 1.4 | 0.9 | 2 | 272 | 175 | 389 |
| **Kosovo** | 26,251 | high | 1.3 | 1.1 | 1.6 | 239 | 202 | 294 |
| **Ukraine** | 8,290 | high | 3.6 | 0.9 | 4.5 | 209 | 52 | 261 |
| **FYR Macedonia** | 20,513 | high | 1.3 | 1.1 | 1.6 | 187 | 158 | 230 |
| **Slovenia** | 18,247 | high | 1.3 | 1.1 | 1.6 | 166 | 141 | 204 |
| **Pakistan** | 4,719 | high | 5 | 4.4 | 5.5 | 165 | 145 | 182 |
| **Switzerland** | 12,703 | high | 1.5 | 0.7 | 1.8 | 133 | 62 | 160 |
| **China** | 13,862 | high | 1.3 | 0.4 | 2.0 | 126 | 39 | 194 |
| **Georgia** | 2,668 | high | 6.7 | 5.6 | 7.3 | 125 | 105 | 136 |
| **Bulgaria** | 15,876 | high | 1.1 | 0.3 | 2.4 | 122 | 33 | 267 |
| **Iraq** | 4,886 | high | 3.2 | 0.3 | 3.2 | 109 | 10 | 109 |
| **Armenia** | 2,496 | high | 5.4 | 3.5 | 6.8 | 94 | 61 | 119 |
| **Thailand** | 4,805 | high | 2.7 | 1.8 | 3.7 | 91 | 61 | 124 |
| **Afghanistan** | 11,272 | high | 1.1 | 0.6 | 1.9 | 87 | 47 | 150 |
| **Syria** | 3,599 | high | 3.1 | 2.5 | 3.9 | 78 | 63 | 98 |
| **United States** | 8,056 | high | 1.3 | 1.2 | 2.4 | 73 | 68 | 135 |
| **Ghana** | 1,946 | high | 5.3 | 2.9 | 9.1 | 72 | 40 | 124 |
| **Greece** | 3,921 | high | 1.9 | 0.5 | 2.6 | 52 | 14 | 71 |
| **Albania** | 2,870 | high | 2.4 | 2 | 2.8 | 48 | 40 | 56 |
| **Brazil** | 4,272 | high | 1.6 | 1.1 | 1.6 | 48 | 33 | 48 |
| **Spain** | 3,875 | high | 1.7 | 0.4 | 2.6 | 46 | 11 | 71 |
| **Tunisia** | 3,331 | high | 1.3 | 0.3 | 2.5 | 30 | 7 | 58 |
| **South Africa** | 2,142 | high | 1.7 | 1.0 | 2.5 | 25 | 15 | 37 |
| **Japan** | 2,362 | high | 1.5 | 0.5 | 2.2 | 25 | 8 | 36 |
| **Bangladesh** | 2,341 | high | 1.3 | 0.2 | 2.2 | 21 | 3 | 36 |
| **Vietnam** | 2,790 | high | 1.0 | 0.8 | 1.8 | 20 | 16 | 35 |
| **Canada** | 1,985 | high | 1.1 | 0.6 | 1.3 | 15 | 8 | 18 |
| **Germany** | 192,124 | low | 0.5 | 0.3 | 0.9 | 672 | 403 | 1,210 |
| **Hungary** | 44,168 | low | 0.8 | 0.4 | 2.7 | 247 | 124 | 835 |
| **Czech Republic** | 40,693 | low | 0.7 | 0.2 | 0.7 | 199 | 57 | 199 |
| **Philippines** | 11,739 | low | 0.9 | 0.3 | 2.0 | 74 | 25 | 164 |
| **India** | 11,610 | low | 0.8 | 0.4 | 1.0 | 65 | 33 | 81 |
| **Iran** | 14,032 | low | 0.5 | 0.2 | 1.0 | 49 | 20 | 98 |
| **United Kingdom** | 8,654 | low | 0.6 | 0.4 | 1.2 | 36 | 24 | 73 |
| **France** | 7,186 | low | 0.7 | 0.5 | 0.8 | 35 | 25 | 40 |
| **Dominican Republic** | 2,600 | low | 0.8 | 0.2 | 1.3 | 15 | 4 | 24 |
| **Belgium** | 2,015 | low | 0.9 | 0.1 | 1.2 | 13 | 1 | 17 |
| **South Korea** | 2,081 | low | 0.8 | 0.2 | 2.1 | 12 | 3 | 31 |
| **Sweden** | 2,684 | low | 0.6 | 0.5 | 0.7 | 11 | 9 | 13 |
| **Netherlands** | 7,128 | low | 0.2 | 0.1 | 0.4 | 10 | 5 | 20 |

## Belgium

| **Country of birth** | **Population (>15 yrs)** | **Anti-HCV endemicity** | **Anti-HCV prevalence** | | | **Estimated no. of CHC cases** | | |
| --- | --- | --- | --- | --- | --- | --- | --- | --- |
|  |  |  | **%** | **Lower limit** | **Upper limit** | **Cases** | **Lower range** | **Upper range** |
| **Italy** | 115,528 | high | 4.4 | 1.6 | 7.3 | 3,558 | 1,294 | 5,903 |
| **DR Congo** | 81,383 | high | 4.3 | 3.2 | 13.7 | 2,450 | 1,823 | 7,805 |
| **Morocco** | 197,472 | high | 1.6 | 0.6 | 1.9 | 2,212 | 829 | 2,626 |
| **Former Soviet Union** | 54,604 | high | 3.3 | 1.6 | 4.5 | 1,261 | 612 | 1,720 |
| **Cameroon** | 13,519 | high | 11.6 | 4.3 | 29.7 | 1,098 | 407 | 2,811 |
| **Romania** | 47,832 | high | 3.2 | 2.9 | 3.6 | 1,071 | 971 | 1,205 |
| **Turkey** | 96,479 | high | 1.0 | 0.7 | 1.1 | 675 | 473 | 743 |
| **Poland** | 62,233 | high | 1.1 | 0.6 | 1.9 | 479 | 261 | 828 |
| **Former Yugoslavia** | 47,892 | high | 1.3 | 1.1 | 1.6 | 436 | 369 | 536 |
| **Spain** | 35,598 | high | 1.7 | 0.4 | 2.6 | 424 | 100 | 648 |
| **Guinea** | 10,957 | high | 5.3 | 2.9 | 9.1 | 407 | 222 | 698 |
| **Pakistan** | 11,481 | high | 5.0 | 4.4 | 5.5 | 402 | 354 | 442 |
| **Portugal** | 29,034 | high | 1.8 | 0.5 | 2.9 | 366 | 102 | 589 |
| **Nigeria** | 5,048 | high | 8.4 | 3.9 | 12.8 | 297 | 138 | 452 |
| **Ghana** | 7,706 | high | 5.3 | 2.9 | 9.1 | 286 | 156 | 491 |
| **Algeria** | 25,134 | high | 1.4 | 0.2 | 2.5 | 246 | 35 | 440 |
| **Iraq** | 9,435 | high | 3.2 | 0.3 | 3.2 | 211 | 20 | 211 |
| **Greece** | 14,554 | high | 1.9 | 0.5 | 2.6 | 194 | 51 | 265 |
| **Côte d'Ivoire** | 5,062 | high | 5.3 | 2.9 | 9.1 | 188 | 103 | 322 |
| **Togo** | 5,031 | high | 5.3 | 2.9 | 9.1 | 187 | 102 | 320 |
| **Angola** | 5,979 | high | 4.2 | 2.4 | 9.2 | 176 | 100 | 385 |
| **Senegal** | 4,569 | high | 5.3 | 2.9 | 9.1 | 170 | 93 | 291 |
| **Bulgaria** | 21,585 | high | 1.1 | 0.3 | 2.4 | 166 | 45 | 363 |
| **China** | 16,443 | high | 1.3 | 0.4 | 2.0 | 150 | 46 | 230 |
| **Albania** | 8,655 | high | 2.4 | 2 | 2.8 | 145 | 121 | 170 |
| **Syria** | 6,618 | high | 3.1 | 2.5 | 3.9 | 144 | 116 | 181 |
| **Thailand** | 7,222 | high | 2.7 | 1.8 | 3.7 | 136 | 91 | 187 |
| **Lebanon** | 5,865 | high | 3.1 | 2.5 | 3.9 | 127 | 103 | 160 |
| **Congo** | 4,244 | high | 4.2 | 2.4 | 9.2 | 125 | 71 | 273 |
| **Brazil** | 10,987 | high | 1.6 | 1.1 | 1.6 | 123 | 85 | 123 |
| **Tunisia** | 12,946 | high | 1.3 | 0.3 | 2.5 | 118 | 27 | 227 |
| **United States** | 12,445 | high | 1.3 | 1.2 | 2.4 | 113 | 105 | 209 |
| **Rwanda** | 13,292 | high | 1 | 0.6 | 3.1 | 93 | 56 | 288 |
| **Afghanistan** | 10,969 | high | 1.1 | 0.6 | 1.9 | 84 | 46 | 146 |
| **Former Czechoslovakia** | 8,221 | high | 1.3 | 1.1 | 1.6 | 75 | 63 | 92 |
| **Vietnam** | 7,722 | high | 1.0 | 0.8 | 1.8 | 54 | 43 | 97 |
| **Switzerland** | 4,340 | high | 1.5 | 0.7 | 1.8 | 46 | 21 | 55 |
| **Burundi** | 6,403 | high | 1.0 | 0.6 | 3.1 | 45 | 27 | 139 |
| **Colombia** | 5,318 | high | 1.0 | 0.8 | 1.4 | 37 | 30 | 52 |
| **Canada** | 4,350 | high | 1.1 | 0.6 | 1.3 | 33 | 18 | 40 |
| **France** | 164,889 | low | 0.7 | 0.5 | 0.8 | 808 | 577 | 923 |
| **Germany** | 77,037 | low | 0.5 | 0.3 | 0.9 | 270 | 162 | 485 |
| **Netherlands** | 111,552 | low | 0.2 | 0.1 | 0.4 | 156 | 78 | 312 |
| **India** | 16,949 | low | 0.8 | 0.4 | 1.0 | 95 | 47 | 119 |
| **United Kingdom** | 20,682 | low | 0.6 | 0.4 | 1.2 | 87 | 58 | 174 |
| **Philippines** | 9,187 | low | 0.9 | 0.3 | 2.0 | 58 | 19 | 129 |
| **Luxembourg** | 9,000 | low | 0.9 | 0.6 | 0.9 | 57 | 38 | 57 |
| **Hungary** | 7,222 | low | 0.8 | 0.4 | 2.7 | 40 | 20 | 136 |
| **Iran** | 10,421 | low | 0.5 | 0.2 | 1.0 | 36 | 15 | 73 |
| **Ecuador** | 5,504 | low | 0.9 | 0.4 | 1.3 | 35 | 15 | 50 |

## Bulgaria

| **Country of birth** | **Population (>15 yrs)** | **Anti-HCV endemicity** | **Anti-HCV prevalence** | | | **Estimated no. of CHC cases** | | |
| --- | --- | --- | --- | --- | --- | --- | --- | --- |
|  |  |  | **%** | **Lower limit** | **Upper limit** | **Cases** | **Lower range** | **Upper range** |
| **Russia** | 18,929 | high | 4.1 | 1.2 | 5.6 | 543 | 159 | 742 |
| **Ukraine** | 5,896 | high | 3.6 | 0.9 | 4.5 | 149 | 37 | 186 |
| **Romania** | 5,308 | high | 3.2 | 2.9 | 3.6 | 119 | 108 | 134 |
| **Greece** | 4,896 | high | 1.9 | 0.5 | 2.6 | 65 | 17 | 89 |
| **Uzbekistan** | 712 | high | 11.3 | 6.4 | 13.1 | 56 | 32 | 65 |
| **Armenia** | 1,373 | high | 5.4 | 3.5 | 6.8 | 52 | 34 | 65 |
| **Moldova** | 1,849 | high | 3.3 | 1.6 | 4.5 | 43 | 21 | 58 |
| **Azerbaijan** | 1,866 | high | 3.1 | 1.0 | 6.7 | 40 | 13 | 88 |
| **Turkey** | 4,857 | high | 1.0 | 0.7 | 1.1 | 34 | 24 | 37 |
| **Syria** | 1,191 | high | 3.1 | 2.5 | 3.9 | 26 | 21 | 33 |
| **Italy** | 769 | high | 4.4 | 1.6 | 7.3 | 24 | 9 | 39 |
| **Kazakhstan** | 1,012 | high | 3.3 | 1.0 | 6.7 | 23 | 7 | 47 |
| **FYR Macedonia** | 2,278 | high | 1.3 | 1.1 | 1.6 | 21 | 18 | 26 |
| **Serbia** | 2,167 | high | 1.3 | 1.1 | 1.6 | 20 | 17 | 24 |
| **Albania** | 1,071 | high | 2.4 | 2.0 | 2.8 | 18 | 15 | 21 |
| **Iraq** | 701 | high | 3.2 | 0.3 | 3.2 | 16 | 1 | 16 |
| **Egypt** | 135 | high | 15.7 | 13.9 | 17.5 | 15 | 13 | 17 |
| **Georgia** | 313 | high | 6.7 | 5.6 | 7.3 | 15 | 12 | 16 |
| **Lebanon** | 549 | high | 3.1 | 2.5 | 3.9 | 12 | 10 | 15 |
| **Turkmenistan** | 265 | high | 5.6 | 1.1 | 6.7 | 10 | 2 | 12 |
| **Poland** | 1,167 | high | 1.1 | 0.6 | 1.9 | 9 | 5 | 16 |
| **China** | 813 | high | 1.3 | 0.4 | 2.0 | 7 | 2 | 11 |
| **Belarus** | 637 | high | 1.3 | 0.9 | 2.9 | 6 | 4 | 13 |
| **United States** | 634 | high | 1.3 | 1.2 | 2.4 | 6 | 5 | 11 |
| **Bangladesh** | 536 | high | 1.3 | 0.2 | 2.2 | 5 | 1 | 8 |
| **Vietnam** | 568 | high | 1.0 | 0.8 | 1.8 | 4 | 3 | 7 |
| **Kosovo** | 395 | high | 1.3 | 1.1 | 1.6 | 4 | 3 | 4 |
| **Algeria** | 346 | high | 1.4 | 0.2 | 2.5 | 3 | 0 | 6 |
| **Jordan** | 149 | high | 3.1 | 2.5 | 3.9 | 3 | 3 | 4 |
| **Israel** | 217 | high | 2.0 | 0.9 | 2.0 | 3 | 1 | 3 |
| **Slovakia** | 278 | high | 1.4 | 0.9 | 2.0 | 3 | 2 | 4 |
| **Spain** | 221 | high | 1.7 | 0.4 | 2.6 | 3 | 1 | 4 |
| **Lithuania** | 128 | high | 2.9 | 0.7 | 3.0 | 3 | 1 | 3 |
| **Latvia** | 139 | high | 2.4 | 1.7 | 3.3 | 2 | 2 | 3 |
| **Afghanistan** | 271 | high | 1.1 | 0.6 | 1.9 | 2 | 1 | 4 |
| **Australia** | 129 | high | 1.7 | 1.2 | 2.3 | 2 | 1 | 2 |
| **Libya** | 176 | high | 1.2 | 1.2 | 2.3 | 1 | 1 | 3 |
| **United Kingdom** | 3,097 | low | 0.6 | 0.4 | 1.2 | 13 | 9 | 26 |
| **Germany** | 1,861 | low | 0.5 | 0.3 | 0.9 | 7 | 4 | 12 |
| **Czech Republic** | 824 | low | 0.7 | 0.2 | 0.7 | 4 | 1 | 4 |
| **France** | 469 | low | 0.7 | 0.5 | 0.8 | 2 | 2 | 3 |
| **Belgium** | 294 | low | 0.9 | 0.1 | 1.2 | 2 | 0 | 2 |
| **Hungary** | 308 | low | 0.8 | 0.4 | 2.7 | 2 | 1 | 6 |
| **Cuba** | 255 | low | 0.8 | 0.2 | 1.3 | 1 | 0 | 2 |
| **India** | 162 | low | 0.8 | 0.4 | 1.0 | 1 | 0 | 1 |
| **Iran** | 257 | low | 0.5 | 0.2 | 1.0 | 1 | 0 | 2 |
| **Cyprus** | 175 | low | 0.6 | 0.5 | 1.9 | 1 | 1 | 2 |
| **Austria** | 174 | low | 0.5 | 0.1 | 0.7 | 1 | 0 | 1 |
| **Netherlands** | 288 | low | 0.2 | 0.1 | 0.4 | 0 | 0 | 1 |

## Croatia

| **Country of birth** | **Population (>15 yrs)** | **Anti-HCV endemicity** | **Anti-HCV prevalence** | | | **Estimated no. of CHC cases** | | |
| --- | --- | --- | --- | --- | --- | --- | --- | --- |
|  |  |  | **%** | **Lower limit** | **Upper limit** | **Cases** | **Lower range** | **Upper range** |
| **Bosnia and Herzegovina** | 403,576 | high | 1.3 | 1.1 | 1.6 | 3,673 | 3,108 | 4,520 |
| **Serbia** | 51,169 | high | 1.3 | 1.1 | 1.6 | 466 | 394 | 573 |
| **Kosovo** | 19,931 | high | 1.3 | 1.1 | 1.6 | 181 | 153 | 223 |
| **Slovenia** | 19,276 | high | 1.3 | 1.1 | 1.6 | 175 | 148 | 216 |
| **FYR Macedonia** | 9,887 | high | 1.3 | 1.1 | 1.6 | 90 | 76 | 111 |
| **Italy** | 2,193 | high | 4.4 | 1.6 | 7.3 | 68 | 25 | 112 |
| **Montenegro** | 6,188 | high | 1.3 | 1.1 | 1.6 | 56 | 48 | 69 |
| **Russian Federation** | 1,219 | high | 4.1 | 1.2 | 5.6 | 35 | 10 | 48 |
| **Egypt** | 244 | high | 15.7 | 13.9 | 17.5 | 27 | 24 | 30 |
| **Switzerland** | 2,326 | high | 1.5 | 0.7 | 1.8 | 24 | 11 | 29 |
| **Ukraine** | 719 | high | 3.6 | 0.9 | 4.5 | 18 | 5 | 23 |
| **Australia** | 1,364 | high | 1.7 | 1.2 | 2.3 | 16 | 11 | 22 |
| **Romania** | 484 | high | 3.2 | 2.9 | 3.6 | 11 | 10 | 12 |
| **United States** | 1,181 | high | 1.3 | 1.2 | 2.4 | 11 | 10 | 20 |
| **Canada** | 824 | high | 1.1 | 0.6 | 1.3 | 6 | 3 | 7 |
| **Slovakia** | 614 | high | 1.4 | 0.9 | 2.0 | 6 | 4 | 9 |
| **Poland** | 703 | high | 1.1 | 0.6 | 1.9 | 5 | 3 | 9 |
| **China** | 434 | high | 1.3 | 0.4 | 2.0 | 4 | 1 | 6 |
| **Albania** | 169 | high | 2.4 | 2.0 | 2.8 | 3 | 2 | 3 |
| **Moldova** | 109 | high | 3.3 | 1.6 | 4.5 | 3 | 1 | 3 |
| **Bulgaria** | 284 | high | 1.1 | 0.3 | 2.4 | 2 | 1 | 5 |
| **South Africa** | 179 | high | 1.7 | 1.0 | 2.5 | 2 | 1 | 3 |
| **Brazil** | 184 | high | 1.6 | 1.1 | 1.6 | 2 | 1 | 2 |
| **Argentina** | 190 | high | 1.5 | 0.5 | 2.5 | 2 | 1 | 3 |
| **Iraq** | 82 | high | 3.2 | 0.3 | 3.2 | 2 | 0 | 2 |
| **Greece** | 133 | high | 1.9 | 0.5 | 2.6 | 2 | 0 | 2 |
| **Kazakhstan** | 64 | high | 3.3 | 1.0 | 6.7 | 1 | 0 | 3 |
| **Spain** | 117 | high | 1.7 | 0.4 | 2.6 | 1 | 0 | 2 |
| **Lebanon** | 59 | high | 3.1 | 2.5 | 3.9 | 1 | 1 | 2 |
| **Libya** | 145 | high | 1.2 | 1.2 | 2.3 | 1 | 1 | 2 |
| **New Zealand** | 89 | high | 1.9 | 0.8 | 2.2 | 1 | 0 | 1 |
| **Israel** | 82 | high | 2.0 | 0.9 | 2.0 | 1 | 1 | 1 |
| **Chile** | 106 | high | 1.2 | 0.5 | 2.1 | 1 | 0 | 2 |
| **Belarus** | 85 | high | 1.3 | 0.9 | 2.9 | 1 | 1 | 2 |
| **Peru** | 80 | high | 1.2 | 0.4 | 1.6 | 1 | 0 | 1 |
| **Algeria** | 57 | high | 1.4 | 0.2 | 2.5 | 1 | 0 | 1 |
| **Turkey** | 63 | high | 1.0 | 0.7 | 1.1 | 0 | 0 | 0 |
| **Germany** | 29,327 | low | 0.5 | 0.3 | 0.9 | 103 | 62 | 185 |
| **Austria** | 4,194 | low | 0.5 | 0.1 | 0.7 | 15 | 3 | 21 |
| **France** | 1,622 | low | 0.7 | 0.5 | 0.8 | 8 | 6 | 9 |
| **Hungary** | 904 | low | 0.8 | 0.4 | 2.7 | 5 | 3 | 17 |
| **Czech Republic** | 846 | low | 0.7 | 0.2 | 0.7 | 4 | 1 | 4 |
| **Sweden** | 538 | low | 0.6 | 0.5 | 0.7 | 2 | 2 | 3 |
| **Belgium** | 296 | low | 0.9 | 0.1 | 1.2 | 2 | 0 | 2 |
| **United Kingdom** | 443 | low | 0.6 | 0.4 | 1.2 | 2 | 1 | 4 |
| **Netherlands** | 667 | low | 0.2 | 0.1 | 0.4 | 1 | 0 | 2 |
| **Philippines** | 67 | low | 0.9 | 0.3 | 2.0 | 0 | 0 | 1 |
| **Denmark** | 80 | low | 0.7 | 0.5 | 0.7 | 0 | 0 | 0 |

## Cyprus

| **Country of birth** | **Population (>15 yrs)** | **Anti-HCV endemicity** | **Anti-HCV prevalence** | | | **Estimated no. of CHC cases** | | |
| --- | --- | --- | --- | --- | --- | --- | --- | --- |
|  |  |  | **%** | **Lower limit** | **Upper limit** | **Cases** | **Lower** | **Upper** |
| **Georgia** | 11,511 | high | 6.7 | 5.6 | 7.3 | 540 | 451 | 588 |
| **Romania** | 22,475 | high | 3.2 | 2.9 | 3.6 | 503 | 456 | 566 |
| **Egypt** | 3,193 | high | 15.7 | 13.9 | 17.5 | 351 | 311 | 391 |
| **Russia** | 9,255 | high | 4.1 | 1.2 | 5.6 | 266 | 78 | 363 |
| **Greece** | 16,210 | high | 1.9 | 0.5 | 2.6 | 216 | 57 | 295 |
| **Bulgaria** | 17,320 | high | 1.1 | 0.3 | 2.4 | 133 | 36 | 291 |
| **Ukraine** | 3,484 | high | 3.6 | 0.9 | 4.5 | 88 | 22 | 110 |
| **Syria** | 2,991 | high | 3.1 | 2.5 | 3.9 | 65 | 52 | 82 |
| **Pakistan** | 1,520 | high | 5.0 | 4.4 | 5.5 | 53 | 47 | 59 |
| **Sri Lanka** | 7,298 | high | 1.0 | 0.8 | 1.8 | 51 | 41 | 92 |
| **Moldova** | 2,154 | high | 3.3 | 1.6 | 4.5 | 50 | 24 | 68 |
| **Vietnam** | 7,009 | high | 1.0 | 0.8 | 1.8 | 49 | 39 | 88 |
| **Armenia** | 1,086 | high | 5.4 | 3.5 | 6.8 | 41 | 27 | 52 |
| **Lebanon** | 1,489 | high | 3.1 | 2.5 | 3.9 | 32 | 26 | 41 |
| **Iraq** | 1,418 | high | 3.2 | 0.3 | 3.2 | 32 | 3 | 32 |
| **South Africa** | 2,338 | high | 1.7 | 1.0 | 2.5 | 28 | 16 | 41 |
| **Nigeria** | 405 | high | 8.4 | 3.9 | 12.8 | 24 | 11 | 36 |
| **Cameroon** | 279 | high | 11.6 | 4.3 | 29.7 | 23 | 8 | 58 |
| **Poland** | 2,708 | high | 1.1 | 0.6 | 1.9 | 21 | 11 | 36 |
| **Australia** | 1,477 | high | 1.7 | 1.2 | 2.3 | 18 | 12 | 24 |
| **Bangladesh** | 1,828 | high | 1.3 | 0.2 | 2.2 | 17 | 3 | 28 |
| **United States** | 1,664 | high | 1.3 | 1.2 | 2.4 | 15 | 14 | 28 |
| **Latvia** | 806 | high | 2.4 | 1.7 | 3.3 | 14 | 10 | 19 |
| **Lithuania** | 637 | high | 2.9 | 0.7 | 3.0 | 13 | 3 | 13 |
| **Palestinian Territory** | 586 | high | 3.1 | 2.5 | 3.9 | 13 | 10 | 16 |
| **Italy** | 362 | high | 4.4 | 1.6 | 7.3 | 11 | 4 | 18 |
| **China** | 1,079 | high | 1.3 | 0.4 | 2.0 | 10 | 3 | 15 |
| **Slovakia** | 924 | high | 1.4 | 0.9 | 2.0 | 9 | 6 | 13 |
| **Congo** | 296 | high | 4.2 | 2.4 | 9.2 | 9 | 5 | 19 |
| **Serbia** | 956 | high | 1.3 | 1.1 | 1.6 | 9 | 7 | 11 |
| **Jordan** | 335 | high | 3.1 | 2.5 | 3.9 | 7 | 6 | 9 |
| **Canada** | 737 | high | 1.1 | 0.6 | 1.3 | 6 | 3 | 7 |
| **Belarus** | 613 | high | 1.3 | 0.9 | 2.9 | 6 | 4 | 12 |
| **Albania** | 270 | high | 2.4 | 2.0 | 2.8 | 5 | 4 | 5 |
| **Ireland** | 502 | high | 1.1 | 0.7 | 1.6 | 4 | 2 | 6 |
| **Zimbabwe** | 330 | high | 1.6 | 1.0 | 9.1 | 4 | 2 | 21 |
| **Nepal** | 471 | high | 1.1 | 0.7 | 1.5 | 4 | 2 | 5 |
| **Turkey** | 411 | high | 1.0 | 0.7 | 1.1 | 3 | 2 | 3 |
| **Sudan** | 285 | high | 1.0 | 0.6 | 3.1 | 2 | 1 | 6 |
| **United Kingdom** | 29,727 | low | 0.6 | 0.4 | 1.2 | 125 | 83 | 250 |
| **Philippines** | 9,941 | low | 0.9 | 0.3 | 2.0 | 63 | 21 | 139 |
| **India** | 2,940 | low | 0.8 | 0.4 | 1.0 | 16 | 8 | 21 |
| **Germany** | 1,730 | low | 0.5 | 0.3 | 0.9 | 6 | 4 | 11 |
| **Iran** | 1,214 | low | 0.5 | 0.2 | 1.0 | 4 | 2 | 8 |
| **Hungary** | 587 | low | 0.8 | 0.4 | 2.7 | 3 | 2 | 11 |
| **France** | 562 | low | 0.7 | 0.5 | 0.8 | 3 | 2 | 3 |
| **Sweden** | 513 | low | 0.6 | 0.5 | 0.7 | 2 | 2 | 3 |
| **Czech Republic** | 433 | low | 0.7 | 0.2 | 0.7 | 2 | 1 | 2 |
| **Netherlands** | 344 | low | 0.2 | 0.1 | 0.4 | 0 | 0 | 1 |

## Czech Republic

| **Country of birth** | **Population (>15 yrs)** | **Anti-HCV endemicity** | **Anti-HCV prevalence** | | | **Estimated no. of CHC cases** | | |
| --- | --- | --- | --- | --- | --- | --- | --- | --- |
|  |  |  | **%** | **Lower limit** | **Upper limit** | **Cases** | **Lower** | **Upper** |
| **Ukraine** | 95,966 | high | 3.6 | 0.9 | 4.5 | 2,418 | 605 | 3,023 |
| **Russia** | 26,943 | high | 4.1 | 1.2 | 5.6 | 773 | 226 | 1,056 |
| **Slovakia** | 77,821 | high | 1.4 | 0.9 | 2 | 763 | 490 | 1,089 |
| **Vietnam** | 45,167 | high | 1 | 0.8 | 1.8 | 316 | 253 | 569 |
| **Mongolia** | 4,041 | high | 10.8 | 8.7 | 15.6 | 305 | 246 | 441 |
| **Uzbekistan** | 1,908 | high | 11.3 | 6.4 | 13.1 | 151 | 85 | 175 |
| **Poland** | 18,032 | high | 1.1 | 0.6 | 1.9 | 139 | 76 | 240 |
| **Moldova** | 5,969 | high | 3.3 | 1.6 | 4.5 | 138 | 67 | 188 |
| **Kazakhstan** | 5,416 | high | 3.3 | 1 | 6.7 | 125 | 38 | 254 |
| **Romania** | 4,877 | high | 3.2 | 2.9 | 3.6 | 109 | 99 | 123 |
| **Italy** | 2,757 | high | 4.4 | 1.6 | 7.3 | 85 | 31 | 141 |
| **Armenia** | 1,854 | high | 5.4 | 3.5 | 6.8 | 70 | 45 | 88 |
| **Egypt** | 585 | high | 15.7 | 13.9 | 17.5 | 64 | 57 | 72 |
| **Bulgaria** | 7,464 | high | 1.1 | 0.3 | 2.4 | 57 | 16 | 125 |
| **Georgia** | 940 | high | 6.7 | 5.6 | 7.3 | 44 | 37 | 48 |
| **China** | 4,383 | high | 1.3 | 0.4 | 2 | 40 | 12 | 61 |
| **Belarus** | 3,715 | high | 1.3 | 0.9 | 2.9 | 34 | 23 | 75 |
| **Nigeria** | 563 | high | 8.4 | 3.9 | 12.8 | 33 | 15 | 50 |
| **United States** | 3,577 | high | 1.3 | 1.2 | 2.4 | 33 | 30 | 60 |
| **Bosnia and Herzegovina** | 2,442 | high | 1.3 | 1.1 | 1.6 | 22 | 19 | 27 |
| **Azerbaijan** | 900 | high | 3.1 | 1 | 6.7 | 20 | 6 | 42 |
| **Former Yugoslavia** | 2,038 | high | 1.3 | 1.1 | 1.6 | 19 | 16 | 23 |
| **Pakistan** | 430 | high | 5 | 4.4 | 5.5 | 15 | 13 | 17 |
| **FYR Macedonia** | 1,640 | high | 1.3 | 1.1 | 1.6 | 15 | 13 | 18 |
| **Syria** | 619 | high | 3.1 | 2.5 | 3.9 | 13 | 11 | 17 |
| **Thailand** | 653 | high | 2.7 | 1.8 | 3.7 | 12 | 8 | 17 |
| **Croatia** | 1,344 | high | 1.3 | 1.1 | 1.6 | 12 | 10 | 15 |
| **Kyrgyzstan** | 696 | high | 2.5 | 1.6 | 6.7 | 12 | 8 | 33 |
| **Japan** | 1,086 | high | 1.5 | 0.5 | 2.2 | 11 | 4 | 17 |
| **Lithuania** | 535 | high | 2.9 | 0.7 | 3 | 11 | 3 | 11 |
| **Iraq** | 452 | high | 3.2 | 0.3 | 3.2 | 10 | 1 | 10 |
| **Serbia** | 1,003 | high | 1.3 | 1.1 | 1.6 | 9 | 8 | 11 |
| **Spain** | 716 | high | 1.7 | 0.4 | 2.6 | 9 | 2 | 13 |
| **Greece** | 577 | high | 1.9 | 0.5 | 2.6 | 8 | 2 | 11 |
| **Turkey** | 1,070 | high | 1 | 0.7 | 1.1 | 7 | 5 | 8 |
| **Former Yugoslav Republic** | 767 | high | 1.3 | 1.1 | 1.6 | 7 | 6 | 9 |
| **Algeria** | 654 | high | 1.4 | 0.2 | 2.5 | 6 | 1 | 11 |
| **Tunisia** | 683 | high | 1.3 | 0.3 | 2.5 | 6 | 1 | 12 |
| **Switzerland** | 453 | high | 1.5 | 0.7 | 1.8 | 5 | 2 | 6 |
| **Canada** | 588 | high | 1.1 | 0.6 | 1.3 | 5 | 2 | 5 |
| **Ireland** | 516 | high | 1.1 | 0.7 | 1.6 | 4 | 3 | 6 |
| **Germany** | 13,333 | low | 0.5 | 0.3 | 0.9 | 47 | 28 | 84 |
| **United Kingdom** | 4,367 | low | 0.6 | 0.4 | 1.2 | 18 | 12 | 37 |
| **France** | 2,362 | low | 0.7 | 0.5 | 0.8 | 12 | 8 | 13 |
| **Austria** | 2,450 | low | 0.5 | 0.1 | 0.7 | 9 | 2 | 12 |
| **India** | 1,122 | low | 0.8 | 0.4 | 1 | 6 | 3 | 8 |
| **Hungary** | 952 | low | 0.8 | 0.4 | 2.7 | 5 | 3 | 18 |
| **South Korea** | 888 | low | 0.8 | 0.2 | 2.1 | 5 | 1 | 13 |
| **Netherlands** | 2,358 | low | 0.2 | 0.1 | 0.4 | 3 | 2 | 7 |
| **Belgium** | 498 | low | 0.9 | 0.1 | 1.2 | 3 | 0 | 4 |

## Denmark

| **Country of birth** | **Population (>15 yrs)** | **Anti-HCV endemicity** | **Anti-HCV prevalence** | | | **Estimated no. of CHC cases** | | |
| --- | --- | --- | --- | --- | --- | --- | --- | --- |
|  |  |  | **%** | **Lower limit** | **Upper limit** | **Cases** | **Lower** | **Upper** |
| **Iraq** | 20,222 | high | 3.2 | 0.3 | 3.2 | 453 | 42 | 453 |
| **Pakistan** | 11,658 | high | 5 | 4.4 | 5.5 | 408 | 359 | 449 |
| **Romania** | 12,370 | high | 3.2 | 2.9 | 3.6 | 277 | 251 | 312 |
| **Lebanon** | 11,975 | high | 3.1 | 2.5 | 3.9 | 260 | 210 | 327 |
| **Turkey** | 31,611 | high | 1 | 0.7 | 1.1 | 221 | 155 | 243 |
| **Poland** | 28,029 | high | 1.1 | 0.6 | 1.9 | 216 | 118 | 373 |
| **Thailand** | 10,371 | high | 2.7 | 1.8 | 3.7 | 196 | 131 | 269 |
| **Italy** | 5,580 | high | 4.4 | 1.6 | 7.3 | 172 | 62 | 285 |
| **Bosnia and Herzegovina** | 17,358 | high | 1.3 | 1.1 | 1.6 | 158 | 134 | 194 |
| **Lithuania** | 7,690 | high | 2.9 | 0.7 | 3 | 156 | 38 | 161 |
| **Ukraine** | 6,112 | high | 3.6 | 0.9 | 4.5 | 154 | 39 | 193 |
| **Russia** | 4,898 | high | 4.1 | 1.2 | 5.6 | 141 | 41 | 192 |
| **United States** | 9,865 | high | 1.3 | 1.2 | 2.4 | 90 | 83 | 166 |
| **Former Yugoslavia** | 9,675 | high | 1.3 | 1.1 | 1.6 | 88 | 74 | 108 |
| **Afghanistan** | 10,579 | high | 1.1 | 0.6 | 1.9 | 81 | 44 | 141 |
| **China** | 8,913 | high | 1.3 | 0.4 | 2 | 81 | 25 | 125 |
| **Syria** | 3,279 | high | 3.1 | 2.5 | 3.9 | 71 | 57 | 90 |
| **Somalia** | 9,621 | high | 1 | 0.6 | 3.1 | 67 | 40 | 209 |
| **Latvia** | 3,747 | high | 2.4 | 1.7 | 3.3 | 63 | 45 | 87 |
| **Vietnam** | 8,992 | high | 1 | 0.8 | 1.8 | 63 | 50 | 113 |
| **Ghana** | 1,682 | high | 5.3 | 2.9 | 9.1 | 62 | 34 | 107 |
| **Spain** | 4,979 | high | 1.7 | 0.4 | 2.6 | 59 | 14 | 91 |
| **Morocco** | 5,168 | high | 1.6 | 0.6 | 1.9 | 58 | 22 | 69 |
| **Sri Lanka** | 7,616 | high | 1 | 0.8 | 1.8 | 53 | 43 | 96 |
| **Bulgaria** | 4,900 | high | 1.1 | 0.3 | 2.4 | 38 | 10 | 82 |
| **Brazil** | 2,918 | high | 1.6 | 1.1 | 1.6 | 33 | 22 | 33 |
| **Australia** | 2,533 | high | 1.7 | 1.2 | 2.3 | 30 | 21 | 41 |
| **Canada** | 3,352 | high | 1.1 | 0.6 | 1.3 | 26 | 14 | 31 |
| **Greece** | 1,864 | high | 1.9 | 0.5 | 2.6 | 25 | 7 | 34 |
| **Macedonia** | 2,700 | high | 1.3 | 1.1 | 1.6 | 25 | 21 | 30 |
| **Switzerland** | 2,115 | high | 1.5 | 0.7 | 1.8 | 22 | 10 | 27 |
| **Japan** | 1,671 | high | 1.5 | 0.5 | 2.2 | 18 | 6 | 26 |
| **Colombia** | 2,312 | high | 1 | 0.8 | 1.4 | 16 | 13 | 23 |
| **Nepal** | 1,686 | high | 1.1 | 0.7 | 1.5 | 13 | 8 | 18 |
| **Germany** | 32,196 | low | 0.5 | 0.3 | 0.9 | 113 | 68 | 203 |
| **Greenland (DK)** | 13,948 | low | 0.9 | 0.7 | 1.5 | 88 | 68 | 146 |
| **Norway** | 17,777 | low | 0.7 | 0.6 | 0.9 | 87 | 75 | 112 |
| **Sweden** | 19,309 | low | 0.6 | 0.5 | 0.7 | 81 | 68 | 95 |
| **Faeroe Islands (DK)** | 10,733 | low | 0.9 | 0.7 | 1.5 | 68 | 53 | 113 |
| **United Kingdom** | 15,292 | low | 0.6 | 0.4 | 1.2 | 64 | 43 | 128 |
| **Philippines** | 9,706 | low | 0.9 | 0.3 | 2 | 61 | 20 | 136 |
| **Iran** | 12,847 | low | 0.5 | 0.2 | 1 | 45 | 18 | 90 |
| **South Korea** | 7,997 | low | 0.8 | 0.2 | 2.1 | 45 | 11 | 118 |
| **Iceland** | 6,871 | low | 0.9 | 0.7 | 1.5 | 43 | 34 | 72 |
| **India** | 7,681 | low | 0.8 | 0.4 | 1 | 43 | 22 | 54 |
| **France** | 6,032 | low | 0.7 | 0.5 | 0.8 | 30 | 21 | 34 |
| **Hungary** | 3,487 | low | 0.8 | 0.4 | 2.7 | 20 | 10 | 66 |
| **Finland** | 3,429 | low | 0.7 | 0.6 | 0.9 | 17 | 14 | 22 |
| **Netherlands** | 6,088 | low | 0.2 | 0.1 | 0.4 | 9 | 4 | 17 |

## Estonia

| **Country of birth** | **Population (>15 yrs)** | **Anti-HCV endemicity** | **Anti-HCV prevalence** | | | **Estimated no. of CHC cases** | | |
| --- | --- | --- | --- | --- | --- | --- | --- | --- |
|  |  |  | **%** | **Lower limit** | **Upper limit** | **Cases** | **Lower** | **Upper** |
| **Russia** | 137,646 | high | 4.1 | 1.2 | 5.6 | 3,950 | 1,156 | 5,396 |
| **Ukraine** | 21,598 | high | 3.6 | 0.9 | 4.5 | 544 | 136 | 680 |
| **Belarus** | 11,587 | high | 1.3 | 0.9 | 2.9 | 105 | 73 | 235 |
| **Kazakhstan** | 3,790 | high | 3.3 | 1 | 6.7 | 88 | 27 | 178 |
| **Uzbekistan** | 1,088 | high | 11.3 | 6.4 | 13.1 | 86 | 49 | 100 |
| **Georgia** | 1,515 | high | 6.7 | 5.6 | 7.3 | 71 | 59 | 77 |
| **Latvia** | 3,971 | high | 2.4 | 1.7 | 3.3 | 67 | 47 | 92 |
| **Lithuania** | 1,844 | high | 2.9 | 0.7 | 3 | 37 | 9 | 39 |
| **Azerbaijan** | 1,494 | high | 3.1 | 1 | 6.7 | 32 | 10 | 70 |
| **Armenia** | 658 | high | 5.4 | 3.5 | 6.8 | 25 | 16 | 31 |
| **Moldova** | 835 | high | 3.3 | 1.6 | 4.5 | 19 | 9 | 26 |
| **Turkmenistan** | 340 | high | 5.6 | 1.1 | 6.7 | 13 | 3 | 16 |
| **Kyrgyzstan** | 547 | high | 2.5 | 1.6 | 6.7 | 10 | 6 | 26 |
| **Tajikistan** | 367 | high | 3.1 | 1.1 | 6.7 | 8 | 3 | 17 |
| **Italy** | 192 | high | 4.4 | 1.6 | 7.3 | 6 | 2 | 10 |
| **Egypt** | 44 | high | 15.7 | 13.9 | 17.5 | 5 | 4 | 5 |
| **Poland** | 543 | high | 1.1 | 0.6 | 1.9 | 4 | 2 | 7 |
| **Mongolia** | 50 | high | 10.8 | 8.7 | 15.6 | 4 | 3 | 5 |
| **United States** | 361 | high | 1.3 | 1.2 | 2.4 | 3 | 3 | 6 |
| **China** | 216 | high | 1.3 | 0.4 | 2 | 2 | 1 | 3 |
| **Romania** | 61 | high | 3.2 | 2.9 | 3.6 | 1 | 1 | 2 |
| **Pakistan** | 37 | high | 5 | 4.4 | 5.5 | 1 | 1 | 1 |
| **Spain** | 99 | high | 1.7 | 0.4 | 2.6 | 1 | 0 | 2 |
| **Turkey** | 108 | high | 1 | 0.7 | 1.1 | 1 | 1 | 1 |
| **Brazil** | 62 | high | 1.6 | 1.1 | 1.6 | 1 | 0 | 1 |
| **Canada** | 89 | high | 1.1 | 0.6 | 1.3 | 1 | 0 | 1 |
| **Bulgaria** | 86 | high | 1.1 | 0.3 | 2.4 | 1 | 0 | 1 |
| **Australia** | 55 | high | 1.7 | 1.2 | 2.3 | 1 | 0 | 1 |
| **Thailand** | 29 | high | 2.7 | 1.8 | 3.7 | 1 | 0 | 1 |
| **Japan** | 48 | high | 1.5 | 0.5 | 2.2 | 1 | 0 | 1 |
| **Israel** | 27 | high | 2 | 0.9 | 2 | 0 | 0 | 0 |
| **Portugal** | 26 | high | 1.8 | 0.5 | 2.9 | 0 | 0 | 1 |
| **Mexico** | 30 | high | 1.4 | 1.1 | 1.6 | 0 | 0 | 0 |
| **Switzerland** | 25 | high | 1.5 | 0.7 | 1.8 | 0 | 0 | 0 |
| **Ireland** | 27 | high | 1.1 | 0.7 | 1.6 | 0 | 0 | 0 |
| **Finland** | 2,075 | low | 0.7 | 0.6 | 0.9 | 10 | 9 | 13 |
| **Germany** | 1,153 | low | 0.5 | 0.3 | 0.9 | 4 | 2 | 7 |
| **Sweden** | 366 | low | 0.6 | 0.5 | 0.7 | 2 | 1 | 2 |
| **United Kingdom** | 310 | low | 0.6 | 0.4 | 1.2 | 1 | 1 | 3 |
| **India** | 160 | low | 0.8 | 0.4 | 1 | 1 | 0 | 1 |
| **France** | 173 | low | 0.7 | 0.5 | 0.8 | 1 | 1 | 1 |
| **Hungary** | 101 | low | 0.8 | 0.4 | 2.7 | 1 | 0 | 2 |
| **Denmark** | 104 | low | 0.7 | 0.5 | 0.7 | 1 | 0 | 1 |
| **Norway** | 84 | low | 0.7 | 0.6 | 0.9 | 0 | 0 | 1 |
| **Czech Republic** | 56 | low | 0.7 | 0.2 | 0.7 | 0 | 0 | 0 |
| **Belgium** | 37 | low | 0.9 | 0.1 | 1.2 | 0 | 0 | 0 |
| **Austria** | 48 | low | 0.5 | 0.1 | 0.7 | 0 | 0 | 0 |
| **Netherlands** | 90 | low | 0.2 | 0.1 | 0.4 | 0 | 0 | 0 |
| **Iran** | 34 | low | 0.5 | 0.2 | 1 | 0 | 0 | 0 |

## Finland

| **Country of birth** | **Population (>15 yrs)** | **Anti-HCV endemicity** | **Anti-HCV prevalence** | | | **Estimated no. of CHC cases** | | |
| --- | --- | --- | --- | --- | --- | --- | --- | --- |
|  |  |  | **%** | **Lower limit** | **Upper limit** | **Cases** | **Lower** | **Upper** |
| **Former Soviet Union** | 52,318 | high | 3.3 | 1.6 | 4.5 | 1,209 | 586 | 1,648 |
| **Estonia** | 30,299 | high | 3.3 | 1.6 | 4.5 | 700 | 339 | 954 |
| **Russia** | 6,562 | high | 4.1 | 1.2 | 5.6 | 188 | 55 | 257 |
| **Iraq** | 7,759 | high | 3.2 | 0.3 | 3.2 | 174 | 16 | 174 |
| **Thailand** | 6,434 | high | 2.7 | 1.8 | 3.7 | 122 | 81 | 167 |
| **Nigeria** | 1,727 | high | 8.4 | 3.9 | 12.8 | 102 | 47 | 155 |
| **Egypt** | 814 | high | 15.7 | 13.9 | 17.5 | 89 | 79 | 100 |
| **China** | 7,174 | high | 1.3 | 0.4 | 2 | 65 | 20 | 100 |
| **Former Yugoslavia** | 6,481 | high | 1.3 | 1.1 | 1.6 | 59 | 50 | 73 |
| **Italy** | 1,877 | high | 4.4 | 1.6 | 7.3 | 58 | 21 | 96 |
| **Pakistan** | 1,622 | high | 5 | 4.4 | 5.5 | 57 | 50 | 62 |
| **Somalia** | 8,103 | high | 1 | 0.6 | 3.1 | 57 | 34 | 176 |
| **Romania** | 1,960 | high | 3.2 | 2.9 | 3.6 | 44 | 40 | 49 |
| **Ghana** | 1,075 | high | 5.3 | 2.9 | 9.1 | 40 | 22 | 68 |
| **Turkey** | 5,395 | high | 1 | 0.7 | 1.1 | 38 | 26 | 42 |
| **Vietnam** | 5,000 | high | 1 | 0.8 | 1.8 | 35 | 28 | 63 |
| **DR Congo** | 1,159 | high | 4.3 | 3.2 | 13.7 | 35 | 26 | 111 |
| **United States** | 3,573 | high | 1.3 | 1.2 | 2.4 | 33 | 30 | 60 |
| **Ukraine** | 1,055 | high | 3.6 | 0.9 | 4.5 | 27 | 7 | 33 |
| **Poland** | 3,105 | high | 1.1 | 0.6 | 1.9 | 24 | 13 | 41 |
| **Afghanistan** | 3,038 | high | 1.1 | 0.6 | 1.9 | 23 | 13 | 40 |
| **Spain** | 1,760 | high | 1.7 | 0.4 | 2.6 | 21 | 5 | 32 |
| **Latvia** | 1,198 | high | 2.4 | 1.7 | 3.3 | 20 | 14 | 28 |
| **Morocco** | 1,788 | high | 1.6 | 0.6 | 1.9 | 20 | 8 | 24 |
| **Bangladesh** | 1,584 | high | 1.3 | 0.2 | 2.2 | 14 | 2 | 24 |
| **Ethiopia** | 1,473 | high | 1.3 | 0.7 | 5.8 | 13 | 7 | 60 |
| **Nepal** | 1,675 | high | 1.1 | 0.7 | 1.5 | 13 | 8 | 18 |
| **Myanmar (Burma)** | 1,041 | high | 1.7 | 1 | 2.7 | 12 | 7 | 20 |
| **Japan** | 1,142 | high | 1.5 | 0.5 | 2.2 | 12 | 4 | 18 |
| **Brazil** | 1,012 | high | 1.6 | 1.1 | 1.6 | 11 | 8 | 11 |
| **Greece** | 809 | high | 1.9 | 0.5 | 2.6 | 11 | 3 | 15 |
| **Bulgaria** | 1,392 | high | 1.1 | 0.3 | 2.4 | 11 | 3 | 23 |
| **Canada** | 1,391 | high | 1.1 | 0.6 | 1.3 | 11 | 6 | 13 |
| **Australia** | 876 | high | 1.7 | 1.2 | 2.3 | 10 | 7 | 14 |
| **Switzerland** | 802 | high | 1.5 | 0.7 | 1.8 | 8 | 4 | 10 |
| **Kenya** | 977 | high | 1 | 0.6 | 3.1 | 7 | 4 | 21 |
| **Sri Lanka** | 787 | high | 1 | 0.8 | 1.8 | 6 | 4 | 10 |
| **Sudan** | 743 | high | 1 | 0.6 | 3.1 | 5 | 3 | 16 |
| **Sweden** | 29,695 | low | 0.6 | 0.5 | 0.7 | 125 | 104 | 146 |
| **India** | 4,087 | low | 0.8 | 0.4 | 1 | 23 | 11 | 29 |
| **Germany** | 5,540 | low | 0.5 | 0.3 | 0.9 | 19 | 12 | 35 |
| **United Kingdom** | 4,226 | low | 0.6 | 0.4 | 1.2 | 18 | 12 | 35 |
| **Iran** | 4,490 | low | 0.5 | 0.2 | 1 | 16 | 6 | 31 |
| **Philippines** | 2,070 | low | 0.9 | 0.3 | 2 | 13 | 4 | 29 |
| **Hungary** | 1,727 | low | 0.8 | 0.4 | 2.7 | 10 | 5 | 33 |
| **France** | 1,901 | low | 0.7 | 0.5 | 0.8 | 9 | 7 | 11 |
| **Norway** | 1,007 | low | 0.7 | 0.6 | 0.9 | 5 | 4 | 6 |
| **Denmark** | 753 | low | 0.7 | 0.5 | 0.7 | 4 | 3 | 4 |
| **Netherlands** | 1,168 | low | 0.2 | 0.1 | 0.4 | 2 | 1 | 3 |

## France

| **Country of birth** | **Population (>15 yrs)** | **Anti-HCV endemicity** | **Anti-HCV prevalence** | | | **Estimated no. of CHC cases** | | |
| --- | --- | --- | --- | --- | --- | --- | --- | --- |
|  |  |  | **%** | **Lower limit** | **Upper limit** | **Cases** | **Lower** | **Upper** |
| **Algeria** | 1,331,679 | high | 1.4 | 0.2 | 2.5 | 13,050 | 1,864 | 23,304 |
| **Italy** | 337,920 | high | 4.4 | 1.6 | 7.3 | 10,408 | 3,785 | 17,268 |
| **Morocco** | 869,903 | high | 1.6 | 0.6 | 1.9 | 9,743 | 3,654 | 11,570 |
| **Portugal** | 595,315 | high | 1.8 | 0.5 | 2.9 | 7,501 | 2,084 | 12,085 |
| **Cameroon** | 73,384 | high | 11.6 | 4.3 | 29.7 | 5,959 | 2,209 | 15,257 |
| **Senegal** | 108,409 | high | 5.3 | 2.9 | 9.1 | 4,022 | 2,201 | 6,906 |
| **Tunisia** | 367,692 | high | 1.3 | 0.3 | 2.5 | 3,346 | 772 | 6,435 |
| **Spain** | 279,269 | high | 1.7 | 0.4 | 2.6 | 3,323 | 782 | 5,083 |
| **Egypt** | 28,413 | high | 15.7 | 13.9 | 17.5 | 3,123 | 2,765 | 3,481 |
| **Ivory Coast** | 83,132 | high | 5.3 | 2.9 | 9.1 | 3,084 | 1,688 | 5,296 |
| **Mali** | 64,198 | high | 5.3 | 2.9 | 9.1 | 2,382 | 1,303 | 4,089 |
| **Congo** | 62,883 | high | 4.2 | 2.4 | 9.2 | 1,849 | 1,056 | 4,050 |
| **DR Congo** | 59,712 | high | 4.3 | 3.2 | 13.7 | 1,797 | 1,338 | 5,726 |
| **Turkey** | 244,660 | high | 1 | 0.7 | 1.1 | 1,713 | 1,199 | 1,884 |
| **Romania** | 64,532 | high | 3.2 | 2.9 | 3.6 | 1,446 | 1,310 | 1,626 |
| **Gabon** | 17,862 | high | 11.2 | 2.1 | 20.7 | 1,400 | 263 | 2,588 |
| **Russia** | 41,390 | high | 4.1 | 1.2 | 5.6 | 1,188 | 348 | 1,622 |
| **Guinea** | 27,921 | high | 5.3 | 2.9 | 9.1 | 1,036 | 567 | 1,779 |
| **Cambodia** | 58,852 | high | 2.3 | 2.3 | 14.7 | 948 | 948 | 6,056 |
| **Lebanon** | 43,069 | high | 3.1 | 2.5 | 3.9 | 935 | 754 | 1,176 |
| **Madagascar** | 107,934 | high | 1.2 | 0.8 | 1.7 | 907 | 604 | 1,284 |
| **China** | 90,269 | high | 1.3 | 0.4 | 2 | 821 | 253 | 1,264 |
| **Togo** | 21,432 | high | 5.3 | 2.9 | 9.1 | 795 | 435 | 1,365 |
| **Switzerland** | 74,956 | high | 1.5 | 0.7 | 1.8 | 787 | 367 | 944 |
| **Cape Verde** | 21,049 | high | 5.3 | 2.9 | 9.1 | 781 | 427 | 1,341 |
| **Vietnam** | 110,495 | high | 1 | 0.8 | 1.8 | 773 | 619 | 1,392 |
| **Poland** | 98,983 | high | 1.1 | 0.6 | 1.9 | 762 | 416 | 1,316 |
| **Armenia** | 18,061 | high | 5.4 | 3.5 | 6.8 | 683 | 442 | 860 |
| **Pakistan** | 18,125 | high | 5 | 4.4 | 5.5 | 634 | 558 | 698 |
| **Serbia** | 66,929 | high | 1.3 | 1.1 | 1.6 | 609 | 515 | 750 |
| **Brazil** | 50,981 | high | 1.6 | 1.1 | 1.6 | 571 | 393 | 571 |
| **Benin** | 18,476 | high | 3.6 | 3.6 | 12.8 | 466 | 466 | 1,655 |
| **United States** | 41,040 | high | 1.3 | 1.2 | 2.4 | 373 | 345 | 689 |
| **Mauritius** | 40,769 | high | 1 | 0.8 | 1.8 | 285 | 228 | 514 |
| **Laos** | 40,379 | high | 1 | 0.8 | 1.8 | 283 | 226 | 509 |
| **Sri Lanka** | 38,458 | high | 1 | 0.8 | 1.8 | 269 | 215 | 485 |
| **Comoros** | 34,305 | high | 1 | 0.6 | 3.1 | 240 | 144 | 744 |
| **Japan** | 18,148 | high | 1.5 | 0.5 | 2.2 | 191 | 64 | 279 |
| **Canada** | 21,269 | high | 1.1 | 0.6 | 1.3 | 164 | 89 | 194 |
| **Colombia** | 21,823 | high | 1 | 0.8 | 1.4 | 153 | 122 | 214 |
| **Belgium** | 133,944 | low | 0.9 | 0.1 | 1.2 | 844 | 94 | 1,125 |
| **Germany** | 206,221 | low | 0.5 | 0.3 | 0.9 | 722 | 433 | 1,299 |
| **United Kingdom** | 151,328 | low | 0.6 | 0.4 | 1.2 | 636 | 424 | 1,271 |
| **Haiti** | 65,340 | low | 0.8 | 0.2 | 1.3 | 366 | 91 | 595 |
| **India** | 44,506 | low | 0.8 | 0.4 | 1 | 249 | 125 | 312 |
| **Suriname** | 20,675 | low | 0.8 | 0.2 | 1.3 | 116 | 29 | 188 |
| **Monaco** | 16,543 | low | 0.9 | 0.7 | 1.5 | 104 | 81 | 174 |
| **South Korea** | 18,411 | low | 0.8 | 0.2 | 2.1 | 103 | 26 | 271 |
| **Iran** | 21,617 | low | 0.5 | 0.2 | 1 | 76 | 30 | 151 |
| **Netherlands** | 36,707 | low | 0.2 | 0.1 | 0.4 | 51 | 26 | 103 |

## Germany

| **Country of birth** | **Population (>15 yrs)** | **Anti-HCV endemicity** | **Anti-HCV prevalence** | | | **Estimated no. of CHC cases** | | |
| --- | --- | --- | --- | --- | --- | --- | --- | --- |
|  |  |  | **%** | **Lower limit** | **Upper limit** | **Cases** | **Lower** | **Upper** |
| **Russia** | 943,240 | high | 4.1 | 1.2 | 5.6 | 27,071 | 7,923 | 36,975 |
| **Poland** | 2,721,070 | high | 1.1 | 0.6 | 1.9 | 20,952 | 11,428 | 36,190 |
| **Kazakhstan** | 780,840 | high | 3.3 | 1 | 6.7 | 18,037 | 5,466 | 36,621 |
| **Italy** | 323,210 | high | 4.4 | 1.6 | 7.3 | 9,955 | 3,620 | 16,516 |
| **Romania** | 442,280 | high | 3.2 | 2.9 | 3.6 | 9,907 | 8,978 | 11,145 |
| **Turkey** | 1,303,200 | high | 1 | 0.7 | 1.1 | 9,122 | 6,386 | 10,035 |
| **Ukraine** | 199,000 | high | 3.6 | 0.9 | 4.5 | 5,015 | 1,254 | 6,269 |
| **Uzbekistan** | 32,040 | high | 11.3 | 6.4 | 13.1 | 2,534 | 1,435 | 2,938 |
| **Greece** | 156,180 | high | 1.9 | 0.5 | 2.6 | 2,077 | 547 | 2,842 |
| **Iraq** | 75,660 | high | 3.2 | 0.3 | 3.2 | 1,695 | 159 | 1,695 |
| **Former Yugoslavia** | 173,100 | high | 1.3 | 1.1 | 1.6 | 1,575 | 1,333 | 1,939 |
| **Croatia** | 161,780 | high | 1.3 | 1.1 | 1.6 | 1,472 | 1,246 | 1,812 |
| **Bosnia and Herzegovina** | 157,600 | high | 1.3 | 1.1 | 1.6 | 1,434 | 1,214 | 1,765 |
| **Lebanon** | 60,900 | high | 3.1 | 2.5 | 3.9 | 1,322 | 1,066 | 1,663 |
| **Pakistan** | 37,300 | high | 5 | 4.4 | 5.5 | 1,306 | 1,149 | 1,436 |
| **Kosovo** | 129,000 | high | 1.3 | 1.1 | 1.6 | 1,174 | 993 | 1,445 |
| **Kyrgyzstan** | 66,310 | high | 2.5 | 1.6 | 6.7 | 1,160 | 743 | 3,110 |
| **Thailand** | 53,340 | high | 2.7 | 1.8 | 3.7 | 1,008 | 672 | 1,382 |
| **Morocco** | 89,850 | high | 1.6 | 0.6 | 1.9 | 1,006 | 377 | 1,195 |
| **Portugal** | 72,570 | high | 1.8 | 0.5 | 2.9 | 914 | 254 | 1,473 |
| **Ghana** | 22,750 | high | 5.3 | 2.9 | 9.1 | 844 | 462 | 1,449 |
| **Spain** | 69,920 | high | 1.7 | 0.4 | 2.6 | 832 | 196 | 1,273 |
| **Syria** | 37,480 | high | 3.1 | 2.5 | 3.9 | 813 | 656 | 1,023 |
| **United States** | 79,900 | high | 1.3 | 1.2 | 2.4 | 727 | 671 | 1,342 |
| **Lithuania** | 35,440 | high | 2.9 | 0.7 | 3 | 719 | 174 | 744 |
| **China** | 74,730 | high | 1.3 | 0.4 | 2 | 680 | 209 | 1,046 |
| **Switzerland** | 55,710 | high | 1.5 | 0.7 | 1.8 | 585 | 273 | 702 |
| **Vietnam** | 83,190 | high | 1 | 0.8 | 1.8 | 582 | 466 | 1,048 |
| **Afghanistan** | 73,210 | high | 1.1 | 0.6 | 1.9 | 564 | 307 | 974 |
| **Tajikistan** | 23,940 | high | 3.1 | 1.1 | 6.7 | 519 | 184 | 1,123 |
| **Bulgaria** | 65,940 | high | 1.1 | 0.3 | 2.4 | 508 | 138 | 1,108 |
| **Brazil** | 44,070 | high | 1.6 | 1.1 | 1.6 | 494 | 339 | 494 |
| **Macedonia** | 52,250 | high | 1.3 | 1.1 | 1.6 | 475 | 402 | 585 |
| **Serbia** | 39,300 | high | 1.3 | 1.1 | 1.6 | 358 | 303 | 440 |
| **Slovakia** | 31,360 | high | 1.4 | 0.9 | 2 | 307 | 198 | 439 |
| **Tunisia** | 32,660 | high | 1.3 | 0.3 | 2.5 | 297 | 69 | 572 |
| **Slovenia** | 29,660 | high | 1.3 | 1.1 | 1.6 | 270 | 228 | 332 |
| **Japan** | 24,260 | high | 1.5 | 0.5 | 2.2 | 255 | 85 | 374 |
| **Sri Lanka** | 34,470 | high | 1 | 0.8 | 1.8 | 241 | 193 | 434 |
| **Czech Republic** | 438,630 | low | 0.7 | 0.2 | 0.7 | 2,149 | 614 | 2,149 |
| **Austria** | 196,360 | low | 0.5 | 0.1 | 0.7 | 687 | 137 | 962 |
| **Hungary** | 122,660 | low | 0.8 | 0.4 | 2.7 | 687 | 343 | 2,318 |
| **France** | 101,680 | low | 0.7 | 0.5 | 0.8 | 498 | 356 | 569 |
| **Iran** | 99,990 | low | 0.5 | 0.2 | 1 | 350 | 140 | 700 |
| **United Kingdom** | 73,650 | low | 0.6 | 0.4 | 1.2 | 309 | 206 | 619 |
| **India** | 47,410 | low | 0.8 | 0.4 | 1 | 265 | 133 | 332 |
| **Philippines** | 38,050 | low | 0.9 | 0.3 | 2 | 240 | 80 | 533 |
| **Belgium** | 24,230 | low | 0.9 | 0.1 | 1.2 | 153 | 17 | 204 |
| **Netherlands** | 102,210 | low | 0.2 | 0.1 | 0.4 | 143 | 72 | 286 |
| **Czech Republic** | 24,400 | low | 0.7 | 0.2 | 0.7 | 120 | 34 | 120 |

## Greece

| **Country of birth** | **Population (>15 yrs)** | **Anti-HCV endemicity** | **Anti-HCV prevalence** | | | **Estimated no. of CHC cases** | | |
| --- | --- | --- | --- | --- | --- | --- | --- | --- |
|  |  |  | **%** | **Lower limit** | **Upper limit** | **Cases** | **Lower** | **Upper** |
| **Albania** | 323,392 | high | 2.4 | 2 | 2.8 | 5,433 | 4,527 | 6,338 |
| **Georgia** | 49,076 | high | 6.7 | 5.6 | 7.3 | 2,302 | 1,924 | 2,508 |
| **Egypt** | 10,304 | high | 15.7 | 13.9 | 17.5 | 1,132 | 1,003 | 1,262 |
| **Russia** | 34,197 | high | 4.1 | 1.2 | 5.6 | 981 | 287 | 1,341 |
| **Pakistan** | 21,769 | high | 5 | 4.4 | 5.5 | 762 | 670 | 838 |
| **Romania** | 29,629 | high | 3.2 | 2.9 | 3.6 | 664 | 601 | 747 |
| **Armenia** | 8,660 | high | 5.4 | 3.5 | 6.8 | 327 | 212 | 412 |
| **Ukraine** | 10,393 | high | 3.6 | 0.9 | 4.5 | 262 | 65 | 327 |
| **Bulgaria** | 31,730 | high | 1.1 | 0.3 | 2.4 | 244 | 67 | 533 |
| **Syria** | 9,089 | high | 3.1 | 2.5 | 3.9 | 197 | 159 | 248 |
| **Italy** | 3,102 | high | 4.4 | 1.6 | 7.3 | 96 | 35 | 158 |
| **Poland** | 8,505 | high | 1.1 | 0.6 | 1.9 | 65 | 36 | 113 |
| **Bangladesh** | 6,815 | high | 1.3 | 0.2 | 2.2 | 62 | 10 | 105 |
| **United States** | 6,682 | high | 1.3 | 1.2 | 2.4 | 61 | 56 | 112 |
| **Turkey** | 8,502 | high | 1 | 0.7 | 1.1 | 60 | 42 | 65 |
| **Moldova** | 1,616 | high | 3.3 | 1.6 | 4.5 | 37 | 18 | 51 |
| **Canada** | 4,804 | high | 1.1 | 0.6 | 1.3 | 37 | 20 | 44 |
| **Morocco** | 2,996 | high | 1.6 | 0.6 | 1.9 | 34 | 13 | 40 |
| **Australia** | 2,622 | high | 1.7 | 1.2 | 2.3 | 31 | 22 | 42 |
| **Iraq** | 1,039 | high | 3.2 | 0.3 | 3.2 | 23 | 2 | 23 |
| **South Africa** | 1,798 | high | 1.7 | 1 | 2.5 | 21 | 13 | 31 |
| **Serbia** | 2,168 | high | 1.3 | 1.1 | 1.6 | 20 | 17 | 24 |
| **Lithuania** | 731 | high | 2.9 | 0.7 | 3 | 15 | 4 | 15 |
| **China** | 1,433 | high | 1.3 | 0.4 | 2 | 13 | 4 | 20 |
| **Venezuela** | 1,050 | high | 1.5 | 0.3 | 2.6 | 11 | 2 | 19 |
| **Thailand** | 571 | high | 2.7 | 1.8 | 3.7 | 11 | 7 | 15 |
| **Lebanon** | 486 | high | 3.1 | 2.5 | 3.9 | 11 | 9 | 13 |
| **Switzerland** | 943 | high | 1.5 | 0.7 | 1.8 | 10 | 5 | 12 |
| **Macedonia** | 642 | high | 1.3 | 1.1 | 1.6 | 6 | 5 | 7 |
| **Israel** | 360 | high | 2 | 0.9 | 2 | 5 | 2 | 5 |
| **Brazil** | 448 | high | 1.6 | 1.1 | 1.6 | 5 | 3 | 5 |
| **Sri Lanka** | 708 | high | 1 | 0.8 | 1.8 | 5 | 4 | 9 |
| **Libya** | 572 | high | 1.2 | 1.2 | 2.3 | 5 | 5 | 9 |
| **Ireland** | 504 | high | 1.1 | 0.7 | 1.6 | 4 | 2 | 6 |
| **Sudan** | 547 | high | 1 | 0.6 | 3.1 | 4 | 2 | 12 |
| **Tunisia** | 395 | high | 1.3 | 0.3 | 2.5 | 4 | 1 | 7 |
| **Germany** | 19,193 | low | 0.5 | 0.3 | 0.9 | 67 | 40 | 121 |
| **Cyprus** | 9,288 | low | 0.6 | 0.5 | 1.9 | 39 | 33 | 124 |
| **Philippines** | 5,980 | low | 0.9 | 0.3 | 2 | 38 | 13 | 84 |
| **United Kingdom** | 7,938 | low | 0.6 | 0.4 | 1.2 | 33 | 22 | 67 |
| **India** | 3,679 | low | 0.8 | 0.4 | 1 | 21 | 10 | 26 |
| **France** | 3,287 | low | 0.7 | 0.5 | 0.8 | 16 | 12 | 18 |
| **Finland** | 1,707 | low | 0.7 | 0.6 | 0.9 | 8 | 7 | 11 |
| **Czech Republic** | 1,649 | low | 0.7 | 0.2 | 0.7 | 8 | 2 | 8 |
| **Indonesia** | 1,372 | low | 0.8 | 0.4 | 2 | 8 | 4 | 19 |
| **Belgium** | 1,142 | low | 0.9 | 0.1 | 1.2 | 7 | 1 | 10 |
| **Hungary** | 573 | low | 0.8 | 0.4 | 2.7 | 3 | 2 | 11 |
| **Sweden** | 626 | low | 0.6 | 0.5 | 0.7 | 3 | 2 | 3 |
| **Iran** | 733 | low | 0.5 | 0.2 | 1 | 3 | 1 | 5 |
| **Austria** | 677 | low | 0.5 | 0.1 | 0.7 | 2 | 0 | 3 |

## Hungary

| **Country of birth** | **Population (>15 yrs)** | **Anti-HCV endemicity** | **Anti-HCV prevalence** | | | **Estimated no. of CHC cases** | | |
| --- | --- | --- | --- | --- | --- | --- | --- | --- |
|  |  |  | **%** | **Lower limit** | **Upper limit** | **Cases** | **Lower** | **Upper** |
| **Romania** | 186,660 | high | 3.2 | 2.9 | 3.6 | 4,181 | 3,789 | 4,704 |
| **Ukraine** | 27,866 | high | 3.6 | 0.9 | 4.5 | 702 | 176 | 878 |
| **Former Soviet Union** | 14,061 | high | 3.3 | 1.6 | 4.5 | 325 | 157 | 443 |
| **Serbia** | 34,934 | high | 1.3 | 1.1 | 1.6 | 318 | 269 | 391 |
| **Slovakia** | 20,412 | high | 1.4 | 0.9 | 2 | 200 | 129 | 286 |
| **China** | 9,620 | high | 1.3 | 0.4 | 2 | 88 | 27 | 135 |
| **Russia** | 2,816 | high | 4.1 | 1.2 | 5.6 | 81 | 24 | 110 |
| **Italy** | 2,536 | high | 4.4 | 1.6 | 7.3 | 78 | 28 | 130 |
| **Egypt** | 697 | high | 15.7 | 13.9 | 17.5 | 77 | 68 | 85 |
| **Nigeria** | 1,277 | high | 8.4 | 3.9 | 12.8 | 75 | 35 | 114 |
| **Mongolia** | 980 | high | 10.8 | 8.7 | 15.6 | 74 | 60 | 107 |
| **Former Czechoslovakia** | 5,797 | high | 1.3 | 1.1 | 1.6 | 53 | 45 | 65 |
| **United States** | 4,214 | high | 1.3 | 1.2 | 2.4 | 38 | 35 | 71 |
| **Syria** | 1,060 | high | 3.1 | 2.5 | 3.9 | 23 | 19 | 29 |
| **Vietnam** | 3,110 | high | 1 | 0.8 | 1.8 | 22 | 17 | 39 |
| **Poland** | 2,635 | high | 1.1 | 0.6 | 1.9 | 20 | 11 | 35 |
| **Israel** | 1,266 | high | 2 | 0.9 | 2 | 18 | 8 | 18 |
| **Croatia** | 1,922 | high | 1.3 | 1.1 | 1.6 | 17 | 15 | 22 |
| **Iraq** | 767 | high | 3.2 | 0.3 | 3.2 | 17 | 2 | 17 |
| **Switzerland** | 1,373 | high | 1.5 | 0.7 | 1.8 | 14 | 7 | 17 |
| **Turkey** | 1,908 | high | 1 | 0.7 | 1.1 | 13 | 9 | 15 |
| **Thailand** | 676 | high | 2.7 | 1.8 | 3.7 | 13 | 9 | 18 |
| **Greece** | 950 | high | 1.9 | 0.5 | 2.6 | 13 | 3 | 17 |
| **Japan** | 1,026 | high | 1.5 | 0.5 | 2.2 | 11 | 4 | 16 |
| **Spain** | 870 | high | 1.7 | 0.4 | 2.6 | 10 | 2 | 16 |
| **Kazakhstan** | 440 | high | 3.3 | 1 | 6.7 | 10 | 3 | 21 |
| **Bulgaria** | 1,282 | high | 1.1 | 0.3 | 2.4 | 10 | 3 | 22 |
| **Canada** | 1,169 | high | 1.1 | 0.6 | 1.3 | 9 | 5 | 11 |
| **Afghanistan** | 1,083 | high | 1.1 | 0.6 | 1.9 | 8 | 5 | 14 |
| **Australia** | 668 | high | 1.7 | 1.2 | 2.3 | 8 | 6 | 11 |
| **Algeria** | 600 | high | 1.4 | 0.2 | 2.5 | 6 | 1 | 11 |
| **Brazil** | 507 | high | 1.6 | 1.1 | 1.6 | 6 | 4 | 6 |
| **Mexico** | 343 | high | 1.4 | 1.1 | 1.6 | 3 | 3 | 4 |
| **Slovenia** | 365 | high | 1.3 | 1.1 | 1.6 | 3 | 3 | 4 |
| **Somalia** | 470 | high | 1 | 0.6 | 3.1 | 3 | 2 | 10 |
| **Germany** | 23,177 | low | 0.5 | 0.3 | 0.9 | 81 | 49 | 146 |
| **Austria** | 5,634 | low | 0.5 | 0.1 | 0.7 | 20 | 4 | 28 |
| **France** | 2,799 | low | 0.7 | 0.5 | 0.8 | 14 | 10 | 16 |
| **United Kingdom** | 2,639 | low | 0.6 | 0.4 | 1.2 | 11 | 7 | 22 |
| **Iran** | 1,992 | low | 0.5 | 0.2 | 1 | 7 | 3 | 14 |
| **Czech Republic** | 1,411 | low | 0.7 | 0.2 | 0.7 | 7 | 2 | 7 |
| **Belgium** | 1,047 | low | 0.9 | 0.1 | 1.2 | 7 | 1 | 9 |
| **South Korea** | 935 | low | 0.8 | 0.2 | 2.1 | 5 | 1 | 14 |
| **India** | 917 | low | 0.8 | 0.4 | 1 | 5 | 3 | 6 |
| **Sweden** | 1,101 | low | 0.6 | 0.5 | 0.7 | 5 | 4 | 5 |
| **Norway** | 917 | low | 0.7 | 0.6 | 0.9 | 4 | 4 | 6 |
| **Netherlands** | 2,179 | low | 0.2 | 0.1 | 0.4 | 3 | 2 | 6 |
| **Cuba** | 514 | low | 0.8 | 0.2 | 1.3 | 3 | 1 | 5 |
| **Finland** | 460 | low | 0.7 | 0.6 | 0.9 | 2 | 2 | 3 |

## Iceland

| **Country of birth** | **Population (>15 yrs)** | **Anti-HCV endemicity** | **Anti-HCV prevalence** | | | **Estimated no. of CHC cases** | | |
| --- | --- | --- | --- | --- | --- | --- | --- | --- |
|  |  |  | **%** | **Lower limit** | **Upper limit** | **Cases** | **Lower** | **Upper** |
| **Poland** | 8,577 | high | 1.1 | 0.6 | 1.9 | 66 | 36 | 114 |
| **Lithuania** | 1,269 | high | 2.9 | 0.7 | 3 | 26 | 6 | 27 |
| **Thailand** | 1,068 | high | 2.7 | 1.8 | 3.7 | 20 | 13 | 28 |
| **United States** | 1,516 | high | 1.3 | 1.2 | 2.4 | 14 | 13 | 25 |
| **Latvia** | 596 | high | 2.4 | 1.7 | 3.3 | 10 | 7 | 14 |
| **Russia** | 334 | high | 4.1 | 1.2 | 5.6 | 10 | 3 | 13 |
| **Italy** | 201 | high | 4.4 | 1.6 | 7.3 | 6 | 2 | 10 |
| **Ukraine** | 242 | high | 3.6 | 0.9 | 4.5 | 6 | 2 | 8 |
| **Romania** | 240 | high | 3.2 | 2.9 | 3.6 | 5 | 5 | 6 |
| **Portugal** | 412 | high | 1.8 | 0.5 | 2.9 | 5 | 1 | 8 |
| **Vietnam** | 530 | high | 1 | 0.8 | 1.8 | 4 | 3 | 7 |
| **China** | 368 | high | 1.3 | 0.4 | 2 | 3 | 1 | 5 |
| **Spain** | 272 | high | 1.7 | 0.4 | 2.6 | 3 | 1 | 5 |
| **Serbia** | 297 | high | 1.3 | 1.1 | 1.6 | 3 | 2 | 3 |
| **Estonia** | 112 | high | 3.3 | 1.6 | 4.5 | 3 | 1 | 4 |
| **Former Soviet Republic** | 112 | high | 3.3 | 1.6 | 4.5 | 3 | 1 | 4 |
| **Canada** | 228 | high | 1.1 | 0.6 | 1.3 | 2 | 1 | 2 |
| **Morocco** | 149 | high | 1.6 | 0.6 | 1.9 | 2 | 1 | 2 |
| **Slovakia** | 165 | high | 1.4 | 0.9 | 2 | 2 | 1 | 2 |
| **Kosovo** | 156 | high | 1.3 | 1.1 | 1.6 | 1 | 1 | 2 |
| **Croatia** | 149 | high | 1.3 | 1.1 | 1.6 | 1 | 1 | 2 |
| **Brazil** | 111 | high | 1.6 | 1.1 | 1.6 | 1 | 1 | 1 |
| **Switzerland** | 117 | high | 1.5 | 0.7 | 1.8 | 1 | 1 | 1 |
| **Bosnia and Herzegovina** | 132 | high | 1.3 | 1.1 | 1.6 | 1 | 1 | 1 |
| **Sri Lanka** | 158 | high | 1 | 0.8 | 1.8 | 1 | 1 | 2 |
| **Bulgaria** | 140 | high | 1.1 | 0.3 | 2.4 | 1 | 0 | 2 |
| **Colombia** | 146 | high | 1 | 0.8 | 1.4 | 1 | 1 | 1 |
| **Australia** | 85 | high | 1.7 | 1.2 | 2.3 | 1 | 1 | 1 |
| **Japan** | 83 | high | 1.5 | 0.5 | 2.2 | 1 | 0 | 1 |
| **Former Yugoslav Republic** | 89 | high | 1.3 | 1.1 | 1.6 | 1 | 1 | 1 |
| **Former Yugoslavia** | 85 | high | 1.3 | 1.1 | 1.6 | 1 | 1 | 1 |
| **Nepal** | 90 | high | 1.1 | 0.7 | 1.5 | 1 | 0 | 1 |
| **Kenya** | 82 | high | 1 | 0.6 | 3.1 | 1 | 0 | 2 |
| **Denmark** | 2,075 | low | 0.7 | 0.5 | 0.7 | 10 | 7 | 10 |
| **Philippines** | 1,384 | low | 0.9 | 0.3 | 2 | 9 | 3 | 19 |
| **Sweden** | 1,597 | low | 0.6 | 0.5 | 0.7 | 7 | 6 | 8 |
| **Germany** | 1,338 | low | 0.5 | 0.3 | 0.9 | 5 | 3 | 8 |
| **United Kingdom** | 958 | low | 0.6 | 0.4 | 1.2 | 4 | 3 | 8 |
| **Norway** | 704 | low | 0.7 | 0.6 | 0.9 | 3 | 3 | 4 |
| **France*** | 399 | low | 0.7 | 0.5 | 0.8 | 2 | 1 | 2 |
| **Faeroe Islands (DK)** | 294 | low | 0.9 | 0.7 | 1.5 | 2 | 1 | 3 |
| **India** | 206 | low | 0.8 | 0.4 | 1 | 1 | 1 | 1 |
| **Hungary** | 155 | low | 0.8 | 0.4 | 2.7 | 1 | 0 | 3 |
| **Finland** | 155 | low | 0.7 | 0.6 | 0.9 | 1 | 1 | 1 |
| **Czech Republic** | 140 | low | 0.7 | 0.2 | 0.7 | 1 | 0 | 1 |
| **Indonesia** | 114 | low | 0.8 | 0.4 | 2 | 1 | 0 | 2 |
| **Luxembourg** | 86 | low | 0.9 | 0.6 | 0.9 | 1 | 0 | 1 |
| **Austria** | 104 | low | 0.5 | 0.1 | 0.7 | 0 | 0 | 1 |
| **Netherlands** | 201 | low | 0.2 | 0.1 | 0.4 | 0 | 0 | 1 |

## Republic of Ireland

| **Country of birth** | **Population (>15 yrs)** | **Anti-HCV endemicity** | **Anti-HCV prevalence** | | | **Estimated no. of CHC cases** | | |
| --- | --- | --- | --- | --- | --- | --- | --- | --- |
|  |  |  | **%** | **Lower limit** | **Upper limit** | **Cases** | **Lower** | **Upper** |
| **Nigeria** | 20,819 | high | 8.4 | 3.9 | 12.8 | 1,224 | 568 | 1,865 |
| **Poland** | 93,763 | high | 1.1 | 0.6 | 1.9 | 722 | 394 | 1,247 |
| **Lithuania** | 28,152 | high | 2.9 | 0.7 | 3 | 571 | 138 | 591 |
| **Romania** | 15,106 | high | 3.2 | 2.9 | 3.6 | 338 | 307 | 381 |
| **Pakistan** | 8,887 | high | 5 | 4.4 | 5.5 | 311 | 274 | 342 |
| **Latvia** | 16,249 | high | 2.4 | 1.7 | 3.3 | 273 | 193 | 375 |
| **Italy** | 6,276 | high | 4.4 | 1.6 | 7.3 | 193 | 70 | 321 |
| **Egypt** | 1,539 | high | 15.7 | 13.9 | 17.5 | 169 | 150 | 189 |
| **Russia** | 5,437 | high | 4.1 | 1.2 | 5.6 | 156 | 46 | 213 |
| **United States** | 17,094 | high | 1.3 | 1.2 | 2.4 | 156 | 144 | 287 |
| **China** | 13,002 | high | 1.3 | 0.4 | 2 | 118 | 36 | 182 |
| **Ukraine** | 4,690 | high | 3.6 | 0.9 | 4.5 | 118 | 30 | 148 |
| **Brazil** | 10,374 | high | 1.6 | 1.1 | 1.6 | 116 | 80 | 116 |
| **South Africa** | 9,013 | high | 1.7 | 1 | 2.5 | 107 | 63 | 158 |
| **Slovakia** | 9,084 | high | 1.4 | 0.9 | 2 | 89 | 57 | 127 |
| **Moldova** | 3,834 | high | 3.3 | 1.6 | 4.5 | 89 | 43 | 121 |
| **Congo** | 2,443 | high | 4.2 | 2.4 | 9.2 | 72 | 41 | 157 |
| **Australia** | 5,958 | high | 1.7 | 1.2 | 2.3 | 71 | 50 | 96 |
| **Spain** | 5,814 | high | 1.7 | 0.4 | 2.6 | 69 | 16 | 106 |
| **Ghana** | 1,466 | high | 5.3 | 2.9 | 9.1 | 54 | 30 | 93 |
| **Estonia** | 2,100 | high | 3.3 | 1.6 | 4.5 | 49 | 24 | 66 |
| **Malaysia** | 4,353 | high | 1.5 | 0.3 | 7.7 | 46 | 9 | 235 |
| **Canada** | 5,544 | high | 1.1 | 0.6 | 1.3 | 43 | 23 | 50 |
| **Iraq** | 1,792 | high | 3.2 | 0.3 | 3.2 | 40 | 4 | 40 |
| **Thailand** | 1,855 | high | 2.7 | 1.8 | 3.7 | 35 | 23 | 48 |
| **Zimbabwe** | 3,061 | high | 1.6 | 1 | 9.1 | 34 | 21 | 195 |
| **New Zealand** | 2,213 | high | 1.9 | 0.8 | 2.2 | 29 | 12 | 34 |
| **Bangladesh** | 3,211 | high | 1.3 | 0.2 | 2.2 | 29 | 4 | 49 |
| **Portugal** | 1,828 | high | 1.8 | 0.5 | 2.9 | 23 | 6 | 37 |
| **Mauritius** | 3,279 | high | 1 | 0.8 | 1.8 | 23 | 18 | 41 |
| **Saudi Arabia** | 1,537 | high | 1.5 | 0.6 | 7.3 | 16 | 6 | 79 |
| **Algeria** | 1,617 | high | 1.4 | 0.2 | 2.5 | 16 | 2 | 28 |
| **Bosnia and Herzegovina** | 1,444 | high | 1.3 | 1.1 | 1.6 | 13 | 11 | 16 |
| **Belarus** | 1,407 | high | 1.3 | 0.9 | 2.9 | 13 | 9 | 29 |
| **Switzerland** | 1,200 | high | 1.5 | 0.7 | 1.8 | 13 | 6 | 15 |
| **Bulgaria** | 1,574 | high | 1.1 | 0.3 | 2.4 | 12 | 3 | 26 |
| **Sudan** | 1,724 | high | 1 | 0.6 | 3.1 | 12 | 7 | 37 |
| **Somalia** | 1,570 | high | 1 | 0.6 | 3.1 | 11 | 7 | 34 |
| **Turkey** | 1,462 | high | 1 | 0.7 | 1.1 | 10 | 7 | 11 |
| **United Kingdom** | 208,916 | low | 0.6 | 0.4 | 1.2 | 877 | 585 | 1,755 |
| **India** | 18,390 | low | 0.8 | 0.4 | 1 | 103 | 51 | 129 |
| **Philippines** | 14,955 | low | 0.9 | 0.3 | 2 | 94 | 31 | 209 |
| **France** | 8,622 | low | 0.7 | 0.5 | 0.8 | 42 | 30 | 48 |
| **Germany** | 11,268 | low | 0.5 | 0.3 | 0.9 | 39 | 24 | 71 |
| **Hungary** | 6,265 | low | 0.8 | 0.4 | 2.7 | 35 | 18 | 118 |
| **Czech Republic** | 4,672 | low | 0.7 | 0.2 | 0.7 | 23 | 7 | 23 |
| **Sweden** | 1,452 | low | 0.6 | 0.5 | 0.7 | 6 | 5 | 7 |
| **Netherlands** | 3,809 | low | 0.2 | 0.1 | 0.4 | 5 | 3 | 11 |
| **Iran** | 1,197 | low | 0.5 | 0.2 | 1 | 4 | 2 | 8 |

## Italy

| **Country of birth** | **Population (>15 yrs)** | **Anti-HCV endemicity** | **Anti-HCV prevalence** | | | **Estimated no. of CHC cases** | | |
| --- | --- | --- | --- | --- | --- | --- | --- | --- |
|  |  |  | **%** | **Lower limit** | **Upper limit** | **Cases** | **Lower** | **Upper** |
| **Romania** | 933,854 | high | 3.2 | 2.9 | 3.6 | 20,918 | 18,957 | 23,533 |
| **Egypt** | 95,572 | high | 15.7 | 13.9 | 17.5 | 10,503 | 9,299 | 11,708 |
| **Albania** | 411,994 | high | 2.4 | 2 | 2.8 | 6,921 | 5,768 | 8,075 |
| **Ukraine** | 199,021 | high | 3.6 | 0.9 | 4.5 | 5,015 | 1,254 | 6,269 |
| **Morocco** | 385,177 | high | 1.6 | 0.6 | 1.9 | 4,314 | 1,618 | 5,123 |
| **Moldova** | 146,367 | high | 3.3 | 1.6 | 4.5 | 3,381 | 1,639 | 4,611 |
| **Nigeria** | 51,022 | high | 8.4 | 3.9 | 12.8 | 3,000 | 1,393 | 4,572 |
| **Senegal** | 74,211 | high | 5.3 | 2.9 | 9.1 | 2,753 | 1,506 | 4,727 |
| **Pakistan** | 66,303 | high | 5 | 4.4 | 5.5 | 2,321 | 2,042 | 2,553 |
| **Russia** | 69,963 | high | 4.1 | 1.2 | 5.6 | 2,008 | 588 | 2,743 |
| **Switzerland** | 185,436 | high | 1.5 | 0.7 | 1.8 | 1,947 | 909 | 2,336 |
| **China** | 184,917 | high | 1.3 | 0.4 | 2 | 1,683 | 518 | 2,589 |
| **Ghana** | 41,851 | high | 5.3 | 2.9 | 9.1 | 1,553 | 850 | 2,666 |
| **Brazil** | 100,579 | high | 1.6 | 1.1 | 1.6 | 1,126 | 774 | 1,126 |
| **Tunisia** | 106,217 | high | 1.3 | 0.3 | 2.5 | 967 | 223 | 1,859 |
| **Peru** | 107,531 | high | 1.2 | 0.4 | 1.6 | 903 | 301 | 1,204 |
| **Poland** | 116,457 | high | 1.1 | 0.6 | 1.9 | 897 | 489 | 1,549 |
| **Ivory Coast** | 23,090 | high | 5.3 | 2.9 | 9.1 | 857 | 469 | 1,471 |
| **Argentina** | 74,233 | high | 1.5 | 0.5 | 2.5 | 779 | 260 | 1,299 |
| **Bangladesh** | 81,677 | high | 1.3 | 0.2 | 2.2 | 743 | 114 | 1,258 |
| **FYR Macedonia** | 66,995 | high | 1.3 | 1.1 | 1.6 | 610 | 516 | 750 |
| **Sri Lanka** | 79,486 | high | 1 | 0.8 | 1.8 | 556 | 445 | 1,002 |
| **Venezuela** | 46,119 | high | 1.5 | 0.3 | 2.6 | 484 | 97 | 839 |
| **United States** | 50,793 | high | 1.3 | 1.2 | 2.4 | 462 | 427 | 853 |
| **Bulgaria** | 53,304 | high | 1.1 | 0.3 | 2.4 | 410 | 112 | 896 |
| **Serbia** | 43,272 | high | 1.3 | 1.1 | 1.6 | 394 | 333 | 485 |
| **Spain** | 28,870 | high | 1.7 | 0.4 | 2.6 | 344 | 81 | 525 |
| **Kosovo** | 36,851 | high | 1.3 | 1.1 | 1.6 | 335 | 284 | 413 |
| **Libya** | 35,387 | high | 1.2 | 1.2 | 2.3 | 297 | 297 | 570 |
| **Belarus** | 31,146 | high | 1.3 | 0.9 | 2.9 | 283 | 196 | 632 |
| **Ethiopia** | 28,110 | high | 1.3 | 0.7 | 5.8 | 256 | 138 | 1,141 |
| **Greece** | 18,531 | high | 1.9 | 0.5 | 2.6 | 246 | 65 | 337 |
| **Colombia** | 33,526 | high | 1 | 0.8 | 1.4 | 235 | 188 | 329 |
| **Australia** | 19,231 | high | 1.7 | 1.2 | 2.3 | 229 | 162 | 310 |
| **Croatia** | 24,917 | high | 1.3 | 1.1 | 1.6 | 227 | 192 | 279 |
| **Algeria** | 22,431 | high | 1.4 | 0.2 | 2.5 | 220 | 31 | 393 |
| **Canada** | 24,589 | high | 1.1 | 0.6 | 1.3 | 189 | 103 | 224 |
| **Turkey** | 18,985 | high | 1 | 0.7 | 1.1 | 133 | 93 | 146 |
| **Philippines** | 130,100 | low | 0.9 | 0.3 | 2 | 820 | 273 | 1,821 |
| **Germany** | 203,827 | low | 0.5 | 0.3 | 0.9 | 713 | 428 | 1,284 |
| **India** | 116,660 | low | 0.8 | 0.4 | 1 | 653 | 327 | 817 |
| **France** | 129,899 | low | 0.7 | 0.5 | 0.8 | 637 | 455 | 727 |
| **Ecuador** | 82,999 | low | 0.9 | 0.4 | 1.3 | 523 | 232 | 755 |
| **Belgium** | 44,480 | low | 0.9 | 0.1 | 1.2 | 280 | 31 | 374 |
| **United Kingdom** | 62,585 | low | 0.6 | 0.4 | 1.2 | 263 | 175 | 526 |
| **Dominican Republic** | 36,748 | low | 0.8 | 0.2 | 1.3 | 206 | 51 | 334 |
| **Cuba** | 29,866 | low | 0.8 | 0.2 | 1.3 | 167 | 42 | 272 |
| **Bolivia** | 13,566 | low | 0.9 | 0.4 | 1.3 | 85 | 38 | 123 |
| **Iran** | 13,830 | low | 0.5 | 0.2 | 1 | 48 | 19 | 97 |
| **Austria** | 13,756 | low | 0.5 | 0.1 | 0.7 | 48 | 10 | 67 |

## Latvia

| **Country of birth** | **Population (>15 yrs)** | **Anti-HCV endemicity** | **Anti-HCV prevalence** | | | **Estimated no. of CHC cases** | | |
| --- | --- | --- | --- | --- | --- | --- | --- | --- |
|  |  |  | **%** | **Lower limit** | **Upper limit** | **Cases** | **Lower** | **Upper** |
| **Russia** | 145,768 | high | 4.1 | 1.2 | 5.6 | 4,184 | 1,224 | 5,714 |
| **Ukraine** | 35,597 | high | 3.6 | 0.9 | 4.5 | 897 | 224 | 1,121 |
| **Belarus** | 51,433 | high | 1.3 | 0.9 | 2.9 | 468 | 324 | 1,044 |
| **Lithuania** | 17,756 | high | 2.9 | 0.7 | 3 | 360 | 87 | 373 |
| **Uzbekistan** | 2,009 | high | 11.3 | 6.4 | 13.1 | 159 | 90 | 184 |
| **Kazakhstan** | 6,186 | high | 3.3 | 1 | 6.7 | 143 | 43 | 290 |
| **Georgia** | 1,374 | high | 6.7 | 5.6 | 7.3 | 64 | 54 | 70 |
| **Estonia** | 2,674 | high | 3.3 | 1.6 | 4.5 | 62 | 30 | 84 |
| **Azerbaijan** | 2,002 | high | 3.1 | 1 | 6.7 | 43 | 14 | 94 |
| **Moldova** | 1,742 | high | 3.3 | 1.6 | 4.5 | 40 | 20 | 55 |
| **Armenia** | 729 | high | 5.4 | 3.5 | 6.8 | 28 | 18 | 35 |
| **Turkmenistan** | 620 | high | 5.6 | 1.1 | 6.7 | 24 | 5 | 29 |
| **Kyrgyzstan** | 859 | high | 2.5 | 1.6 | 6.7 | 15 | 10 | 40 |
| **Tajikistan** | 544 | high | 3.1 | 1.1 | 6.7 | 12 | 4 | 26 |
| **Mongolia** | 115 | high | 10.8 | 8.7 | 15.6 | 9 | 7 | 13 |
| **Poland** | 845 | high | 1.1 | 0.6 | 1.9 | 7 | 4 | 11 |
| **Italy** | 113 | high | 4.4 | 1.6 | 7.3 | 3 | 1 | 6 |
| **Egypt** | 21 | high | 15.7 | 13.9 | 17.5 | 2 | 2 | 3 |
| **United States** | 227 | high | 1.3 | 1.2 | 2.4 | 2 | 2 | 4 |
| **China** | 206 | high | 1.3 | 0.4 | 2 | 2 | 1 | 3 |
| **Romania** | 70 | high | 3.2 | 2.9 | 3.6 | 2 | 1 | 2 |
| **Bulgaria** | 147 | high | 1.1 | 0.3 | 2.4 | 1 | 0 | 2 |
| **Australia** | 73 | high | 1.7 | 1.2 | 2.3 | 1 | 1 | 1 |
| **Pakistan** | 19 | high | 5 | 4.4 | 5.5 | 1 | 1 | 1 |
| **Canada** | 76 | high | 1.1 | 0.6 | 1.3 | 1 | 0 | 1 |
| **Turkey** | 81 | high | 1 | 0.7 | 1.1 | 1 | 0 | 1 |
| **Lebanon** | 24 | high | 3.1 | 2.5 | 3.9 | 1 | 0 | 1 |
| **Spain** | 41 | high | 1.7 | 0.4 | 2.6 | 0 | 0 | 1 |
| **Israel** | 25 | high | 2 | 0.9 | 2 | 0 | 0 | 0 |
| **Greece** | 17 | high | 1.9 | 0.5 | 2.6 | 0 | 0 | 0 |
| **Venezuela** | 20 | high | 1.5 | 0.3 | 2.6 | 0 | 0 | 0 |
| **Argentina** | 17 | high | 1.5 | 0.5 | 2.5 | 0 | 0 | 0 |
| **Slovakia** | 18 | high | 1.4 | 0.9 | 2 | 0 | 0 | 0 |
| **Nepal** | 20 | high | 1.1 | 0.7 | 1.5 | 0 | 0 | 0 |
| **Germany** | 1,942 | low | 0.5 | 0.3 | 0.9 | 7 | 4 | 12 |
| **Hungary** | 198 | low | 0.8 | 0.4 | 2.7 | 1 | 1 | 4 |
| **United Kingdom** | 211 | low | 0.6 | 0.4 | 1.2 | 1 | 1 | 2 |
| **Sweden** | 154 | low | 0.6 | 0.5 | 0.7 | 1 | 1 | 1 |
| **Czech Republic** | 123 | low | 0.7 | 0.2 | 0.7 | 1 | 0 | 1 |
| **France** | 122 | low | 0.7 | 0.5 | 0.8 | 1 | 0 | 1 |
| **Denmark** | 122 | low | 0.7 | 0.5 | 0.7 | 1 | 0 | 1 |
| **Finland** | 111 | low | 0.7 | 0.6 | 0.9 | 1 | 0 | 1 |
| **India** | 92 | low | 0.8 | 0.4 | 1 | 1 | 0 | 1 |
| **Austria** | 75 | low | 0.5 | 0.1 | 0.7 | 0 | 0 | 0 |
| **Cuba** | 46 | low | 0.8 | 0.2 | 1.3 | 0 | 0 | 0 |
| **Norway** | 50 | low | 0.7 | 0.6 | 0.9 | 0 | 0 | 0 |
| **Belgium** | 28 | low | 0.9 | 0.1 | 1.2 | 0 | 0 | 0 |
| **Netherlands** | 73 | low | 0.2 | 0.1 | 0.4 | 0 | 0 | 0 |
| **South Korea** | 17 | low | 0.8 | 0.2 | 2.1 | 0 | 0 | 0 |
| **Iran** | 24 | low | 0.5 | 0.2 | 1 | 0 | 0 | 0 |

## Liechtenstein

| **Country of birth** | **Population (>15 yrs)** | **Anti-HCV endemicity** | **Anti-HCV prevalence** | | | **Estimated no. of CHC cases** | | |
| --- | --- | --- | --- | --- | --- | --- | --- | --- |
|  |  |  | **%** | **Lower limit** | **Upper limit** | **Cases** | **Lower** | **Upper** |
| **Switzerland** | 10,565 | high | 1.5 | 0.7 | 1.8 | 111 | 52 | 133 |
| **Italy** | 846 | high | 4.4 | 1.6 | 7.3 | 26 | 9 | 43 |
| **Portugal** | 394 | high | 1.8 | 0.5 | 2.9 | 5 | 1 | 8 |
| **Turkey** | 597 | high | 1 | 0.7 | 1.1 | 4 | 3 | 5 |
| **Spain** | 260 | high | 1.7 | 0.4 | 2.6 | 3 | 1 | 5 |
| **Bosnia and Herzegovina** | 299 | high | 1.3 | 1.1 | 1.6 | 3 | 2 | 3 |
| **Kosovo** | 230 | high | 1.3 | 1.1 | 1.6 | 2 | 2 | 3 |
| **Brazil** | 157 | high | 1.6 | 1.1 | 1.6 | 2 | 1 | 2 |
| **Egypt** | 15 | high | 15.7 | 13.9 | 17.5 | 2 | 1 | 2 |
| **Russia** | 45 | high | 4.1 | 1.2 | 5.6 | 1 | 0 | 2 |
| **Thailand** | 68 | high | 2.7 | 1.8 | 3.7 | 1 | 1 | 2 |
| **Serbia** | 138 | high | 1.3 | 1.1 | 1.6 | 1 | 1 | 2 |
| **Croatia** | 128 | high | 1.3 | 1.1 | 1.6 | 1 | 1 | 1 |
| **Slovenia** | 111 | high | 1.3 | 1.1 | 1.6 | 1 | 1 | 1 |
| **Former Yugoslav Republic** | 111 | high | 1.3 | 1.1 | 1.6 | 1 | 1 | 1 |
| **United States** | 110 | high | 1.3 | 1.2 | 2.4 | 1 | 1 | 2 |
| **Ukraine** | 29 | high | 3.6 | 0.9 | 4.5 | 1 | 0 | 1 |
| **FYR Macedonia** | 80 | high | 1.3 | 1.1 | 1.6 | 1 | 1 | 1 |
| **Romania** | 31 | high | 3.2 | 2.9 | 3.6 | 1 | 1 | 1 |
| **Greece** | 47 | high | 1.9 | 0.5 | 2.6 | 1 | 0 | 1 |
| **China** | 68 | high | 1.3 | 0.4 | 2 | 1 | 0 | 1 |
| **Poland** | 53 | high | 1.1 | 0.6 | 1.9 | 0 | 0 | 1 |
| **Slovakia** | 33 | high | 1.4 | 0.9 | 2 | 0 | 0 | 0 |
| **South Africa** | 27 | high | 1.7 | 1 | 2.5 | 0 | 0 | 0 |
| **Argentina** | 24 | high | 1.5 | 0.5 | 2.5 | 0 | 0 | 0 |
| **Laos** | 35 | high | 1 | 0.8 | 1.8 | 0 | 0 | 0 |
| **Colombia** | 32 | high | 1 | 0.8 | 1.4 | 0 | 0 | 0 |
| **Mexico** | 20 | high | 1.4 | 1.1 | 1.6 | 0 | 0 | 0 |
| **Tunisia** | 20 | high | 1.3 | 0.3 | 2.5 | 0 | 0 | 0 |
| **Venezuela** | 15 | high | 1.5 | 0.3 | 2.6 | 0 | 0 | 0 |
| **Morocco** | 13 | high | 1.6 | 0.6 | 1.9 | 0 | 0 | 0 |
| **Peru** | 17 | high | 1.2 | 0.4 | 1.6 | 0 | 0 | 0 |
| **Japan** | 13 | high | 1.5 | 0.5 | 2.2 | 0 | 0 | 0 |
| **Canada** | 15 | high | 1.1 | 0.6 | 1.3 | 0 | 0 | 0 |
| **Ireland** | 13 | high | 1.1 | 0.7 | 1.6 | 0 | 0 | 0 |
| **Somalia** | 13 | high | 1 | 0.6 | 3.1 | 0 | 0 | 0 |
| **Austria** | 3,429 | low | 0.5 | 0.1 | 0.7 | 12 | 2 | 17 |
| **Germany** | 1,652 | low | 0.5 | 0.3 | 0.9 | 6 | 3 | 10 |
| **France** | 111 | low | 0.7 | 0.5 | 0.8 | 1 | 0 | 1 |
| **Hungary** | 61 | low | 0.8 | 0.4 | 2.7 | 0 | 0 | 1 |
| **Dominican Republic** | 57 | low | 0.8 | 0.2 | 1.3 | 0 | 0 | 1 |
| **Czech Republic** | 54 | low | 0.7 | 0.2 | 0.7 | 0 | 0 | 0 |
| **United Kingdom** | 57 | low | 0.6 | 0.4 | 1.2 | 0 | 0 | 0 |
| **Philippines** | 37 | low | 0.9 | 0.3 | 2 | 0 | 0 | 1 |
| **Belgium** | 37 | low | 0.9 | 0.1 | 1.2 | 0 | 0 | 0 |
| **India** | 24 | low | 0.8 | 0.4 | 1 | 0 | 0 | 0 |
| **Netherlands** | 71 | low | 0.2 | 0.1 | 0.4 | 0 | 0 | 0 |
| **Sweden** | 22 | low | 0.6 | 0.5 | 0.7 | 0 | 0 | 0 |
| **Denmark** | 14 | low | 0.7 | 0.5 | 0.7 | 0 | 0 | 0 |

## Lithuania

| **Country of birth** | **Population (>15 yrs)** | **Anti-HCV endemicity** | **Anti-HCV prevalence** | | | **Estimated no. of CHC cases** | | |
| --- | --- | --- | --- | --- | --- | --- | --- | --- |
|  |  |  | **%** | **Lower limit** | **Upper limit** | **Cases** | **Lower** | **Upper** |
| **Russia** | 59,191 | high | 4.1 | 1.2 | 5.6 | 1,699 | 497 | 2,320 |
| **Belarus** | 35,262 | high | 1.3 | 0.9 | 2.9 | 321 | 222 | 716 |
| **Ukraine** | 12,396 | high | 3.6 | 0.9 | 4.5 | 312 | 78 | 390 |
| **Kazakhstan** | 4,479 | high | 3.3 | 1 | 6.7 | 103 | 31 | 210 |
| **Latvia** | 5,483 | high | 2.4 | 1.7 | 3.3 | 92 | 65 | 127 |
| **Uzbekistan** | 971 | high | 11.3 | 6.4 | 13.1 | 77 | 43 | 89 |
| **Georgia** | 666 | high | 6.7 | 5.6 | 7.3 | 31 | 26 | 34 |
| **Armenia** | 561 | high | 5.4 | 3.5 | 6.8 | 21 | 14 | 27 |
| **Azerbaijan** | 879 | high | 3.1 | 1 | 6.7 | 19 | 6 | 41 |
| **Estonia** | 791 | high | 3.3 | 1.6 | 4.5 | 18 | 9 | 25 |
| **Poland** | 2,253 | high | 1.1 | 0.6 | 1.9 | 17 | 9 | 30 |
| **Moldova** | 606 | high | 3.3 | 1.6 | 4.5 | 14 | 7 | 19 |
| **Turkmenistan** | 244 | high | 5.6 | 1.1 | 6.7 | 10 | 2 | 11 |
| **Ireland** | 922 | high | 1.1 | 0.7 | 1.6 | 7 | 5 | 10 |
| **Tajikistan** | 327 | high | 3.1 | 1.1 | 6.7 | 7 | 3 | 15 |
| **Kyrgyzstan** | 385 | high | 2.5 | 1.6 | 6.7 | 7 | 4 | 18 |
| **Italy** | 215 | high | 4.4 | 1.6 | 7.3 | 7 | 2 | 11 |
| **United States** | 691 | high | 1.3 | 1.2 | 2.4 | 6 | 6 | 12 |
| **Spain** | 450 | high | 1.7 | 0.4 | 2.6 | 5 | 1 | 8 |
| **Mongolia** | 64 | high | 10.8 | 8.7 | 15.6 | 5 | 4 | 7 |
| **Egypt** | 33 | high | 15.7 | 13.9 | 17.5 | 4 | 3 | 4 |
| **Romania** | 142 | high | 3.2 | 2.9 | 3.6 | 3 | 3 | 4 |
| **Nigeria** | 37 | high | 8.4 | 3.9 | 12.8 | 2 | 1 | 3 |
| **China** | 117 | high | 1.3 | 0.4 | 2 | 1 | 0 | 2 |
| **Lebanon** | 45 | high | 3.1 | 2.5 | 3.9 | 1 | 1 | 1 |
| **Turkey** | 89 | high | 1 | 0.7 | 1.1 | 1 | 0 | 1 |
| **Argentina** | 58 | high | 1.5 | 0.5 | 2.5 | 1 | 0 | 1 |
| **Brasil** | 54 | high | 1.6 | 1.1 | 1.6 | 1 | 0 | 1 |
| **Portugal** | 42 | high | 1.8 | 0.5 | 2.9 | 1 | 0 | 1 |
| **Greece** | 38 | high | 1.9 | 0.5 | 2.6 | 1 | 0 | 1 |
| **Israel** | 31 | high | 2 | 0.9 | 2 | 0 | 0 | 0 |
| **Bulgaria** | 53 | high | 1.1 | 0.3 | 2.4 | 0 | 0 | 1 |
| **Australia** | 33 | high | 1.7 | 1.2 | 2.3 | 0 | 0 | 1 |
| **Switzerland** | 31 | high | 1.5 | 0.7 | 1.8 | 0 | 0 | 0 |
| **Canada** | 35 | high | 1.1 | 0.6 | 1.3 | 0 | 0 | 0 |
| **Vietnam** | 34 | high | 1 | 0.8 | 1.8 | 0 | 0 | 0 |
| **United Kingdom** | 2,091 | low | 0.6 | 0.4 | 1.2 | 9 | 6 | 18 |
| **Germany** | 1,359 | low | 0.5 | 0.3 | 0.9 | 5 | 3 | 9 |
| **Norway** | 509 | low | 0.7 | 0.6 | 0.9 | 2 | 2 | 3 |
| **France** | 247 | low | 0.7 | 0.5 | 0.8 | 1 | 1 | 1 |
| **Denmark** | 224 | low | 0.7 | 0.5 | 0.7 | 1 | 1 | 1 |
| **Belgium** | 148 | low | 0.9 | 0.1 | 1.2 | 1 | 0 | 1 |
| **Sweden** | 175 | low | 0.6 | 0.5 | 0.7 | 1 | 1 | 1 |
| **Hungary** | 93 | low | 0.8 | 0.4 | 2.7 | 1 | 0 | 2 |
| **Finland** | 79 | low | 0.7 | 0.6 | 0.9 | 0 | 0 | 0 |
| **Czech Republic** | 67 | low | 0.7 | 0.2 | 0.7 | 0 | 0 | 0 |
| **Iceland** | 34 | low | 0.9 | 0.7 | 1.5 | 0 | 0 | 0 |
| **Austria** | 47 | low | 0.5 | 0.1 | 0.7 | 0 | 0 | 0 |
| **Netherlands** | 82 | low | 0.2 | 0.1 | 0.4 | 0 | 0 | 0 |

## Luxembourg

| **Country of birth** | **Population (>15 yrs)** | **Anti-HCV endemicity** | **Anti-HCV prevalence** | | | **Estimated no. of CHC cases** | | |
| --- | --- | --- | --- | --- | --- | --- | --- | --- |
|  |  |  | **%** | **Lower limit** | **Upper limit** | **Cases** | **Lower** | **Upper** |
| **Portugal** | 56,208 | high | 1.8 | 0.5 | 2.9 | 708 | 197 | 1,141 |
| **Italy** | 12,205 | high | 4.4 | 1.6 | 7.3 | 376 | 137 | 624 |
| **Cape Verde** | 4,266 | high | 5.3 | 2.9 | 9.1 | 158 | 87 | 272 |
| **Romania** | 1,779 | high | 3.2 | 2.9 | 3.6 | 40 | 36 | 45 |
| **Cameroon** | 444 | high | 11.6 | 4.3 | 29.7 | 36 | 13 | 92 |
| **Russia** | 1,180 | high | 4.1 | 1.2 | 5.6 | 34 | 10 | 46 |
| **Spain** | 2,690 | high | 1.7 | 0.4 | 2.6 | 32 | 8 | 49 |
| **Montenegro** | 2,628 | high | 1.3 | 1.1 | 1.6 | 24 | 20 | 29 |
| **Poland** | 2,660 | high | 1.1 | 0.6 | 1.9 | 20 | 11 | 35 |
| **Bosnia and Herzegovina** | 2,060 | high | 1.3 | 1.1 | 1.6 | 19 | 16 | 23 |
| **Angola** | 636 | high | 4.2 | 2.4 | 9.2 | 19 | 11 | 41 |
| **Brazil** | 1,657 | high | 1.6 | 1.1 | 1.6 | 19 | 13 | 19 |
| **Congo** | 586 | high | 4.2 | 2.4 | 9.2 | 17 | 10 | 38 |
| **China** | 1,725 | high | 1.3 | 0.4 | 2 | 16 | 5 | 24 |
| **Guinea-Bissau** | 405 | high | 5.3 | 2.9 | 9.1 | 15 | 8 | 26 |
| **Greece** | 1,116 | high | 1.9 | 0.5 | 2.6 | 15 | 4 | 20 |
| **Serbia** | 1,619 | high | 1.3 | 1.1 | 1.6 | 15 | 12 | 18 |
| **United States** | 1,501 | high | 1.3 | 1.2 | 2.4 | 14 | 13 | 25 |
| **Ukraine** | 533 | high | 3.6 | 0.9 | 4.5 | 13 | 3 | 17 |
| **Morocco** | 1,062 | high | 1.6 | 0.6 | 1.9 | 12 | 4 | 14 |
| **Lithuania** | 466 | high | 2.9 | 0.7 | 3 | 9 | 2 | 10 |
| **Switzerland** | 893 | high | 1.5 | 0.7 | 1.8 | 9 | 4 | 11 |
| **Estonia** | 402 | high | 3.3 | 1.6 | 4.5 | 9 | 5 | 13 |
| **Albania** | 443 | high | 2.4 | 2 | 2.8 | 7 | 6 | 9 |
| **Ireland** | 865 | high | 1.1 | 0.7 | 1.6 | 7 | 4 | 10 |
| **Former Yugoslavia** | 711 | high | 1.3 | 1.1 | 1.6 | 6 | 5 | 8 |
| **Algeria** | 606 | high | 1.4 | 0.2 | 2.5 | 6 | 1 | 11 |
| **Bulgaria** | 745 | high | 1.1 | 0.3 | 2.4 | 6 | 2 | 13 |
| **Slovak Republic** | 532 | high | 1.4 | 0.9 | 2 | 5 | 3 | 7 |
| **Tunisia** | 455 | high | 1.3 | 0.3 | 2.5 | 4 | 1 | 8 |
| **Turkey** | 540 | high | 1 | 0.7 | 1.1 | 4 | 3 | 4 |
| **FYR Macedonia** | 411 | high | 1.3 | 1.1 | 1.6 | 4 | 3 | 5 |
| **Canada** | 466 | high | 1.1 | 0.6 | 1.3 | 4 | 2 | 4 |
| **France** | 25,918 | low | 0.7 | 0.5 | 0.8 | 127 | 91 | 145 |
| **Belgium** | 15,496 | low | 0.9 | 0.1 | 1.2 | 98 | 11 | 130 |
| **Germany** | 13,663 | low | 0.5 | 0.3 | 0.9 | 48 | 29 | 86 |
| **United Kingdom** | 3,898 | low | 0.6 | 0.4 | 1.2 | 16 | 11 | 33 |
| **Denmark** | 1,402 | low | 0.7 | 0.5 | 0.7 | 7 | 5 | 7 |
| **Sweden** | 1,268 | low | 0.6 | 0.5 | 0.7 | 5 | 4 | 6 |
| **Hungary** | 896 | low | 0.8 | 0.4 | 2.7 | 5 | 3 | 17 |
| **India** | 812 | low | 0.8 | 0.4 | 1 | 5 | 2 | 6 |
| **Netherlands** | 3,233 | low | 0.2 | 0.1 | 0.4 | 5 | 2 | 9 |
| **Finland** | 838 | low | 0.7 | 0.6 | 0.9 | 4 | 4 | 5 |
| **Korea** | 682 | low | 0.8 | 0.2 | 2.1 | 4 | 1 | 10 |
| **Philippines** | 460 | low | 0.9 | 0.3 | 2 | 3 | 1 | 6 |
| **Austria** | 752 | low | 0.5 | 0.1 | 0.7 | 3 | 1 | 4 |
| **Iceland** | 401 | low | 0.9 | 0.7 | 1.5 | 3 | 2 | 4 |
| **Iran** | 615 | low | 0.5 | 0.2 | 1 | 2 | 1 | 4 |
| **Czech Republic** | 410 | low | 0.7 | 0.2 | 0.7 | 2 | 1 | 2 |

## Malta

| **Country of birth** | **Population (>15 yrs)** | **Anti-HCV endemicity** | **Anti-HCV prevalence** | | | **Estimated no. of CHC cases** | | |
| --- | --- | --- | --- | --- | --- | --- | --- | --- |
|  |  |  | **%** | **Lower limit** | **Upper limit** | **Cases** | **Lower** | **Upper** |
| **Australia** | 4,293 | high | 1.7 | 1.2 | 2.3 | 51 | 36 | 69 |
| **Egypt** | 438 | high | 15.7 | 13.9 | 17.5 | 48 | 43 | 54 |
| **Italy** | 1,419 | high | 4.4 | 1.6 | 7.3 | 44 | 16 | 73 |
| **Russian Federation** | 705 | high | 4.1 | 1.2 | 5.6 | 20 | 6 | 28 |
| **Nigeria** | 310 | high | 8.4 | 3.9 | 12.8 | 18 | 8 | 28 |
| **Canada** | 1,737 | high | 1.1 | 0.6 | 1.3 | 13 | 7 | 16 |
| **Romania** | 564 | high | 3.2 | 2.9 | 3.6 | 13 | 11 | 14 |
| **United States** | 1,131 | high | 1.3 | 1.2 | 2.4 | 10 | 10 | 19 |
| **Somalia** | 930 | high | 1 | 0.6 | 3.1 | 7 | 4 | 20 |
| **Ukraine** | 256 | high | 3.6 | 0.9 | 4.5 | 6 | 2 | 8 |
| **Bulgaria** | 797 | high | 1.1 | 0.3 | 2.4 | 6 | 2 | 13 |
| **Libya** | 730 | high | 1.2 | 1.2 | 2.3 | 6 | 6 | 12 |
| **Syria** | 244 | high | 3.1 | 2.5 | 3.9 | 5 | 4 | 7 |
| **Serbia** | 502 | high | 1.3 | 1.1 | 1.6 | 5 | 4 | 6 |
| **Pakistan** | 111 | high | 5 | 4.4 | 5.5 | 4 | 3 | 4 |
| **Ivory Coast** | 92 | high | 5.3 | 2.9 | 9.1 | 3 | 2 | 6 |
| **China** | 361 | high | 1.3 | 0.4 | 2 | 3 | 1 | 5 |
| **Eritrea** | 441 | high | 1 | 0.6 | 3.1 | 3 | 2 | 10 |
| **Tunisia** | 301 | high | 1.3 | 0.3 | 2.5 | 3 | 1 | 5 |
| **Morocco** | 213 | high | 1.6 | 0.6 | 1.9 | 2 | 1 | 3 |
| **South Africa** | 158 | high | 1.7 | 1 | 2.5 | 2 | 1 | 3 |
| **Ireland** | 241 | high | 1.1 | 0.7 | 1.6 | 2 | 1 | 3 |
| **Iraq** | 81 | high | 3.2 | 0.3 | 3.2 | 2 | 0 | 2 |
| **Thailand** | 95 | high | 2.7 | 1.8 | 3.7 | 2 | 1 | 2 |
| **Poland** | 230 | high | 1.1 | 0.6 | 1.9 | 2 | 1 | 3 |
| **Ethiopia** | 185 | high | 1.3 | 0.7 | 5.8 | 2 | 1 | 8 |
| **Spain** | 130 | high | 1.7 | 0.4 | 2.6 | 2 | 0 | 2 |
| **Sudan** | 218 | high | 1 | 0.6 | 3.1 | 2 | 1 | 5 |
| **Turkey** | 202 | high | 1 | 0.7 | 1.1 | 1 | 1 | 2 |
| **Bosnia and Herzegovina** | 153 | high | 1.3 | 1.1 | 1.6 | 1 | 1 | 2 |
| **Latvia** | 70 | high | 2.4 | 1.7 | 3.3 | 1 | 1 | 2 |
| **Albania** | 69 | high | 2.4 | 2 | 2.8 | 1 | 1 | 1 |
| **Switzerland** | 97 | high | 1.5 | 0.7 | 1.8 | 1 | 0 | 1 |
| **Brazil** | 89 | high | 1.6 | 1.1 | 1.6 | 1 | 1 | 1 |
| **Greece** | 74 | high | 1.9 | 0.5 | 2.6 | 1 | 0 | 1 |
| **Slovakia** | 95 | high | 1.4 | 0.9 | 2 | 1 | 1 | 1 |
| **Croatia** | 71 | high | 1.3 | 1.1 | 1.6 | 1 | 1 | 1 |
| **United Kingdom** | 9,977 | low | 0.6 | 0.4 | 1.2 | 42 | 28 | 84 |
| **Germany** | 886 | low | 0.5 | 0.3 | 0.9 | 3 | 2 | 6 |
| **Philippines** | 438 | low | 0.9 | 0.3 | 2 | 3 | 1 | 6 |
| **France** | 366 | low | 0.7 | 0.5 | 0.8 | 2 | 1 | 2 |
| **Sweden** | 399 | low | 0.6 | 0.5 | 0.7 | 2 | 1 | 2 |
| **India** | 250 | low | 0.8 | 0.4 | 1 | 1 | 1 | 2 |
| **Belgium** | 135 | low | 0.9 | 0.1 | 1.2 | 1 | 0 | 1 |
| **Hungary** | 132 | low | 0.8 | 0.4 | 2.7 | 1 | 0 | 2 |
| **Denmark** | 110 | low | 0.7 | 0.5 | 0.7 | 1 | 0 | 1 |
| **Netherlands** | 378 | low | 0.2 | 0.1 | 0.4 | 1 | 0 | 1 |
| **Czech Republic** | 78 | low | 0.7 | 0.2 | 0.7 | 0 | 0 | 0 |
| **Austria** | 100 | low | 0.5 | 0.1 | 0.7 | 0 | 0 | 0 |
| **Finland** | 69 | low | 0.7 | 0.6 | 0.9 | 0 | 0 | 0 |

## The Netherlands

| **Country of birth** | **Population (>15 yrs)** | **Anti-HCV endemicity** | **Anti-HCV prevalence** | | | **Estimated no. of CHC cases** | | |
| --- | --- | --- | --- | --- | --- | --- | --- | --- |
|  |  |  | **%** | **Lower limit** | **Upper limit** | **Cases** | **Lower** | **Upper** |
| **Morocco** | 166,178 | high | 1.6 | 0.6 | 1.9 | 1,861 | 698 | 2,210 |
| **Turkey** | 193,763 | high | 1 | 0.7 | 1.1 | 1,356 | 949 | 1,492 |
| **Egypt** | 11,341 | high | 15.7 | 13.9 | 17.5 | 1,246 | 1,103 | 1,389 |
| **Former Soviet Union** | 38,194 | high | 3.3 | 1.6 | 4.5 | 882 | 428 | 1,203 |
| **Iraq** | 38,494 | high | 3.2 | 0.3 | 3.2 | 862 | 81 | 862 |
| **Italy** | 21,197 | high | 4.4 | 1.6 | 7.3 | 653 | 237 | 1,083 |
| **Poland** | 78,845 | high | 1.1 | 0.6 | 1.9 | 607 | 331 | 1,049 |
| **Ghana** | 13,352 | high | 5.3 | 2.9 | 9.1 | 495 | 271 | 851 |
| **China** | 52,993 | high | 1.3 | 0.4 | 2 | 482 | 148 | 742 |
| **Former Yugoslavia** | 50,357 | high | 1.3 | 1.1 | 1.6 | 458 | 388 | 564 |
| **Cape Verde** | 11,571 | high | 5.3 | 2.9 | 9.1 | 429 | 235 | 737 |
| **Pakistan** | 10,949 | high | 5 | 4.4 | 5.5 | 383 | 337 | 422 |
| **Nigeria** | 6,348 | high | 8.4 | 3.9 | 12.8 | 373 | 173 | 569 |
| **Romania** | 13,046 | high | 3.2 | 2.9 | 3.6 | 292 | 265 | 329 |
| **Afghanistan** | 31,434 | high | 1.1 | 0.6 | 1.9 | 242 | 132 | 418 |
| **Spain** | 19,902 | high | 1.7 | 0.4 | 2.6 | 237 | 56 | 362 |
| **Thailand** | 11,943 | high | 2.7 | 1.8 | 3.7 | 226 | 150 | 309 |
| **United States** | 21,834 | high | 1.3 | 1.2 | 2.4 | 199 | 183 | 367 |
| **Portugal** | 14,583 | high | 1.8 | 0.5 | 2.9 | 184 | 51 | 296 |
| **Syria** | 7,338 | high | 3.1 | 2.5 | 3.9 | 159 | 128 | 200 |
| **Greece** | 11,882 | high | 1.9 | 0.5 | 2.6 | 158 | 42 | 216 |
| **Brazil** | 13,740 | high | 1.6 | 1.1 | 1.6 | 154 | 106 | 154 |
| **Angola** | 5,209 | high | 4.2 | 2.4 | 9.2 | 153 | 88 | 335 |
| **Somalia** | 20,872 | high | 1 | 0.6 | 3.1 | 146 | 88 | 453 |
| **South Africa** | 11,936 | high | 1.7 | 1 | 2.5 | 142 | 84 | 209 |
| **Bulgaria** | 16,203 | high | 1.1 | 0.3 | 2.4 | 125 | 34 | 272 |
| **Australia** | 9,366 | high | 1.7 | 1.2 | 2.3 | 111 | 79 | 151 |
| **Vietnam** | 12,225 | high | 1 | 0.8 | 1.8 | 86 | 68 | 154 |
| **Colombia** | 12,100 | high | 1 | 0.8 | 1.4 | 85 | 68 | 119 |
| **Czech Republic/Slovakia** | 8,688 | high | 1.3 | 1.1 | 1.6 | 79 | 67 | 97 |
| **Ethiopia** | 8,325 | high | 1.3 | 0.7 | 5.8 | 76 | 41 | 338 |
| **Sri Lanka** | 9,887 | high | 1 | 0.8 | 1.8 | 69 | 55 | 125 |
| **Canada** | 8,970 | high | 1.1 | 0.6 | 1.3 | 69 | 38 | 82 |
| **Israel** | 4,715 | high | 2 | 0.9 | 2 | 66 | 30 | 66 |
| **Switzerland** | 5,844 | high | 1.5 | 0.7 | 1.8 | 61 | 29 | 74 |
| **Japan** | 5,074 | high | 1.5 | 0.5 | 2.2 | 53 | 18 | 78 |
| **Suriname** | 181,917 | low | 0.8 | 0.2 | 1.3 | 1,019 | 255 | 1,655 |
| **Indonesia** | 131,261 | low | 0.8 | 0.4 | 2 | 735 | 368 | 1,838 |
| **Former Dutch Antilles** | 82,589 | low | 0.8 | 0.2 | 1.3 | 462 | 116 | 752 |
| **Germany** | 115,742 | low | 0.5 | 0.3 | 0.9 | 405 | 243 | 729 |
| **Belgium** | 44,096 | low | 0.9 | 0.1 | 1.2 | 278 | 31 | 370 |
| **United Kingdom** | 43,492 | low | 0.6 | 0.4 | 1.2 | 183 | 122 | 365 |
| **India** | 19,104 | low | 0.8 | 0.4 | 1 | 107 | 53 | 134 |
| **France** | 20,583 | low | 0.7 | 0.5 | 0.8 | 101 | 72 | 115 |
| **Iran** | 27,261 | low | 0.5 | 0.2 | 1 | 95 | 38 | 191 |
| **Philippines** | 10,851 | low | 0.9 | 0.3 | 2 | 68 | 23 | 152 |
| **Hungary** | 10,978 | low | 0.8 | 0.4 | 2.7 | 61 | 31 | 207 |
| **Dominican Rep,** | 7,942 | low | 0.8 | 0.2 | 1.3 | 44 | 11 | 72 |
| **South Korea** | 6,239 | low | 0.8 | 0.2 | 2.1 | 35 | 9 | 92 |
| **Austria** | 6,174 | low | 0.5 | 0.1 | 0.7 | 22 | 4 | 30 |

## Norway

| **Country of birth** | **Population (>15 yrs)** | **Anti-HCV endemicity** | **Anti-HCV prevalence** | | | **Estimated no. of CHC cases** | | |
| --- | --- | --- | --- | --- | --- | --- | --- | --- |
|  |  |  | **%** | **Lower limit** | **Upper limit** | **Cases** | **Lower** | **Upper** |
| **Pakistan** | 17,780 | high | 5 | 4.4 | 5.5 | 622 | 548 | 685 |
| **Poland** | 69,545 | high | 1.1 | 0.6 | 1.9 | 535 | 292 | 925 |
| **Lithuania** | 25,226 | high | 2.9 | 0.7 | 3 | 512 | 124 | 530 |
| **Iraq** | 20,250 | high | 3.2 | 0.3 | 3.2 | 454 | 43 | 454 |
| **Russia** | 14,250 | high | 4.1 | 1.2 | 5.6 | 409 | 120 | 559 |
| **Thailand** | 14,039 | high | 2.7 | 1.8 | 3.7 | 265 | 177 | 364 |
| **Romania** | 7,371 | high | 3.2 | 2.9 | 3.6 | 165 | 150 | 186 |
| **Somalia** | 20,449 | high | 1 | 0.6 | 3.1 | 143 | 86 | 444 |
| **United States** | 14,886 | high | 1.3 | 1.2 | 2.4 | 135 | 125 | 250 |
| **Latvia** | 7,078 | high | 2.4 | 1.7 | 3.3 | 119 | 84 | 164 |
| **Bosnia and Herzegovina** | 12,934 | high | 1.3 | 1.1 | 1.6 | 118 | 100 | 145 |
| **Vietnam** | 13,230 | high | 1 | 0.8 | 1.8 | 93 | 74 | 167 |
| **Estonia** | 3,969 | high | 3.3 | 1.6 | 4.5 | 92 | 44 | 125 |
| **Italy** | 2,839 | high | 4.4 | 1.6 | 7.3 | 87 | 32 | 145 |
| **Kosovo** | 9,211 | high | 1.3 | 1.1 | 1.6 | 84 | 71 | 103 |
| **Ukraine** | 3,253 | high | 3.6 | 0.9 | 4.5 | 82 | 20 | 102 |
| **Afghanistan** | 10,545 | high | 1.1 | 0.6 | 1.9 | 81 | 44 | 140 |
| **China** | 8,703 | high | 1.3 | 0.4 | 2 | 79 | 24 | 122 |
| **Turkey** | 10,523 | high | 1 | 0.7 | 1.1 | 74 | 52 | 81 |
| **Sri Lanka** | 8,875 | high | 1 | 0.8 | 1.8 | 62 | 50 | 112 |
| **Eritrea** | 8,359 | high | 1 | 0.6 | 3.1 | 59 | 35 | 181 |
| **Morocco** | 5,093 | high | 1.6 | 0.6 | 1.9 | 57 | 21 | 68 |
| **Chile** | 6,267 | high | 1.2 | 0.5 | 2.1 | 53 | 22 | 92 |
| **Palestine** | 2,315 | high | 3.1 | 2.5 | 3.9 | 50 | 41 | 63 |
| **Ethiopia** | 5,457 | high | 1.3 | 0.7 | 5.8 | 50 | 27 | 222 |
| **Spain** | 3,872 | high | 1.7 | 0.4 | 2.6 | 46 | 11 | 70 |
| **Brazil** | 3,847 | high | 1.6 | 1.1 | 1.6 | 43 | 30 | 43 |
| **Myanmar (Burma)** | 2,692 | high | 1.7 | 1 | 2.7 | 32 | 19 | 51 |
| **Slovakia** | 3,056 | high | 1.4 | 0.9 | 2 | 30 | 19 | 43 |
| **Colombia** | 4,074 | high | 1 | 0.8 | 1.4 | 29 | 23 | 40 |
| **Bulgaria** | 3,573 | high | 1.1 | 0.3 | 2.4 | 28 | 8 | 60 |
| **Croatia** | 2,898 | high | 1.3 | 1.1 | 1.6 | 26 | 22 | 32 |
| **Serbia** | 2,830 | high | 1.3 | 1.1 | 1.6 | 26 | 22 | 32 |
| **Portugal** | 1,751 | high | 1.8 | 0.5 | 2.9 | 22 | 6 | 36 |
| **Canada** | 2,707 | high | 1.1 | 0.6 | 1.3 | 21 | 11 | 25 |
| **Macedonia** | 2,271 | high | 1.3 | 1.1 | 1.6 | 21 | 17 | 25 |
| **Switzerland** | 1,859 | high | 1.5 | 0.7 | 1.8 | 20 | 9 | 23 |
| **Sweden** | 45,491 | low | 0.6 | 0.5 | 0.7 | 191 | 159 | 223 |
| **Denmark** | 22,035 | low | 0.7 | 0.5 | 0.7 | 108 | 77 | 108 |
| **Philippines** | 16,169 | low | 0.9 | 0.3 | 2 | 102 | 34 | 226 |
| **Germany** | 25,092 | low | 0.5 | 0.3 | 0.9 | 88 | 53 | 158 |
| **United Kingdom** | 16,753 | low | 0.6 | 0.4 | 1.2 | 70 | 47 | 141 |
| **India** | 9,114 | low | 0.8 | 0.4 | 1 | 51 | 26 | 64 |
| **Iran** | 14,410 | low | 0.5 | 0.2 | 1 | 50 | 20 | 101 |
| **Iceland** | 6,237 | low | 0.9 | 0.7 | 1.5 | 39 | 31 | 65 |
| **South Korea** | 6,115 | low | 0.8 | 0.2 | 2.1 | 34 | 9 | 90 |
| **Finland** | 6,334 | low | 0.7 | 0.6 | 0.9 | 31 | 27 | 40 |
| **France** | 5,098 | low | 0.7 | 0.5 | 0.8 | 25 | 18 | 29 |
| **Hungary** | 2,800 | low | 0.8 | 0.4 | 2.7 | 16 | 8 | 53 |
| **Netherlands** | 7,002 | low | 0.2 | 0.1 | 0.4 | 10 | 5 | 20 |

## Poland

| **Country of birth** | **Population (>15 yrs)** | **Anti-HCV endemicity** | **Anti-HCV prevalence** | | | **Estimated no. of CHC cases** | | |
| --- | --- | --- | --- | --- | --- | --- | --- | --- |
|  |  |  | **%** | **Lower limit** | **Upper limit** | **Cases** | **Lower** | **Upper** |
| **Ukraine** | 226,887 | high | 3.6 | 0.9 | 4.5 | 5,718 | 1,429 | 7,147 |
| **Russia** | 40,808 | high | 4.1 | 1.2 | 5.6 | 1,171 | 343 | 1,600 |
| **Lithuania** | 55,493 | high | 2.9 | 0.7 | 3 | 1,127 | 272 | 1,165 |
| **Belarus** | 83,480 | high | 1.3 | 0.9 | 2.9 | 760 | 526 | 1,695 |
| **Kazakhstan** | 4,717 | high | 3.3 | 1 | 6.7 | 109 | 33 | 221 |
| **Italy** | 3,414 | high | 4.4 | 1.6 | 7.3 | 105 | 38 | 174 |
| **Armenia** | 2,349 | high | 5.4 | 3.5 | 6.8 | 89 | 58 | 112 |
| **Romania** | 2,606 | high | 3.2 | 2.9 | 3.6 | 58 | 53 | 66 |
| **United States** | 6,117 | high | 1.3 | 1.2 | 2.4 | 56 | 51 | 103 |
| **Egypt** | 480 | high | 15.7 | 13.9 | 17.5 | 53 | 47 | 59 |
| **Uzbekistan** | 509 | high | 11.3 | 6.4 | 13.1 | 40 | 23 | 47 |
| **Bosnia and Herzegovina** | 3,803 | high | 1.3 | 1.1 | 1.6 | 35 | 29 | 43 |
| **Latvia** | 1,683 | high | 2.4 | 1.7 | 3.3 | 28 | 20 | 39 |
| **Mongolia** | 355 | high | 10.8 | 8.7 | 15.6 | 27 | 22 | 39 |
| **Nigeria** | 449 | high | 8.4 | 3.9 | 12.8 | 26 | 12 | 40 |
| **Greece** | 1,753 | high | 1.9 | 0.5 | 2.6 | 23 | 6 | 32 |
| **Vietnam** | 3,055 | high | 1 | 0.8 | 1.8 | 21 | 17 | 38 |
| **Georgia** | 365 | high | 6.7 | 5.6 | 7.3 | 17 | 14 | 19 |
| **Moldova** | 620 | high | 3.3 | 1.6 | 4.5 | 14 | 7 | 20 |
| **Canada** | 1,833 | high | 1.1 | 0.6 | 1.3 | 14 | 8 | 17 |
| **Bulgaria** | 1,828 | high | 1.1 | 0.3 | 2.4 | 14 | 4 | 31 |
| **Syria** | 641 | high | 3.1 | 2.5 | 3.9 | 14 | 11 | 17 |
| **Slovakia** | 1,335 | high | 1.4 | 0.9 | 2 | 13 | 8 | 19 |
| **Spain** | 996 | high | 1.7 | 0.4 | 2.6 | 12 | 3 | 18 |
| **China** | 1,139 | high | 1.3 | 0.4 | 2 | 10 | 3 | 16 |
| **Iraq** | 445 | high | 3.2 | 0.3 | 3.2 | 10 | 1 | 10 |
| **Serbia** | 996 | high | 1.3 | 1.1 | 1.6 | 9 | 8 | 11 |
| **Lebanon** | 416 | high | 3.1 | 2.5 | 3.9 | 9 | 7 | 11 |
| **Australia** | 713 | high | 1.7 | 1.2 | 2.3 | 8 | 6 | 11 |
| **Croatia** | 800 | high | 1.3 | 1.1 | 1.6 | 7 | 6 | 9 |
| **Turkey** | 910 | high | 1 | 0.7 | 1.1 | 6 | 4 | 7 |
| **Algeria** | 618 | high | 1.4 | 0.2 | 2.5 | 6 | 1 | 11 |
| **Switzerland** | 521 | high | 1.5 | 0.7 | 1.8 | 5 | 3 | 7 |
| **Japan** | 418 | high | 1.5 | 0.5 | 2.2 | 4 | 1 | 6 |
| **Tunisia** | 470 | high | 1.3 | 0.3 | 2.5 | 4 | 1 | 8 |
| **South Africa** | 344 | high | 1.7 | 1 | 2.5 | 4 | 2 | 6 |
| **Morocco** | 357 | high | 1.6 | 0.6 | 1.9 | 4 | 1 | 5 |
| **Germany** | 69,682 | low | 0.5 | 0.3 | 0.9 | 244 | 146 | 439 |
| **France** | 24,875 | low | 0.7 | 0.5 | 0.8 | 122 | 87 | 139 |
| **Czech Republic** | 4,683 | low | 0.7 | 0.2 | 0.7 | 23 | 7 | 23 |
| **United Kingdom** | 3,374 | low | 0.6 | 0.4 | 1.2 | 14 | 9 | 28 |
| **Belgium** | 2,079 | low | 0.9 | 0.1 | 1.2 | 13 | 1 | 17 |
| **Austria** | 2,826 | low | 0.5 | 0.1 | 0.7 | 10 | 2 | 14 |
| **Hungary** | 1,348 | low | 0.8 | 0.4 | 2.7 | 8 | 4 | 25 |
| **India** | 871 | low | 0.8 | 0.4 | 1 | 5 | 2 | 6 |
| **Sweden** | 683 | low | 0.6 | 0.5 | 0.7 | 3 | 2 | 3 |
| **Denmark** | 539 | low | 0.7 | 0.5 | 0.7 | 3 | 2 | 3 |
| **Norway** | 390 | low | 0.7 | 0.6 | 0.9 | 2 | 2 | 2 |
| **Netherlands** | 1,184 | low | 0.2 | 0.1 | 0.4 | 2 | 1 | 3 |

## Portugal

| **Country of birth** | **Population (>15 yrs)** | **Anti-HCV endemicity** | **Anti-HCV prevalence** | | | **Estimated no. of CHC cases** | | |
| --- | --- | --- | --- | --- | --- | --- | --- | --- |
|  |  |  | **%** | **Lower limit** | **Upper limit** | **Cases** | **Lower** | **Upper** |
| **Angola** | 159,008 | high | 4.2 | 2.4 | 9.2 | 4,675 | 2,671 | 10,240 |
| **Cape Verde** | 57,903 | high | 5.3 | 2.9 | 9.1 | 2,148 | 1,175 | 3,688 |
| **Brazil** | 125,547 | high | 1.6 | 1.1 | 1.6 | 1,406 | 967 | 1,406 |
| **Guinea-Bissau** | 27,196 | high | 5.3 | 2.9 | 9.1 | 1,009 | 552 | 1,732 |
| **Ukraine** | 30,355 | high | 3.6 | 0.9 | 4.5 | 765 | 191 | 956 |
| **Sao Tome and Principe** | 17,115 | high | 5.3 | 2.9 | 9.1 | 635 | 347 | 1,090 |
| **Mozambique** | 72,308 | high | 1 | 0.6 | 3.1 | 506 | 304 | 1,569 |
| **Romania** | 20,885 | high | 3.2 | 2.9 | 3.6 | 468 | 424 | 526 |
| **Moldova** | 12,746 | high | 3.3 | 1.6 | 4.5 | 294 | 143 | 401 |
| **Venezuela** | 24,133 | high | 1.5 | 0.3 | 2.6 | 253 | 51 | 439 |
| **Spain** | 13,718 | high | 1.7 | 0.4 | 2.6 | 163 | 38 | 250 |
| **Russia** | 5,504 | high | 4.1 | 1.2 | 5.6 | 158 | 46 | 216 |
| **South Africa** | 10,977 | high | 1.7 | 1 | 2.5 | 131 | 77 | 192 |
| **Switzerland** | 11,666 | high | 1.5 | 0.7 | 1.8 | 122 | 57 | 147 |
| **China** | 10,106 | high | 1.3 | 0.4 | 2 | 92 | 28 | 141 |
| **Italy** | 2,530 | high | 4.4 | 1.6 | 7.3 | 78 | 28 | 129 |
| **Congo** | 2,300 | high | 4.2 | 2.4 | 9.2 | 68 | 39 | 148 |
| **Pakistan** | 1,715 | high | 5 | 4.4 | 5.5 | 60 | 53 | 66 |
| **United States** | 6,175 | high | 1.3 | 1.2 | 2.4 | 56 | 52 | 104 |
| **Canada** | 6,958 | high | 1.1 | 0.6 | 1.3 | 54 | 29 | 63 |
| **Uzbekistan** | 620 | high | 11.3 | 6.4 | 13.1 | 49 | 28 | 57 |
| **Senegal** | 1,231 | high | 5.3 | 2.9 | 9.1 | 46 | 25 | 78 |
| **Bulgaria** | 4,858 | high | 1.1 | 0.3 | 2.4 | 37 | 10 | 82 |
| **Georgia** | 738 | high | 6.7 | 5.6 | 7.3 | 35 | 29 | 38 |
| **Morocco** | 2,247 | high | 1.6 | 0.6 | 1.9 | 25 | 9 | 30 |
| **DR Congo** | 825 | high | 4.3 | 3.2 | 13.7 | 25 | 18 | 79 |
| **Guinea** | 644 | high | 5.3 | 2.9 | 9.1 | 24 | 13 | 41 |
| **Zimbabwe** | 1,463 | high | 1.6 | 1 | 9.1 | 16 | 10 | 93 |
| **Kazakhstan** | 649 | high | 3.3 | 1 | 6.7 | 15 | 5 | 30 |
| **Argentina** | 1,293 | high | 1.5 | 0.5 | 2.5 | 14 | 5 | 23 |
| **Australia** | 1,138 | high | 1.7 | 1.2 | 2.3 | 14 | 10 | 18 |
| **Timor-Leste** | 1,753 | high | 1 | 0.8 | 1.8 | 12 | 10 | 22 |
| **Ireland** | 1,387 | high | 1.1 | 0.7 | 1.6 | 11 | 7 | 16 |
| **Thailand** | 475 | high | 2.7 | 1.8 | 3.7 | 9 | 6 | 12 |
| **Bangladesh** | 969 | high | 1.3 | 0.2 | 2.2 | 9 | 1 | 15 |
| **Nepal** | 945 | high | 1.1 | 0.7 | 1.5 | 7 | 5 | 10 |
| **Poland** | 941 | high | 1.1 | 0.6 | 1.9 | 7 | 4 | 13 |
| **Colombia** | 687 | high | 1 | 0.8 | 1.4 | 5 | 4 | 7 |
| **Belarus** | 504 | high | 1.3 | 0.9 | 2.9 | 5 | 3 | 10 |
| **France** | 88,825 | low | 0.7 | 0.5 | 0.8 | 435 | 311 | 497 |
| **Germany** | 25,009 | low | 0.5 | 0.3 | 0.9 | 88 | 53 | 158 |
| **United Kingdom** | 15,695 | low | 0.6 | 0.4 | 1.2 | 66 | 44 | 132 |
| **India** | 7,732 | low | 0.8 | 0.4 | 1 | 43 | 22 | 54 |
| **Luxembourg** | 3,150 | low | 0.9 | 0.6 | 0.9 | 20 | 13 | 20 |
| **Belgium** | 2,943 | low | 0.9 | 0.1 | 1.2 | 19 | 2 | 25 |
| **Cuba** | 947 | low | 0.8 | 0.2 | 1.3 | 5 | 1 | 9 |
| **Netherlands** | 3,655 | low | 0.2 | 0.1 | 0.4 | 5 | 3 | 10 |
| **Sweden** | 763 | low | 0.6 | 0.5 | 0.7 | 3 | 3 | 4 |
| **Philippines** | 446 | low | 0.9 | 0.3 | 2 | 3 | 1 | 6 |
| **Austria** | 471 | low | 0.5 | 0.1 | 0.7 | 2 | 0 | 2 |

## Romania

| **Country of birth** | **Population (>15 yrs)** | **Anti-HCV endemicity** | **Anti-HCV prevalence** | | | **Estimated no. of CHC cases** | | |
| --- | --- | --- | --- | --- | --- | --- | --- | --- |
|  |  |  | **%** | **Lower limit** | **Upper limit** | **Cases** | **Lower** | **Upper** |
| **Moldova** | 56,435 | high | 3.3 | 1.6 | 4.5 | 1,304 | 632 | 1,778 |
| **Ukraine** | 8,589 | high | 3.6 | 0.9 | 4.5 | 216 | 54 | 271 |
| **Russia** | 4,893 | high | 4.1 | 1.2 | 5.6 | 140 | 41 | 192 |
| **Italy** | 3,383 | high | 4.4 | 1.6 | 7.3 | 104 | 38 | 173 |
| **Bulgaria** | 11,109 | high | 1.1 | 0.3 | 2.4 | 86 | 23 | 187 |
| **Syria** | 1,985 | high | 3.1 | 2.5 | 3.9 | 43 | 35 | 54 |
| **Turkey** | 4,129 | high | 1 | 0.7 | 1.1 | 29 | 20 | 32 |
| **Greece** | 2,145 | high | 1.9 | 0.5 | 2.6 | 29 | 8 | 39 |
| **China** | 2,882 | high | 1.3 | 0.4 | 2 | 26 | 8 | 40 |
| **Iraq** | 1,041 | high | 3.2 | 0.3 | 3.2 | 23 | 2 | 23 |
| **Israel** | 1,219 | high | 2 | 0.9 | 2 | 17 | 8 | 17 |
| **Serbia** | 1,278 | high | 1.3 | 1.1 | 1.6 | 12 | 10 | 14 |
| **Tunisia** | 1,013 | high | 1.3 | 0.3 | 2.5 | 9 | 2 | 18 |
| **United States** | 862 | high | 1.3 | 1.2 | 2.4 | 8 | 7 | 14 |
| **Spain** | 533 | high | 1.7 | 0.4 | 2.6 | 6 | 1 | 10 |
| **Nigeria** | 90 | high | 8.4 | 3.9 | 12.8 | 5 | 2 | 8 |
| **Pakistan** | 141 | high | 5 | 4.4 | 5.5 | 5 | 4 | 5 |
| **Mali** | 107 | high | 5.3 | 2.9 | 9.1 | 4 | 2 | 7 |
| **Ghana** | 104 | high | 5.3 | 2.9 | 9.1 | 4 | 2 | 7 |
| **Egypt** | 28 | high | 15.7 | 13.9 | 17.5 | 3 | 3 | 3 |
| **Guinea** | 73 | high | 5.3 | 2.9 | 9.1 | 3 | 1 | 5 |
| **Cameroon** | 27 | high | 11.6 | 4.3 | 29.7 | 2 | 1 | 6 |
| **Lebanon** | 98 | high | 3.1 | 2.5 | 3.9 | 2 | 2 | 3 |
| **Albania** | 102 | high | 2.4 | 2 | 2.8 | 2 | 1 | 2 |
| **Senegal** | 35 | high | 5.3 | 2.9 | 9.1 | 1 | 1 | 2 |
| **Equatorial Guinea** | 44 | high | 4.2 | 2.4 | 9.2 | 1 | 1 | 3 |
| **Bangladesh** | 117 | high | 1.3 | 0.2 | 2.2 | 1 | 0 | 2 |
| **Portugal** | 68 | high | 1.8 | 0.5 | 2.9 | 1 | 0 | 1 |
| **Algeria** | 77 | high | 1.4 | 0.2 | 2.5 | 1 | 0 | 1 |
| **Poland** | 87 | high | 1.1 | 0.6 | 1.9 | 1 | 0 | 1 |
| **Mauritania** | 48 | high | 1.9 | 1.1 | 10.7 | 1 | 0 | 4 |
| **Mexico** | 50 | high | 1.4 | 1.1 | 1.6 | 0 | 0 | 1 |
| **Morocco** | 31 | high | 1.6 | 0.6 | 1.9 | 0 | 0 | 0 |
| **Vietnam** | 31 | high | 1 | 0.8 | 1.8 | 0 | 0 | 0 |
| **Hungary** | 3,729 | low | 0.8 | 0.4 | 2.7 | 21 | 10 | 70 |
| **Germany** | 2,511 | low | 0.5 | 0.3 | 0.9 | 9 | 5 | 16 |
| **France** | 1,700 | low | 0.7 | 0.5 | 0.8 | 8 | 6 | 10 |
| **Iran** | 1,034 | low | 0.5 | 0.2 | 1 | 4 | 1 | 7 |
| **United Kingdom** | 706 | low | 0.6 | 0.4 | 1.2 | 3 | 2 | 6 |
| **Austria** | 106 | low | 0.5 | 0.1 | 0.7 | 0 | 0 | 1 |
| **Belgium** | 49 | low | 0.9 | 0.1 | 1.2 | 0 | 0 | 0 |
| **India** | 39 | low | 0.8 | 0.4 | 1 | 0 | 0 | 0 |
| **Cuba** | 37 | low | 0.8 | 0.2 | 1.3 | 0 | 0 | 0 |
| **Sweden** | 49 | low | 0.6 | 0.5 | 0.7 | 0 | 0 | 0 |
| **Denmark** | 39 | low | 0.7 | 0.5 | 0.7 | 0 | 0 | 0 |
| **Czech Republic** | 38 | low | 0.7 | 0.2 | 0.7 | 0 | 0 | 0 |
| **Dominican Republic** | 33 | low | 0.8 | 0.2 | 1.3 | 0 | 0 | 0 |
| **Netherlands** | 78 | low | 0.2 | 0.1 | 0.4 | 0 | 0 | 0 |

## Slovakia

| **Country of birth** | **Population (>15 yrs)** | **Anti-HCV endemicity** | **Anti-HCV prevalence** | | | **Estimated no. of CHC cases** | | |
| --- | --- | --- | --- | --- | --- | --- | --- | --- |
|  |  |  | **%** | **Lower limit** | **Upper limit** | **Cases** | **Lower** | **Upper** |
| **Ukraine** | 9,589 | high | 3.6 | 0.9 | 4.5 | 242 | 60 | 302 |
| **Romania** | 5,189 | high | 3.2 | 2.9 | 3.6 | 116 | 105 | 131 |
| **Russia** | 2,208 | high | 4.1 | 1.2 | 5.6 | 63 | 19 | 87 |
| **Poland** | 4,437 | high | 1.1 | 0.6 | 1.9 | 34 | 19 | 59 |
| **Italy** | 878 | high | 4.4 | 1.6 | 7.3 | 27 | 10 | 45 |
| **Serbia** | 1,502 | high | 1.3 | 1.1 | 1.6 | 14 | 12 | 17 |
| **Egypt** | 109 | high | 15.7 | 13.9 | 17.5 | 12 | 11 | 13 |
| **Vietnam** | 1,521 | high | 1 | 0.8 | 1.8 | 11 | 9 | 19 |
| **Bulgaria** | 1,280 | high | 1.1 | 0.3 | 2.4 | 10 | 3 | 22 |
| **United States** | 777 | high | 1.3 | 1.2 | 2.4 | 7 | 7 | 13 |
| **China** | 563 | high | 1.3 | 0.4 | 2 | 5 | 2 | 8 |
| **Chad** | 137 | high | 5.3 | 2.9 | 9.1 | 5 | 3 | 9 |
| **Croatia** | 546 | high | 1.3 | 1.1 | 1.6 | 5 | 4 | 6 |
| **Armenia** | 108 | high | 5.4 | 3.5 | 6.8 | 4 | 3 | 5 |
| **Syria** | 163 | high | 3.1 | 2.5 | 3.9 | 4 | 3 | 4 |
| **Kazakhstan** | 143 | high | 3.3 | 1 | 6.7 | 3 | 1 | 7 |
| **Bosnia and Herzegovina** | 311 | high | 1.3 | 1.1 | 1.6 | 3 | 2 | 3 |
| **Spain** | 217 | high | 1.7 | 0.4 | 2.6 | 3 | 1 | 4 |
| **Taiwan** | 69 | high | 4.4 | 2.5 | 6.3 | 2 | 1 | 3 |
| **Greece** | 136 | high | 1.9 | 0.5 | 2.6 | 2 | 0 | 2 |
| **Switzerland** | 154 | high | 1.5 | 0.7 | 1.8 | 2 | 1 | 2 |
| **Lithuania** | 78 | high | 2.9 | 0.7 | 3 | 2 | 0 | 2 |
| **Moldova** | 67 | high | 3.3 | 1.6 | 4.5 | 2 | 1 | 2 |
| **Slovenia** | 169 | high | 1.3 | 1.1 | 1.6 | 2 | 1 | 2 |
| **Belarus** | 153 | high | 1.3 | 0.9 | 2.9 | 1 | 1 | 3 |
| **Canada** | 177 | high | 1.1 | 0.6 | 1.3 | 1 | 1 | 2 |
| **Turkey** | 148 | high | 1 | 0.7 | 1.1 | 1 | 1 | 1 |
| **Australia** | 83 | high | 1.7 | 1.2 | 2.3 | 1 | 1 | 1 |
| **Afghanistan** | 125 | high | 1.1 | 0.6 | 1.9 | 1 | 1 | 2 |
| **Israel** | 63 | high | 2 | 0.9 | 2 | 1 | 0 | 1 |
| **Portugal** | 65 | high | 1.8 | 0.5 | 2.9 | 1 | 0 | 1 |
| **Tunisia** | 83 | high | 1.3 | 0.3 | 2.5 | 1 | 0 | 1 |
| **Argentina** | 65 | high | 1.5 | 0.5 | 2.5 | 1 | 0 | 1 |
| **Algeria** | 65 | high | 1.4 | 0.2 | 2.5 | 1 | 0 | 1 |
| **Ireland** | 82 | high | 1.1 | 0.7 | 1.6 | 1 | 0 | 1 |
| **Czech Republic** | 80,915 | low | 0.7 | 0.2 | 0.7 | 396 | 113 | 396 |
| **Hungary** | 16,219 | low | 0.8 | 0.4 | 2.7 | 91 | 45 | 307 |
| **France** | 1,977 | low | 0.7 | 0.5 | 0.8 | 10 | 7 | 11 |
| **Germany** | 1,702 | low | 0.5 | 0.3 | 0.9 | 6 | 4 | 11 |
| **Austria** | 1,324 | low | 0.5 | 0.1 | 0.7 | 5 | 1 | 6 |
| **United Kingdom** | 790 | low | 0.6 | 0.4 | 1.2 | 3 | 2 | 7 |
| **Belgium** | 346 | low | 0.9 | 0.1 | 1.2 | 2 | 0 | 3 |
| **South Korea** | 235 | low | 0.8 | 0.2 | 2.1 | 1 | 0 | 3 |
| **Norway** | 187 | low | 0.7 | 0.6 | 0.9 | 1 | 1 | 1 |
| **Cuba** | 103 | low | 0.8 | 0.2 | 1.3 | 1 | 0 | 1 |
| **India** | 93 | low | 0.8 | 0.4 | 1 | 1 | 0 | 1 |
| **Denmark** | 65 | low | 0.7 | 0.5 | 0.7 | 0 | 0 | 0 |
| **Sweden** | 73 | low | 0.6 | 0.5 | 0.7 | 0 | 0 | 0 |
| **Netherlands** | 201 | low | 0.2 | 0.1 | 0.4 | 0 | 0 | 1 |
| **Iran** | 69 | low | 0.5 | 0.2 | 1 | 0 | 0 | 0 |

## Slovenia

| **Country of birth** | **Population (>15 yrs)** | **Anti-HCV endemicity** | **Anti-HCV prevalence** | | | **Estimated no. of CHC cases** | | |
| --- | --- | --- | --- | --- | --- | --- | --- | --- |
|  |  |  | **%** | **Lower limit** | **Upper limit** | **Cases** | **Lower** | **Upper** |
| **Bosnia and Herzegovina** | 95,960 | high | 1.3 | 1.1 | 1.6 | 873 | 739 | 1,075 |
| **Croatia** | 47,670 | high | 1.3 | 1.1 | 1.6 | 434 | 367 | 534 |
| **Serbia** | 25,839 | high | 1.3 | 1.1 | 1.6 | 235 | 199 | 289 |
| **FYR Macedonia** | 13,641 | high | 1.3 | 1.1 | 1.6 | 124 | 105 | 153 |
| **Italy** | 2,989 | high | 4.4 | 1.6 | 7.3 | 92 | 33 | 153 |
| **Kosovo** | 9,190 | high | 1.3 | 1.1 | 1.6 | 84 | 71 | 103 |
| **Ukraine** | 1,458 | high | 3.6 | 0.9 | 4.5 | 37 | 9 | 46 |
| **Russia** | 1,221 | high | 4.1 | 1.2 | 5.6 | 35 | 10 | 48 |
| **Montenegro** | 2,780 | high | 1.3 | 1.1 | 1.6 | 25 | 21 | 31 |
| **Egypt** | 90 | high | 15.7 | 13.9 | 17.5 | 10 | 9 | 11 |
| **Switzerland** | 895 | high | 1.5 | 0.7 | 1.8 | 9 | 4 | 11 |
| **Bulgaria** | 998 | high | 1.1 | 0.3 | 2.4 | 8 | 2 | 17 |
| **Romania** | 336 | high | 3.2 | 2.9 | 3.6 | 8 | 7 | 8 |
| **China** | 783 | high | 1.3 | 0.4 | 2 | 7 | 2 | 11 |
| **Moldova** | 288 | high | 3.3 | 1.6 | 4.5 | 7 | 3 | 9 |
| **Spain** | 411 | high | 1.7 | 0.4 | 2.6 | 5 | 1 | 7 |
| **Slovakia** | 483 | high | 1.4 | 0.9 | 2 | 5 | 3 | 7 |
| **United States** | 488 | high | 1.3 | 1.2 | 2.4 | 4 | 4 | 8 |
| **Australia** | 343 | high | 1.7 | 1.2 | 2.3 | 4 | 3 | 6 |
| **Argentina** | 375 | high | 1.5 | 0.5 | 2.5 | 4 | 1 | 7 |
| **Thailand** | 191 | high | 2.7 | 1.8 | 3.7 | 4 | 2 | 5 |
| **Canada** | 372 | high | 1.1 | 0.6 | 1.3 | 3 | 2 | 3 |
| **Poland** | 363 | high | 1.1 | 0.6 | 1.9 | 3 | 2 | 5 |
| **Albania** | 136 | high | 2.4 | 2 | 2.8 | 2 | 2 | 3 |
| **Iraq** | 54 | high | 3.2 | 0.3 | 3.2 | 1 | 0 | 1 |
| **Jordan** | 54 | high | 3.1 | 2.5 | 3.9 | 1 | 1 | 1 |
| **Belarus** | 123 | high | 1.3 | 0.9 | 2.9 | 1 | 1 | 2 |
| **Greece** | 81 | high | 1.9 | 0.5 | 2.6 | 1 | 0 | 1 |
| **Kazakhstan** | 45 | high | 3.3 | 1 | 6.7 | 1 | 0 | 2 |
| **Brazil** | 86 | high | 1.6 | 1.1 | 1.6 | 1 | 1 | 1 |
| **Japan** | 60 | high | 1.5 | 0.5 | 2.2 | 1 | 0 | 1 |
| **Turkey** | 86 | high | 1 | 0.7 | 1.1 | 1 | 0 | 1 |
| **Venezuela** | 45 | high | 1.5 | 0.3 | 2.6 | 0 | 0 | 1 |
| **Tunisia** | 49 | high | 1.3 | 0.3 | 2.5 | 0 | 0 | 1 |
| **Mexico** | 43 | high | 1.4 | 1.1 | 1.6 | 0 | 0 | 0 |
| **Germany** | 7,199 | low | 0.5 | 0.3 | 0.9 | 25 | 15 | 45 |
| **Austria** | 2,589 | low | 0.5 | 0.1 | 0.7 | 9 | 2 | 13 |
| **France** | 1,138 | low | 0.7 | 0.5 | 0.8 | 6 | 4 | 6 |
| **United Kingdom** | 469 | low | 0.6 | 0.4 | 1.2 | 2 | 1 | 4 |
| **Hungary** | 348 | low | 0.8 | 0.4 | 2.7 | 2 | 1 | 7 |
| **Czech Republic** | 340 | low | 0.7 | 0.2 | 0.7 | 2 | 0 | 2 |
| **Belgium** | 239 | low | 0.9 | 0.1 | 1.2 | 2 | 0 | 2 |
| **Sweden** | 324 | low | 0.6 | 0.5 | 0.7 | 1 | 1 | 2 |
| **Dominican Republic** | 148 | low | 0.8 | 0.2 | 1.3 | 1 | 0 | 1 |
| **India** | 107 | low | 0.8 | 0.4 | 1 | 1 | 0 | 1 |
| **Philippines** | 84 | low | 0.9 | 0.3 | 2 | 1 | 0 | 1 |
| **Netherlands** | 300 | low | 0.2 | 0.1 | 0.4 | 0 | 0 | 1 |
| **Cuba** | 57 | low | 0.8 | 0.2 | 1.3 | 0 | 0 | 1 |
| **Iran** | 68 | low | 0.5 | 0.2 | 1 | 0 | 0 | 0 |
| **Denmark** | 43 | low | 0.7 | 0.5 | 0.7 | 0 | 0 | 0 |

## Spain

| **Country of birth** | **Population (>15 yrs)** | **Anti-HCV endemicity** | **Anti-HCV prevalence** | | | **Estimated no. of CHC cases** | | |
| --- | --- | --- | --- | --- | --- | --- | --- | --- |
|  |  |  | **%** | **Lower limit** | **Upper limit** | **Cases** | **Lower** | **Upper** |
| **Romania** | 660,391 | high | 3.2 | 2.9 | 3.6 | 14,793 | 13,406 | 16,642 |
| **Morocco** | 682,412 | high | 1.6 | 0.6 | 1.9 | 7,643 | 2,866 | 9,076 |
| **Italy** | 93,453 | high | 4.4 | 1.6 | 7.3 | 2,878 | 1,047 | 4,775 |
| **Argentina** | 248,101 | high | 1.5 | 0.5 | 2.5 | 2,605 | 868 | 4,342 |
| **Colombia** | 342,317 | high | 1 | 0.8 | 1.4 | 2,396 | 1,917 | 3,355 |
| **Pakistan** | 55,086 | high | 5 | 4.4 | 5.5 | 1,928 | 1,697 | 2,121 |
| **Nigeria** | 32,421 | high | 8.4 | 3.9 | 12.8 | 1,906 | 885 | 2,905 |
| **Ukraine** | 74,876 | high | 3.6 | 0.9 | 4.5 | 1,887 | 472 | 2,359 |
| **Senegal** | 50,722 | high | 5.3 | 2.9 | 9.1 | 1,882 | 1,030 | 3,231 |
| **Russia** | 58,889 | high | 4.1 | 1.2 | 5.6 | 1,690 | 495 | 2,308 |
| **Venezuela** | 146,585 | high | 1.5 | 0.3 | 2.6 | 1,539 | 308 | 2,668 |
| **Peru** | 180,891 | high | 1.2 | 0.4 | 1.6 | 1,519 | 506 | 2,026 |
| **Portugal** | 116,039 | high | 1.8 | 0.5 | 2.9 | 1,462 | 406 | 2,356 |
| **China** | 136,552 | high | 1.3 | 0.4 | 2 | 1,243 | 382 | 1,912 |
| **Brazil** | 103,686 | high | 1.6 | 1.1 | 1.6 | 1,161 | 798 | 1,161 |
| **Bulgaria** | 129,167 | high | 1.1 | 0.3 | 2.4 | 995 | 271 | 2,170 |
| **Mali** | 18,915 | high | 5.3 | 2.9 | 9.1 | 702 | 384 | 1,205 |
| **Switzerland** | 59,965 | high | 1.5 | 0.7 | 1.8 | 630 | 294 | 756 |
| **Uruguay** | 72,858 | high | 1.2 | 0.5 | 2.1 | 612 | 255 | 1,071 |
| **Paraguay** | 66,790 | high | 1.2 | 0.9 | 1.2 | 561 | 421 | 561 |
| **Equatorial Guinea** | 18,704 | high | 4.2 | 2.4 | 9.2 | 550 | 314 | 1,205 |
| **Algeria** | 53,901 | high | 1.4 | 0.2 | 2.5 | 528 | 75 | 943 |
| **Poland** | 64,791 | high | 1.1 | 0.6 | 1.9 | 499 | 272 | 862 |
| **Chile** | 55,817 | high | 1.2 | 0.5 | 2.1 | 469 | 195 | 821 |
| **Ghana** | 12,542 | high | 5.3 | 2.9 | 9.1 | 465 | 255 | 799 |
| **Mexico** | 45,422 | high | 1.4 | 1.1 | 1.6 | 445 | 350 | 509 |
| **Guinea** | 9,908 | high | 5.3 | 2.9 | 9.1 | 368 | 201 | 631 |
| **Moldova** | 15,543 | high | 3.3 | 1.6 | 4.5 | 359 | 174 | 490 |
| **Lithuania** | 16,787 | high | 2.9 | 0.7 | 3 | 341 | 82 | 353 |
| **United States** | 32,892 | high | 1.3 | 1.2 | 2.4 | 299 | 276 | 553 |
| **Gambia** | 16,961 | high | 2.1 | 1.4 | 2.9 | 249 | 166 | 344 |
| **Honduras** | 33,924 | high | 1 | 0.8 | 1.4 | 237 | 190 | 332 |
| **Nicaragua** | 17,869 | high | 1 | 0.8 | 1.4 | 125 | 100 | 175 |
| **Ireland** | 13,533 | high | 1.1 | 0.7 | 1.6 | 104 | 66 | 152 |
| **Bangladesh** | 10,214 | high | 1.3 | 0.2 | 2.2 | 93 | 14 | 157 |
| **Ecuador** | 419,410 | low | 0.9 | 0.4 | 1.3 | 2,642 | 1,174 | 3,817 |
| **United Kingdom** | 305,274 | low | 0.6 | 0.4 | 1.2 | 1,282 | 855 | 2,564 |
| **Bolivia** | 157,885 | low | 0.9 | 0.4 | 1.3 | 995 | 442 | 1,437 |
| **France** | 200,276 | low | 0.7 | 0.5 | 0.8 | 981 | 701 | 1,122 |
| **Dominican Republic** | 141,154 | low | 0.8 | 0.2 | 1.3 | 790 | 198 | 1,285 |
| **Germany** | 202,092 | low | 0.5 | 0.3 | 0.9 | 707 | 424 | 1,273 |
| **Cuba** | 118,838 | low | 0.8 | 0.2 | 1.3 | 665 | 166 | 1,081 |
| **Belgium** | 41,157 | low | 0.9 | 0.1 | 1.2 | 259 | 29 | 346 |
| **Philippines** | 40,178 | low | 0.9 | 0.3 | 2 | 253 | 84 | 562 |
| **India** | 32,115 | low | 0.8 | 0.4 | 1 | 180 | 90 | 225 |
| **Sweden** | 18,423 | low | 0.6 | 0.5 | 0.7 | 77 | 64 | 90 |
| **Norway** | 15,197 | low | 0.7 | 0.6 | 0.9 | 74 | 64 | 96 |
| **Netherlands** | 44,825 | low | 0.2 | 0.1 | 0.4 | 63 | 31 | 126 |
| **Finland** | 11,278 | low | 0.7 | 0.6 | 0.9 | 55 | 47 | 71 |
| **Denmark** | 10,870 | low | 0.7 | 0.5 | 0.7 | 53 | 38 | 53 |

## Sweden

| **Country of birth** | **Population (>15 yrs)** | **Anti-HCV endemicity** | **Anti-HCV prevalence** | | | **Estimated no. of CHC cases** | | |
| --- | --- | --- | --- | --- | --- | --- | --- | --- |
|  |  |  | **%** | **Lower limit** | **Upper limit** | **Cases** | **Lower** | **Upper** |
| **Iraq** | 115,086 | high | 3.2 | 0.3 | 3.2 | 2,578 | 242 | 2,578 |
| **Former Yugoslavia** | 68,961 | high | 1.3 | 1.1 | 1.6 | 628 | 531 | 772 |
| **Thailand** | 31,136 | high | 2.7 | 1.8 | 3.7 | 588 | 392 | 806 |
| **Syria** | 25,217 | high | 3.1 | 2.5 | 3.9 | 547 | 441 | 688 |
| **Poland** | 69,711 | high | 1.1 | 0.6 | 1.9 | 537 | 293 | 927 |
| **Lebanon** | 24,165 | high | 3.1 | 2.5 | 3.9 | 524 | 423 | 660 |
| **Bosnia and Herzegovina** | 56,067 | high | 1.3 | 1.1 | 1.6 | 510 | 432 | 628 |
| **Romania** | 20,858 | high | 3.2 | 2.9 | 3.6 | 467 | 423 | 526 |
| **Russia** | 15,524 | high | 4.1 | 1.2 | 5.6 | 446 | 130 | 609 |
| **Pakistan** | 10,023 | high | 5 | 4.4 | 5.5 | 351 | 309 | 386 |
| **Turkey** | 43,691 | high | 1 | 0.7 | 1.1 | 306 | 214 | 336 |
| **Somalia** | 36,793 | high | 1 | 0.6 | 3.1 | 258 | 155 | 798 |
| **Italy** | 7,961 | high | 4.4 | 1.6 | 7.3 | 245 | 89 | 407 |
| **Chile** | 27,992 | high | 1.2 | 0.5 | 2.1 | 235 | 98 | 411 |
| **Estonia** | 9,589 | high | 3.3 | 1.6 | 4.5 | 222 | 107 | 302 |
| **China** | 22,704 | high | 1.3 | 0.4 | 2 | 207 | 64 | 318 |
| **Greece** | 12,425 | high | 1.9 | 0.5 | 2.6 | 165 | 43 | 226 |
| **Lithuania** | 7,593 | high | 2.9 | 0.7 | 3 | 154 | 37 | 159 |
| **Afghanistan** | 19,227 | high | 1.1 | 0.6 | 1.9 | 148 | 81 | 256 |
| **United States** | 15,469 | high | 1.3 | 1.2 | 2.4 | 141 | 130 | 260 |
| **Former USSR** | 6,047 | high | 3.3 | 1.6 | 4.5 | 140 | 68 | 190 |
| **Ukraine** | 5,309 | high | 3.6 | 0.9 | 4.5 | 134 | 33 | 167 |
| **Ethiopia** | 13,838 | high | 1.3 | 0.7 | 5.8 | 126 | 68 | 562 |
| **Vietnam** | 14,264 | high | 1 | 0.8 | 1.8 | 100 | 80 | 180 |
| **Morocco** | 7,963 | high | 1.6 | 0.6 | 1.9 | 89 | 33 | 106 |
| **Eritrea** | 12,061 | high | 1 | 0.6 | 3.1 | 84 | 51 | 262 |
| **Spain** | 6,730 | high | 1.7 | 0.4 | 2.6 | 80 | 19 | 122 |
| **Colombia** | 9,708 | high | 1 | 0.8 | 1.4 | 68 | 54 | 95 |
| **Brazil** | 6,019 | high | 1.6 | 1.1 | 1.6 | 67 | 46 | 67 |
| **Bangladesh** | 6,455 | high | 1.3 | 0.2 | 2.2 | 59 | 9 | 99 |
| **Croatia** | 6,330 | high | 1.3 | 1.1 | 1.6 | 58 | 49 | 71 |
| **Serbia** | 6,298 | high | 1.3 | 1.1 | 1.6 | 57 | 48 | 71 |
| **Peru** | 6,820 | high | 1.2 | 0.4 | 1.6 | 57 | 19 | 76 |
| **Bulgaria** | 6,889 | high | 1.1 | 0.3 | 2.4 | 53 | 14 | 116 |
| **Former Yugoslav Republic** | 5,705 | high | 1.3 | 1.1 | 1.6 | 52 | 44 | 64 |
| **Former Czechoslovakia** | 5,692 | high | 1.3 | 1.1 | 1.6 | 52 | 44 | 64 |
| **Sri Lanka** | 6,765 | high | 1 | 0.8 | 1.8 | 47 | 38 | 85 |
| **Finland** | 162,614 | low | 0.7 | 0.6 | 0.9 | 797 | 683 | 1,024 |
| **Iran** | 63,819 | low | 0.5 | 0.2 | 1 | 223 | 89 | 447 |
| **Denmark** | 41,144 | low | 0.7 | 0.5 | 0.7 | 202 | 144 | 202 |
| **Norway** | 39,737 | low | 0.7 | 0.6 | 0.9 | 195 | 167 | 250 |
| **Germany** | 45,637 | low | 0.5 | 0.3 | 0.9 | 160 | 96 | 288 |
| **India** | 17,868 | low | 0.8 | 0.4 | 1 | 100 | 50 | 125 |
| **Hungary** | 14,977 | low | 0.8 | 0.4 | 2.7 | 84 | 42 | 283 |
| **United Kingdom** | 19,778 | low | 0.6 | 0.4 | 1.2 | 83 | 55 | 166 |
| **Philippines** | 10,044 | low | 0.9 | 0.3 | 2 | 63 | 21 | 141 |
| **South Korea** | 9,108 | low | 0.8 | 0.2 | 2.1 | 51 | 13 | 134 |
| **France** | 7,557 | low | 0.7 | 0.5 | 0.8 | 37 | 26 | 42 |
| **Austria** | 5,541 | low | 0.5 | 0.1 | 0.7 | 19 | 4 | 27 |
| **Netherlands** | 8,112 | low | 0.2 | 0.1 | 0.4 | 11 | 6 | 23 |

## United Kingdom

| **Country of birth** | **Population (>15 yrs)** | **Anti-HCV endemicity** | **Anti-HCV prevalence** | | | **Estimated no. of CHC cases** | | |
| --- | --- | --- | --- | --- | --- | --- | --- | --- |
|  |  |  | **%** | **Lower limit** | **Upper limit** | **Cases** | **Lower** | **Upper** |
| **Pakistan** | 476,495 | high | 5 | 4.4 | 5.5 | 16,677 | 14,676 | 18,345 |
| **Nigeria** | 186,220 | high | 8.4 | 3.9 | 12.8 | 10,950 | 5,084 | 16,685 |
| **Poland** | 581,815 | high | 1.1 | 0.6 | 1.9 | 4,480 | 2,444 | 7,738 |
| **Italy** | 134,100 | high | 4.4 | 1.6 | 7.3 | 4,130 | 1,502 | 6,853 |
| **Ireland** | 456,690 | high | 1.1 | 0.7 | 1.6 | 3,517 | 2,238 | 5,115 |
| **Ghana** | 91,795 | high | 5.3 | 2.9 | 9.1 | 3,406 | 1,863 | 5,847 |
| **Egypt** | 29,145 | high | 15.7 | 13.9 | 17.5 | 3,203 | 2,836 | 3,570 |
| **China** | 271,635 | high | 1.3 | 0.4 | 2 | 2,472 | 761 | 3,803 |
| **South Africa** | 190,165 | high | 1.7 | 1 | 2.5 | 2,263 | 1,331 | 3,328 |
| **Lithuania** | 96,120 | high | 2.9 | 0.7 | 3 | 1,951 | 471 | 2,019 |
| **Bangladesh** | 206,180 | high | 1.3 | 0.2 | 2.2 | 1,876 | 289 | 3,175 |
| **Romania** | 75,950 | high | 3.2 | 2.9 | 3.6 | 1,701 | 1,542 | 1,914 |
| **Iraq** | 70,420 | high | 3.2 | 0.3 | 3.2 | 1,577 | 148 | 1,577 |
| **United States** | 163,505 | high | 1.3 | 1.2 | 2.4 | 1,488 | 1,373 | 2,747 |
| **Australia** | 115,820 | high | 1.7 | 1.2 | 2.3 | 1,378 | 973 | 1,865 |
| **Zimbabwe** | 112,545 | high | 1.6 | 1 | 9.1 | 1,261 | 788 | 7,169 |
| **Russia** | 36,415 | high | 4.1 | 1.2 | 5.6 | 1,045 | 306 | 1,427 |
| **Portugal** | 80,370 | high | 1.8 | 0.5 | 2.9 | 1,013 | 281 | 1,632 |
| **Kenya** | 136,995 | high | 1 | 0.6 | 3.1 | 959 | 575 | 2,973 |
| **Latvia** | 53,630 | high | 2.4 | 1.7 | 3.3 | 901 | 638 | 1,239 |
| **Spain** | 73,960 | high | 1.7 | 0.4 | 2.6 | 880 | 207 | 1,346 |
| **Sri Lanka** | 123,475 | high | 1 | 0.8 | 1.8 | 864 | 691 | 1,556 |
| **New Zealand** | 58,960 | high | 1.9 | 0.8 | 2.2 | 784 | 330 | 908 |
| **Thailand** | 38,480 | high | 2.7 | 1.8 | 3.7 | 727 | 485 | 997 |
| **Malaysia** | 66,060 | high | 1.5 | 0.3 | 7.7 | 694 | 139 | 3,561 |
| **Somalia** | 91,035 | high | 1 | 0.6 | 3.1 | 637 | 382 | 1,975 |
| **Turkey** | 89,170 | high | 1 | 0.7 | 1.1 | 624 | 437 | 687 |
| **Canada** | 75,760 | high | 1.1 | 0.6 | 1.3 | 583 | 318 | 689 |
| **Slovakia** | 55,765 | high | 1.4 | 0.9 | 2 | 546 | 351 | 781 |
| **Brazil** | 48,290 | high | 1.6 | 1.1 | 1.6 | 541 | 372 | 541 |
| **Greece** | 35,175 | high | 1.9 | 0.5 | 2.6 | 468 | 123 | 640 |
| **Afghanistan** | 55,335 | high | 1.1 | 0.6 | 1.9 | 426 | 232 | 736 |
| **Uganda** | 59,870 | high | 1 | 0.6 | 3.1 | 419 | 251 | 1,299 |
| **Nepal** | 46,320 | high | 1.1 | 0.7 | 1.5 | 357 | 227 | 486 |
| **Bulgaria** | 44,865 | high | 1.1 | 0.3 | 2.4 | 345 | 94 | 754 |
| **Japan** | 32,880 | high | 1.5 | 0.5 | 2.2 | 345 | 115 | 506 |
| **Singapore** | 40,005 | high | 1.1 | 0.5 | 1.7 | 308 | 140 | 476 |
| **Mauritius** | 40,375 | high | 1 | 0.8 | 1.8 | 283 | 226 | 509 |
| **Tanzania** | 35,400 | high | 1 | 0.6 | 3.1 | 248 | 149 | 768 |
| **Vietnam** | 29,575 | high | 1 | 0.8 | 1.8 | 207 | 166 | 373 |
| **India** | 684,805 | low | 0.8 | 0.4 | 1 | 3,835 | 1,917 | 4,794 |
| **Germany** | 275,055 | low | 0.5 | 0.3 | 0.9 | 963 | 578 | 1,733 |
| **Jamaica** | 157,015 | low | 0.8 | 0.2 | 1.3 | 879 | 220 | 1,429 |
| **Philippines** | 116,125 | low | 0.9 | 0.3 | 2 | 732 | 244 | 1,626 |
| **France** | 123,625 | low | 0.7 | 0.5 | 0.8 | 606 | 433 | 692 |
| **Cyprus** | 76,345 | low | 0.6 | 0.5 | 1.9 | 321 | 267 | 1,015 |
| **Iran** | 81,560 | low | 0.5 | 0.2 | 1 | 285 | 114 | 571 |
| **Hungary** | 48,575 | low | 0.8 | 0.4 | 2.7 | 272 | 136 | 918 |
| **Czech Republic** | 34,115 | low | 0.7 | 0.2 | 0.7 | 167 | 48 | 167 |
| **Netherlands** | 47,840 | low | 0.2 | 0.1 | 0.4 | 67 | 33 | 134 |

# Annex 10 Countries of birth of foreign-born migrants found amongst the ten migrant groups most affected by chronic hepatitis C in 10 or more of the 31 EU/EEA countries

| **Country of birth of migrants*** | **Number of EU/EEA countries (of 31)** | **EU/EEA countries** |
| --- | --- | --- |
| Russia | 25 | AT, BG, HR, CY, CZ, EE, FI, DE, EL, HU, IS, IE, IT, LV, LI, LT, LU, MT, NO, PL, RO, SK, SI, ES, SE |
| Italy | 20 | AT, BE, HR, DK, FI, FR, DE, HU, IS, IE, LI, LU, MT, NL, PL, RO, SK, SI, ES, UK |
| Romania | 20 | AT, BE, BG, CY, CZ, DK, DE, EL, HU, IS, IE, IT, LU, MT, NO, PL, PT, SK, ES, SE |
| Ukraine | 18 | BG, CY, CZ, EE, DE, EL, HU, IS, IT, LV, LT, MT, PL, PT, RO, SK, SI, ES |
| Egypt | 16 | AT, HR, CY, FI, FR, EL, HU, IE, IT, LI, MT, NL, PL, SK, SI, UK |
| Poland | 12 | AT, BE, CZ, DK, DE, IS, IE, NL, NO, SK, SE, UK |

*selected from the ten largest CHC affected migrant groups born in endemic countries across EU/EEA countries
